# *Caenorhabditis elegans* as a Model for Studying Nrf2/SKN-1 Activation in Alzheimer’s Disease: Plant Bioactive Compounds and Therapeutic Translation

**DOI:** 10.3390/ijms27177833

**Published:** 2026-09-01

**Authors:** Aina Bellver-Sanchis, Nikola R. Ralchev, Patrizia R. Romanska, Nikoleta K. Dyakova, Kristina I. Ivanova, Christian Griñán-Ferré, Andrey S. Marchev

**Affiliations:** 1 Department of Pharmacology, Toxicology and Therapeutic Chemistry, Institut de Neurociències, University of Barcelona, 08035 Barcelona, Spain; abellversanchis@gmail.com (A.B.-S.); christian.grinan@ub.edu (C.G.-F.); 2 Institute of Neurosciences, University of Barcelona, 08035 Barcelona, Spain; 3 Department of Immunology, The Stephan Angeloff Institute of Microbiology, Bulgarian Academy of Sciences, 1113 Sofia, Bulgaria; nikola_ralchev@microbio.bas.bg (N.R.R.); patrizia.romanska.wrk@gmail.com (P.R.R.); nikoleta.dyako@gmail.com (N.K.D.); 4 Laboratory of Eukaryotic Cell Biology, Department of Biotechnology, The Stephan Angeloff Institute of Microbiology, Bulgarian Academy of Sciences, 139 Ruski Blvd., 4000 Plovdiv, Bulgaria; k_ivanova@microbio.bas.bg; 5 Centro de Investigación en Red, Enfermedades Neurodegenerativas (CIBERNED), Instituto de Salud Carlos III, 28029 Madrid, Spain

**Keywords:** *Caenorhabditis elegans*, Nrf2/SKN-1, Alzheimer’s disease, plant bioactive molecules, therapeutic strategies

## Abstract

Alzheimer’s disease (AD) is a neurodegenerative disorder, which is characterized by several features, such as the deposition of amyloid β (Aβ) fibrils in the brain, leading to the formation of plaques and secondly tau-associated neurofibrillary tangles, resulting in the degeneration of the brain cells. Another feature of AD is mitochondrial dysfunction and oxidative stress, which are linked to the excessive production of reactive oxygen species (ROS). Under physiological conditions, ROS homeostasis is well controlled by the ROS generating system and the cellular antioxidant network. This antioxidant network is in part controlled by nuclear factor erythroid 2-related factor 2 (Nrf2), and its activation protects tissues against oxidative stress and chronic inflammation. Since oxidative stress, inflammation, and impaired proteostasis are significantly involved in the pathogenesis of AD, the KEAP1-Nrf2 system has emerged as a promising therapeutic target for the disease. *Caenorhabditis elegans* is an invaluable model organism among others for neurodegenerative disease research, due to its short life cycle, transparent body, and fully mapped nervous system and many transgenic *C. elegans* models have been developed to study different aspects of AD. This review attempts to provide comprehensive understanding of the molecular characteristics and pathological basis of AD, including the altered Nrf2-ARE signaling in AD and the molecular architecture and regulation of the Nrf2-SKN-1 pathway in *C. elegans*. An overview of plant-derived natural products and their effect on Nrf2/SKN-1 activation. Several classical and novel transgenic strains are described, translating findings from *C. elegans* to mammalian models and humans, including clinical translations and ongoing trials, as well as personalized medicinal approaches are discussed.

## 1. Introduction

Alzheimer’s disease (AD) is a neurodegenerative disorder, which is characterized by several features that are considered the main reasons for 60–70% cases of dementia, including (1) the deposition of amyloid β (Aβ) fibrils in the brain, leading to the formation of plaques and (2) tau-associated neurofibrillary tangles (NFTs), ultimately resulting in the degeneration of the brain cells [1]. It has been estimated that 55 million people worldwide suffer from AD, and a threefold increase in its prevalence is expected by 2050 [2,3]. In 2023 it was estimated that the direct costs (e.g., medical expenses, social services, and long-term care) and indirect costs (e.g., loss of productivity due to caregiving and premature mortality) were around USD 1.3 trillion, and by 2050 they are expected to reach USD 2.8 trillion [4]. The causes of the majority of AD cases, which are sporadic and typically have an age of onset above 65 years, remain unknown. However, about 1% of all cases of AD are genetic and are associated with an average age of onset in the mid-40s. These genetic cases are due to autosomal dominant mutations in one of three genes: the amyloid precursor protein (APP), presenilin 1 (PS1) and presenilin 2 (PS2), involved in the production of the small Aβ peptide. Other features of AD are mitochondrial dysfunction and oxidative stress, which are linked to the excessive production of reactive oxygen species (ROS). The accumulation of ROS in neurons causes damage to mitochondria, which, in turn, leads to more production of ROS and eventually neuronal death [2]. In high concentrations, ROS interact with cellular DNA of the nucleus and mitochondria, proteins and lipids, causing disruption of their homeostasis, ultimately leading to insufficient functioning of the antioxidant mechanism, such as oxidative stress. AD brains show an excess of ROS as well as bioactive metals such as copper, iron, zinc and magnesium, which are capable of further promoting oxidative damage and exacerbating the pathophysiology of the disease [5].

Under physiological conditions, ROS homeostasis is well controlled by the ROS generating system and the cellular antioxidant network, including several enzymes and small molecules, such as heme oxygenase 1 (HO-1), superoxide dismutase (SOD) enzymes, glutathione (GSH), catalase (CAT), thioredoxin (Trx) proteins, thioredoxin reductase (TrxR) enzymes, glutathione peroxidase (Gpx) family and NAD(P)H quinone oxidoreductase 1 (NQO1). Growing evidence indicates that this antioxidant network is being controlled by Nrf2, and its activating through Nrf2-signaling reduces oxidative stress and inflammation and protects tissues from oxidative damage. Since oxidative stress, inflammation, and impaired proteostasis are significantly involved in the pathogenesis of AD, the KEAP1-Nrf2 system has emerged as a promising therapeutic target for the disease [1,3].

Many different plants and phytochemicals have been recognized to ameliorate inflammation and oxidative stress through Nrf2 activation. Therefore, there is a great interest in natural compounds for the development of new therapeutic strategies against different pathologies. Plants and plant-based natural products have strong presence in the human diet, great biocompatibility and potential for chemical structure modification. Plants play irreplaceable roles in drug discovery, and are invaluable sources for drug candidates and leads. Plenty of natural products and ‘natural product-like’ chemicals have demonstrated potential as therapeutic agents against oxidative-stress-related diseases, such as AD, including the Nrf2 activators sulforaphane, curcumin, resveratrol, and quercetin [6,7,8,9,10]. To date, only a few natural Nrf2 activators have been approved, based on the off-target effects of the electrophilic pharmacophores in the skeleton of Nrf2 activators. Due to Nrf2’s appealing role in disease progression, a series of non-electrophilic and non-covalent synthetic Nrf2-KEAP1 protein–protein interaction (PPI) inhibitors with higher target selectivity have been investigated. Presently, the discovery of novel leading molecules from plant sources is still a hot spot [11,12,13].

*Caenorhabditis elegans* is an invaluable model organism for neurodegenerative disease research, due to its short life cycle, transparent body, and fully mapped nervous system. It enables rapid target identification and high-throughput drug screening to overcome conditions in Alzheimer’s, Parkinson’s, and Huntington’s diseases. Along with all these advantages, many transgenic *C. elegans* models have been developed to study different aspects of AD [14].

This review attempts to provide a comprehensive understanding of the molecular characteristics and pathological basis of AD, including the altered Nrf2-ARE signaling in AD and its role in neuroprotection in general, as well as the molecular architecture and regulation of Nrf2-SKN-1 in *C. elegans*. The review provides a comprehensive summary of the possible therapeutic strategies targeting Nrf2 in AD compared to the alternative treatment of AD with plant-derived natural products, emphasizing the effect of Nrf2/SKN-1 activation in AD *C. elegans* models. Several classical and novel transgenic strains are described, and translating findings from *C. elegans* to mammalian models and humans, including clinical translations and ongoing trials, as well as personalized medicinal approaches, is discussed. Finally, future prospects, such as high-throughput screening platforms and imaging, omics technologies and chronotherapeutic strategies, integration of Nrf2 activation with multi-target therapies and novel therapeutic targets and validation strategies, are highlighted.

## 2. Molecular Characteristics and Pathological Bases of Alzheimer’s Disease (AD)

### 2.1. Amyloid-Beta (Aβ) Deposition and Tau Hyperphosphorylation

Alzheimer’s disease (AD) pathology is characterized by two molecular pathways that lead to cognitive decline and brain degeneration: deposition of extracellular Aβ plaques and intracellular accumulation of excessively phosphorylated tau protein in the form of NFTs [15].

- Aβ deposition

Amyloid precursor protein is a highly conserved transmembrane protein expressed on endothelial cells, astrocytes, microglia, and neurons. Functionally, APP is important for memory, neurogenesis, neuronal development, and plasticity; however, its involvement in AD pathogenesis is better understood than its physiological functions [16]. The APP can be processed by two different pathways. First, the non-amyloidogenic pathway, which is a non-pathogenic mechanism for APP proteolysis that involves cleavage by α-secretase followed by γ-secretase. This enzymatic cleavage generates soluble extracellular fragments, sAPPα and p3, and the intracellular AICD (APP intracellular domain). Second, the amyloidogenic pathway, which is characterized by a proteolytic cleavage of APP by the β-secretase (BACE-1), generating an N-terminal soluble fragment (sAPPβ) and a C-terminal membrane-bound fragment β (CTFβ). The enzyme complex γ-secretase cleaves CTFβ, producing an extracellular Aβ fragment and an intracellular AICD [17,18]. Amyloid β monomers are soluble, but they can aggregate into different levels of organization, such as dimers, oligomers, and protofibrils. Protofibrils can further organize into β-sheet structures, such as amyloid fibrils and plaques, which are linked to AD pathology. The aggregation of Aβ monomers into these structures is dependent on the length of the monomer (Aβ40 or Aβ42) and the level of glycation [19]. Furthermore, important factors for Aβ aggregate generation that can be altered in AD include the transport of Aβ to the bloodstream and the enzymatic clearance of Aβ by enzymes such as metalloproteinases, metalloendopeptidases, insulin-degrading enzymes, and cysteine and serine proteases [20]. Elevated levels of Aβ fragments can further increase amyloid production by increasing the expression of APP [21,22].

**Figure 1:**
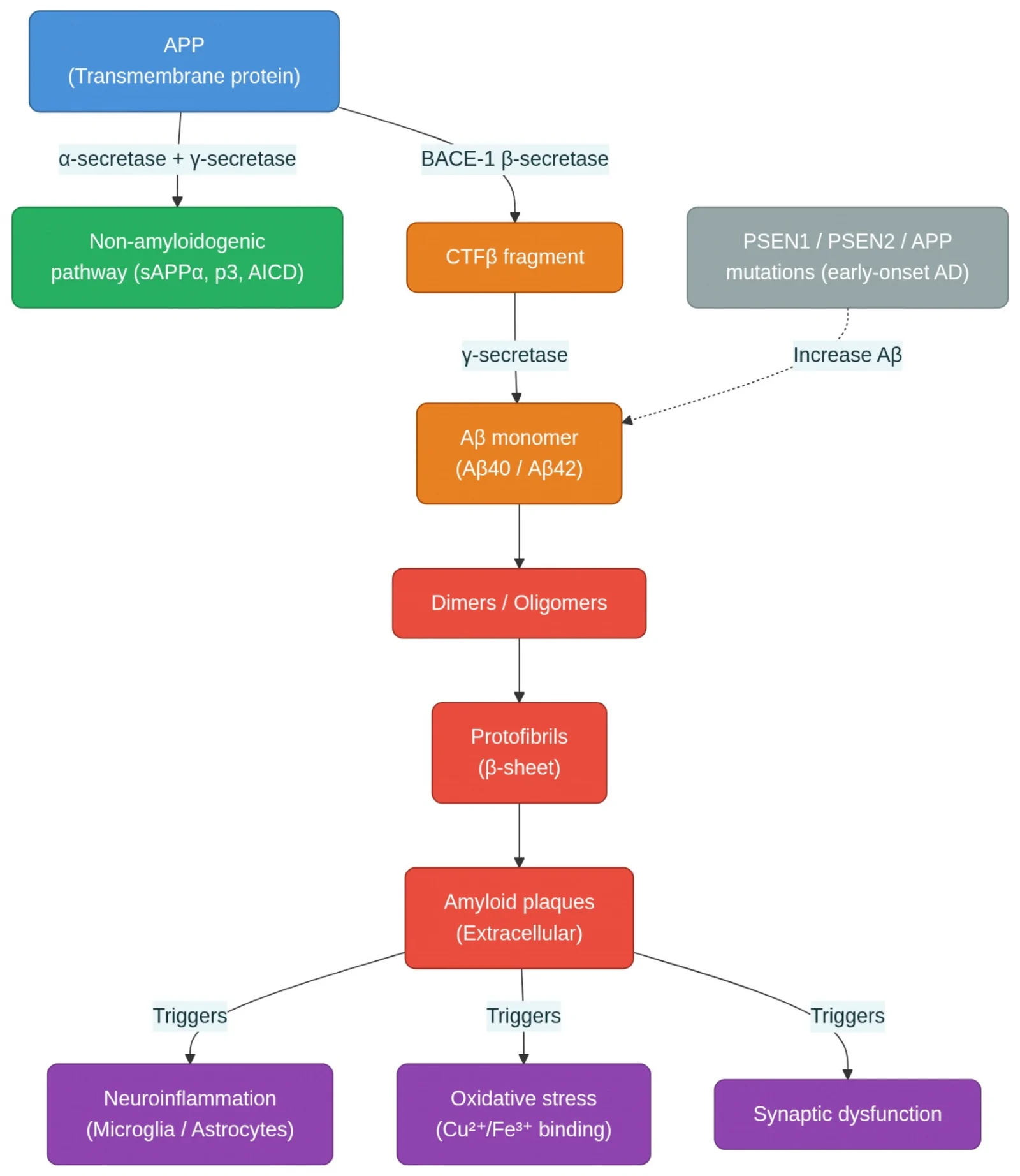
APP processing and Aβ aggregation in AD. APP is cleaved by BACE-1 and γ-secretase in the amyloidogenic pathway, generating Aβ monomers (Aβ40/Aβ42) that progressively aggregate into oligomers, protofibrils, and insoluble amyloid plaques. Plaques trigger neuroinflammation, oxidative stress, and synaptic dysfunction. Mutations in PSEN1, PSEN2, and APP increase amyloidogenic processing and are linked to early-onset AD. The non-amyloidogenic pathway (α-secretase) produces non-toxic soluble fragments. Blue: APP; green: non-toxic products; orange: amyloidogenic intermediates; red: toxic aggregates; purple: downstream damage.

Growing evidence supports the idea of Aβ plaque deposition in the brain 20–30 years before the formation of tau neurofibrillary tangles, neuronal death, and brain degeneration, indicating Aβ as a possible trigger of AD [19,23]. The role of Aβ plaques in AD is also supported by a series of genetic studies. Mutations in genes associated with increased Aβ production and plaque accumulation are linked to the development of early-onset AD, which is highly heritable. The genes associated with AD include proteins from the γ-secretase complex *presenilin 1* and *2* (*PSEN1* and *PSEN2*) and the *APP* gene [20,24,25]. The processing of APP and Aβ aggregation in AD is illustrated in Figure 1.

Accumulation of Aβ plaques shows distinct patterns of spread in the brain across the different stages of AD, with early accumulation in the cortices, allocortical regions, and midbrain in the pre-clinical stages, and in the cerebellum and brainstem during the clinical stages [19,26]. Aβ monomers and plaques can exert neuronal damage through different mechanisms, such as inflammation, induction of oxidative stress, and mitochondrial and synaptic dysfunction [18]. Microglial cells can recognize and phagocytose Aβ structures leading to their activation, secretion of pro-inflammatory cytokines, and neuroinflammation, contributing to AD pathology [17,27]. Oxidative stress can be mediated by superoxide anion generation through the binding of Cu^2+^ and Fe^3+^ by Aβ [18,28].

- Tau hyperphosphorylation

Tau protein is highly abundant in neurons and its function is to stabilize the microtubules that are essential for cellular polarity and signal transduction. Tau phosphorylation is one of the most studied modifications occurring in AD [29,30]. The structure of tau protein contains four domains: the N-terminus, the proline-rich region, the microtubule-binding domain, and the C-terminus region [31]. Microtubule stabilization is supported by the binding of the tau microtubule-binding domain to a specific pocket in the tubulin molecule, as well as the positively charged proline-rich domain of the tau molecule binding to the surface of microtubules with negative charge [29,32,33,34].

Under physiological conditions, tau is phosphorylated, modifying its affinity to microtubules and regulating their stability [31,35,36,37], and under pathological conditions, hyperphosphorylation of tau reduces its affinity to microtubules, leading to microtubule destabilization in the neurons [35,38,39]. Tau phosphorylation is mediated by three main classes of kinases: proline-directed protein kinases, non-proline-directed protein kinases, and tyrosine protein kinases. Cyclin-dependent kinase 5 (CDK5), c-Jun N-terminal kinase, and glycogen synthase kinase-3β (GSK-3β) fall into the proline-directed protein kinase group. CDK5 and GSK-3β are overexpressed in AD brains, and their inhibition reduces neuropathology and tau phosphorylation. Non-proline-directed protein kinases include adenosine monophosphate-activated protein kinase (AMPK), protein kinase B, cyclic adenosine monophosphate-dependent protein kinase (PKA), and tau protein kinase I and II (TPKI, TPKII). Fyn and Abelson family kinases and spleen tyrosine kinases are tyrosine protein kinases that phosphorylate tau [29,40,41,42].

Tau protein is natively disordered and has a high level of β-sheet formation. One of the hallmarks of AD is the development of NFTs in the neurons composed of tau protein, which is probably mediated by tau hyperphosphorylation [29,35,43,44]. Hyperphosphorylated monomeric or dimeric tau molecules can form oligomers. Tau oligomers can assemble into insoluble paired helical filaments with stacked β-strands that can further aggregate into pathological NFTs [35,45,46].

Similar to Aβ spreading in the brains of patients with AD, pathological tau follows specific distribution patterns. Consistently, tau aggregates in the entorhinal cortex, hippocampus, and neocortex [31,47]. Tau oligomers can spread through a mechanism called seeding. Seeding affects physiological tau molecules by transferring tau oligomers to nearby, relatively unaffected regions, leading to tau aggregation. Neuron-to-neuron spreading is mediated by pinocytosis or vesicle transport at the synapse [48,49,50,51]. Tau oligomers probably exert neuronal toxicity even before the formation of NFTs [49,52,53].

**Figure 2:**
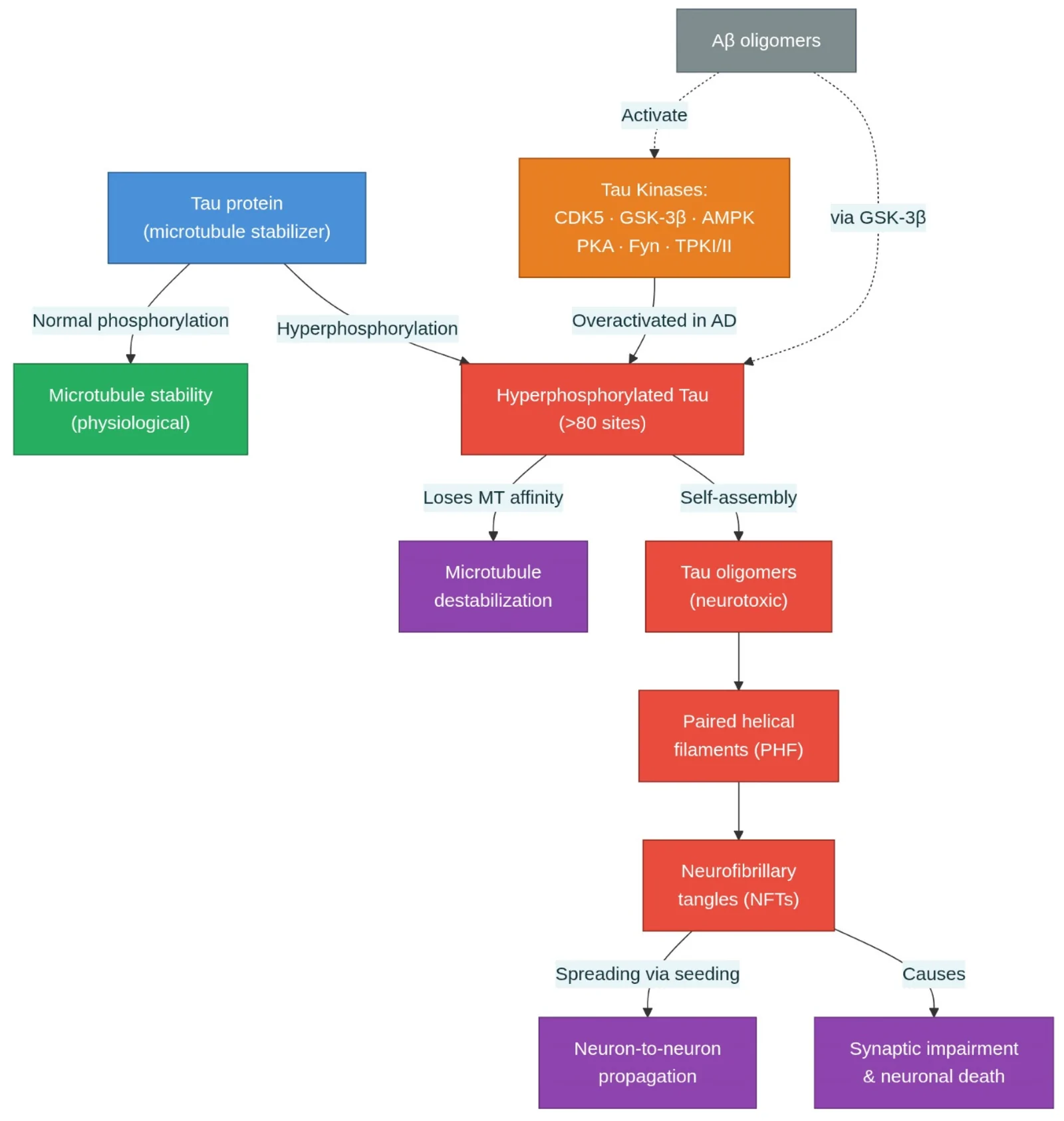
Tau hyperphosphorylation and NFT formation in AD. In AD, overactivation of tau kinases (CDK5, GSK-3β, AMPK, Fyn) hyperphosphorylates tau at >80 residues, reducing its affinity for microtubules and causing cytoskeletal instability. Hyperphosphorylated tau self-assembles into oligomers, paired helical filaments (PHF), and neurofibrillary tangles (NFTs), which spread neuron-to-neuron via seeding. Aβ amplifies tau pathology through GSK-3β activation. Blue: physiological tau; red: hyperphosphorylated tau and aggregates; orange: kinase mediators; purple: downstream neuronal damage.

Tau protein is implicated in several pathological mechanisms contributing to AD, including mitochondrial damage, synaptic impairment, and neuroinflammation [31]. The hyperphosphorylation of tau and NFT formation in AD is illustrated in Figure 2.

- Aβ and tau crosstalk

Aβ deposition and tau hyperphosphorylation are the two main hallmarks of AD; however, they are not separate pathological mechanisms. Studies have demonstrated that in AD, Aβ deposition correlates with neuronal death and brain degeneration less than pathological tau. However, the presence of NFTs is not necessarily accompanied by AD, suggesting that a combination of both proteinopathies is required for AD neurodegeneration [54,55,56,57]. The interplay between Aβ and tau can lead to a vicious cycle characterized by an increase in Aβ through hyperphosphorylated tau *via* microtubule disruption, and Aβ mediating more phosphorylation of tau [58,59]. This proposed phenomenon can hardly be explained through the direct interaction of Aβ and tau due to the presence of data showing that Aβ and tau colocalize only in 0.02% of the synapses [60,61,62]. However, in vitro studies have shown that the seeding of tau aggregates can be directly stimulated by Aβ, while Aβ can increase the resistance of tau to proteases [60,63,64].

The main Aβ–tau interplay is probably mediated through indirect mechanisms. Such a mechanism is neuroinflammation, where microglia can be activated by Aβ or tau leading to secretion of pro-inflammatory cytokines [60,65,66]. Activation of NLRP3 inflammasome can induce tau hyperphosphorylation by modulating phosphatases and tau kinases (as GSK-3) [62,67]. Aβ can also activate GSK-3 through the α2A-adrenergic receptor [60,68]. Aβ can alternatively modulate tau through regulation of neuronal activity to hyperexcitability leading to Ca^2+^ followed by signaling cascades such as protein kinases and altered gene expression [60,69].

### 2.2. Neuroinflammation

Neuroinflammation is a key player in the pathology of AD and can precede disease onset by decades [70]. Aβ plaques have been shown to be extensively surrounded by microglia and astrocytes, which secrete a number of pro-inflammatory molecules that promote oxidative stress, mitochondrial and synaptic dysfunction, and neuronal death [71,72]. Microglia and astrocytes are involved in the clearance of Aβ fibrils via phagocytosis; however, a pro-inflammatory environment in the brain can reduce clearance while perpetuating inappropriate immune activation [73]. When activated by Aβ, reactive astrocytes and microglia secrete pro-inflammatory cytokines such as IL-1, IL-6, and TNF-α, and generate ROS, which can cause substantial neuronal damage [70,72]. The inflammatory environment and increased oxidative stress can result in activation of endothelial matrix metalloproteinases (MMPs), which damage the cerebral vascular barrier, leading to reduced integrity of the blood–brain barrier (BBB) and subsequent infiltration of circulating immune cells into the brain [74].

Microglia, the resident macrophages of the CNS, are the primary immune responders to infection and tissue damage in the brain [75]. When examined within the framework of M1/M2 polarization of peripheral macrophages, microglia exhibit increased pro-inflammatory M1-like polarization in AD [76,77]. Aβ activates microglia through binding the pattern recognition receptor (PRR) dectin-1, resulting in polarization to a pro-inflammatory M1 phenotype, production of TNF-α, IL-6, and IL-1β, recruitment of microglia and peripheral macrophages, increased neuronal damage, and impaired neuronal rejuvenation [77,78,79]. Fibrillar Aβ has been shown to stimulate the induction of microglial phagocytic activity through a complex of receptors comprising TLR2, TLR4, TLR6, and TLR9, α6β1 integrin, the class A scavenger receptor (SR-A), and the class B scavenger receptor CD36 [80].

One of the primary mechanisms by which Aβ drives neuroinflammation is through the activation of the NLRP3 inflammasome in microglia via TLR-4 [81]. A principal role of NLRP3 is the mediation of the maturation of the pro-inflammatory cytokine IL-1β which initiates the upregulation of inflammatory factors that include iNOS, TNF-α, IL-6, MIP-1a and COX-2 [81,82,83,84]. NLRP3 activation involves the polymerization of the adaptor apoptosis-associated speck-like protein containing a CARD (ASC) and the assembly of ASC specks that activate caspase-1, which induces maturation of IL-1β and pyroptosis [85]. Pyroptosis is indicated by the level of its executive protein gasdermin D, which is significantly increased in AD, indicating that it is a major form of neuronal death in AD [86,87]. After pyroptosis, to further facilitate the maturation of IL-1β, ASC specks are released into the extracellular space, where Aβ_1–42_ has been found to rapidly bind to them forming Aβ-ASC aggregates, resulting in the aggregation and cross-seeding of Aβ by ASC [88,89]. Thus, the ability of immune cells to move freely in circulation, in contrast to the relatively immobile neurons, may prove to be detrimental in the case of immune dysregulation due to the potential for dissemination of Aβ by the immune system.

MMPs are zinc-dependent endopeptidases that specialize in the degradation of the extracellular matrix and basement membrane. IL-1β, along with TNF-α, upregulates the activity of MMPs in a ROS-dependent manner [90,91]. A potential mechanism for the IL-1β-induced production of ROS is through stimulation of phospholipase A_2_, which liberates arachidonic acid, an activator of NOX [92]. In addition, the presence of Aβ has been shown to induce MMP-9 and MMP-2 in activated astrocytes surrounding amyloid plaques, where they play a protective role in the breakdown and clearance of Aβ [93].

**Figure 3:**
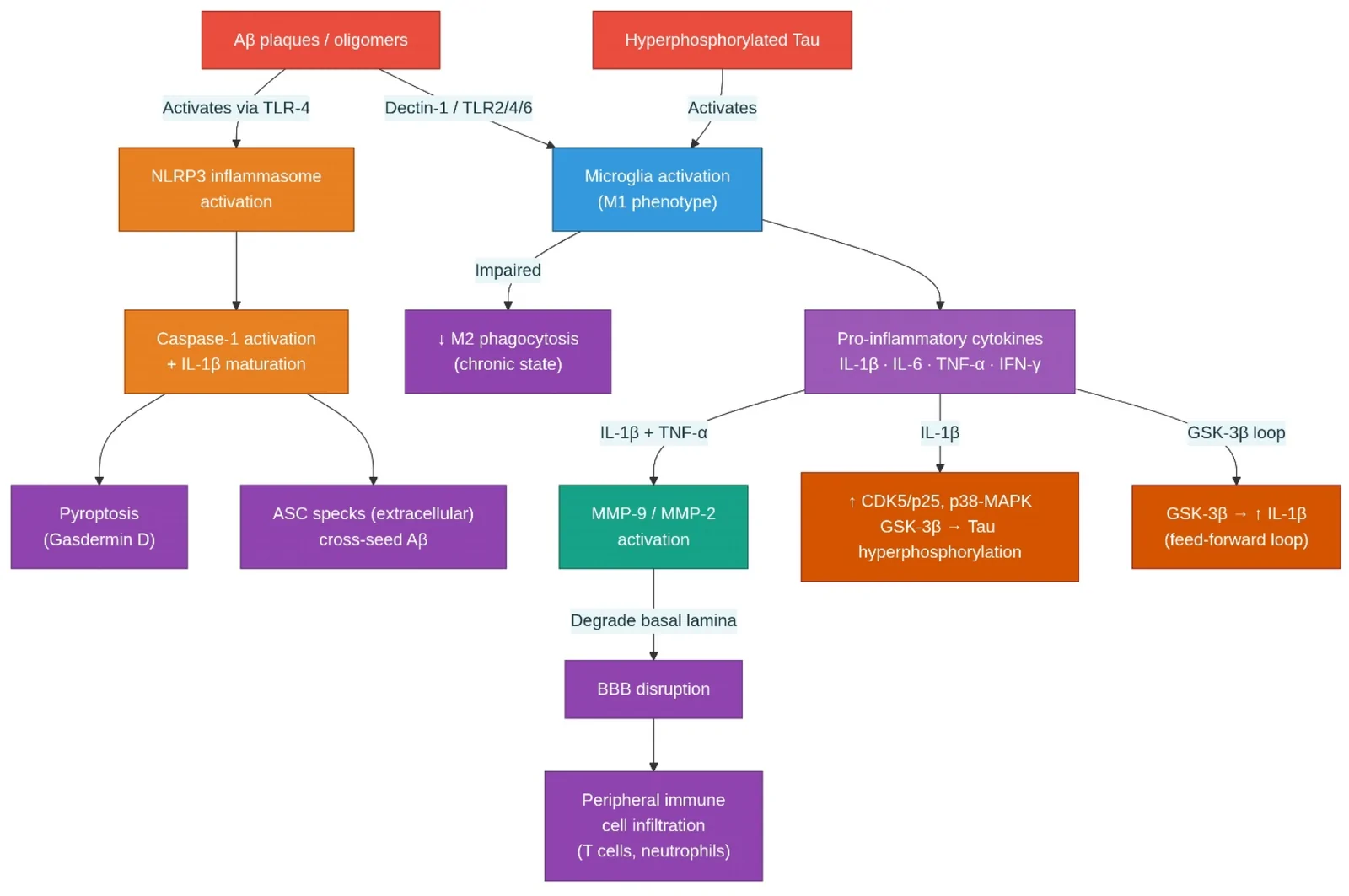
Neuroinflammation in AD. Aβ and tau activate microglia via TLR2/4/6 and Dectin-1, promoting a pro-inflammatory M1 phenotype and suppressing neuroprotective M2 polarization. NLRP3 inflammasome activation drives caspase-1–mediated IL-1β maturation, pyroptosis, and extracellular ASC speck release, which cross-seeds Aβ. Pro-inflammatory cytokines (IL-1β, IL-6, TNF-α) upregulate MMPs, thereby disrupting the BBB and enabling infiltration of peripheral immune cells. IL-1β and GSK-3β establish a feed-forward loop that exacerbates tau hyperphosphorylation. Red: triggers; blue: microglial activation; purple: cytokines; teal: MMPs; dark orange: feedback loops.

The disrupted BBB in AD, along with the secretion of chemokines by resident immune cells in the brain, leads to the infiltration of peripheral immune cells into the brain [94]. The inflammatory environment in the brain results in increased expression of vascular adhesion molecules that facilitate leukocyte trafficking. P-selectin, intercellular adhesion molecule 1 (ICAM-1), vascular cell adhesion molecule-1 (VCAM-1), and E-selectin are elevated in the serum of AD patients and undetectable in healthy individuals [95]. In the rTg4510 model of tauopathy, infiltration is evidenced by the presence of IgG, T cells and blood cell infiltrates in the brain, which exacerbates with the progression of tauopathy [96]. In the brains of AD patients, there is an accumulation of activated but not fully differentiated CD25-negative T cells, indicative of bystander activation and accumulation without antigen presentation and clonal expansion [97]. Infiltration of neutrophils into the brain parenchyma, with increased migration towards and accumulation in Aβ plaques, has been observed in a 5xFAD mouse model of AD [98]. Neutrophil infiltration and accumulation in the brain, specifically the adherence of neutrophils to capillary segments, contribute to the reduced cerebral blood flow characteristic of AD, and administration of anti-Ly6G antibodies results in significant improvements in spatial and working memory in APP/PS1 and 5xFAD mice models [99]. The process of neuroinflammation is presented in Figure 3.

### 2.3. Oxidative Stress and Mitochondrial Dysfunction

The brain has a high rate of oxygen consumption, elevated levels of polyunsaturated fatty acids (PUFAs) and transition-metal ions, and low antioxidant capacity, all of which make it particularly susceptible to oxidative stress [100]. An imbalance in the redox state due to excessive ROS production, along with an impaired antioxidant system in the CNS, lead to the increased oxidative stress observed in aging and neurodegenerative diseases [101]. Oxidative stress is induced by both radical and non-radical oxygen species generated through the partial reduction of oxygen—the primary oxygen radical superoxide O2•−, the hydroxyl radical •OH, and hydrogen peroxide H_2_O_2_ [100]. In addition, superoxide radicals react with nitric oxide, generating reactive nitrogen species (RNS), such as the peroxynitrite ONOO− ion, which contributes to further cellular stress of a nitrosative nature [100,102]. In AD patients, the presence of Aβ_1–40_ and Aβ_1–42_, impaired metal homeostasis, overexpression of oxidases, and mitochondrial dysfunction contribute to oxidative stress [103]. Among the key targets of oxidation in AD are low-density lipoprotein receptor-related protein (LRP1), which is involved in Aβ clearance, and membrane phospholipids, which contribute to the disruption of the integrity of the neuronal membrane and neuronal death [102].

Amyloid plaques have been shown to accumulate essential trace minerals, including Fe, Zn, and Cu [104]. Iron homeostasis is disrupted in AD and correlates with Aβ and tau pathologies, with NFT accumulation increasing with iron accumulation in the cortex and hippocampus of AD patients [105]. Both APP and Aβ have Cu and Zn binding sites and potentially function as Zn/Cu-buffering proteins [106]. When Aβ binds to Cu^2+^, it undergoes considerable structural changes, increasing its capacity to aggregate and its toxicity [107]. The pool of functional protein-bound Cu is reduced in AD, resulting in decreased energy production and capacity to combat oxidative stress by impairing the function of cytochrome oxidase and Cu/Zn SOD, respectively [104]. Upon the binding of Aβ to Cu(II) via a histidine bridge, a series of electron transfer reactions occur, including reduction of Cu(II) to Cu(I) and O_2_-dependent, cell-independent formation of H_2_O_2_ [108,109]. The peroxide generated as a byproduct of the reduction can further react with Aβ/Cu complexes to generate more ROS [109].

In AD, excess enzyme activity contributes to the production of ROS, including NADPH oxidase (NOX), xanthine oxidase, monoamine oxidase (MAO) and nitric acid synthase [101]. Monoamine oxidase catalyzes the oxidative deamination of monoamine neurotransmitters, generating H_2_O_2_ in the process [110]. MAO-A and MAO-B have enhanced expression in the brain tissue of AD patients compared to healthy controls [111]. Increased MAO-B activity has been found in reactive astrocytes in areas of the brain with a propensity for Aβ deposition [112]. Through the generation of free radicals, MAO can contribute to the abnormal cleavage of APP, initiating a vicious cycle of perpetual free-radical production and neuronal death.

The NOX family of oxidases is a major source of ROS in neurodegenerative diseases, as it catalyzes the formation of the superoxide anion via electron transfer to molecular oxygen [113]. In AD and PD patients, higher NOX activity and ROS production are observed, likely due to increased expression by neurons, astrocytes, and microglia [114]. An increase in NOX1 and NOX3 isoforms has been observed in AD brains, while NOX2 is involved in microglial activation and neuroinflammation, and NOX4 contributes to oxidative stress in astrocytes, ferroptosis, and NFT formation [101]. Oligomeric Aβ_1–42_ has been shown to stimulate N-methyl-D-aspartic acid (NMDA) receptors, leading to NOX activation, ROS production, and subsequent declines in synaptic plasticity and memory [115]. ROS produced by NOX leads to the activation of ERK 1/2 and the phosphorylation of cytosolic A2α (cPLA2α) phospholipase, which is one of the mediators of Aβ-induced neuronal death [115].

Mitochondrial dysfunction is one of the hallmarks of early-stage AD, as the number of intact mitochondria in AD patients is reduced, which, in addition to disrupting cellular energy production, results in impaired calcium ion homeostasis and excessive ROS generation [100,103]. Cytochrome c oxidase (COX) deficiency has been identified in the major cerebral areas of AD patients. The decline in respiratory chain efficiency reduces energy production in the brain and impairs energy metabolism, thereby negatively affecting neuronal communication and plasticity, ultimately leading to cell death and cognitive impairment [116]. With inhibition of COX function, electrons are diverted from their usual pathway into the reduction of O_2_ in the hippocampus and neocortex in AD [117]. Aβ causes mitochondrial dysfunction, swelling, and rupture by colocalizing with and inactivating Aβ-binding alcohol dehydrogenase (ABAD), which participates in the electron transport chain and redox reactions by association with the ubiquinone and NAD reduction systems [118]. In a Tg2576 mouse model of AD, soluble forms of Aβ were found to enter mitochondria and inactivate manganese superoxide dismutase (MnSOD) via nitration, resulting in excessive ROS generation [117].

The investigation of redox proteomics in AD has demonstrated that Aβ-induced oxidative stress is an early event in the pathogenesis and leads to excitotoxicity, neuroinflammation, protein misfolding and degradation, reduced synaptic plasticity, and altered signal transduction [119]. Lipid peroxidation is the first type of oxidative damage associated with Aβ, mainly evidenced by the presence of 4-hydroxy-2-nonenal (4-HNE), a highly reactive alkenal that covalently binds to brain proteins [120]. Other markers of lipid peroxidation include malondialdehyde (MDA), 2-propenal, F2-isoprostanes, and F4-neuroprostanes [100]. Proteins are subject to attack by ROS in the form of abstraction of hydrogen atoms from alpha carbons, fragmentation of the peptide backbone, and attack of amino acid chains of Arg, Lys, Pro, etc., which leads to their carbonylation and loss of structural, transport, and signal transduction functions [121]. RNS such as peroxynitrite can oxidize Cys and Met and nitrate Tyr and Trp, with 3-nitrotyrosine being a prominent marker in AD that indicates changes in the structure and function of the affected proteins and the potential for impaired signal transduction via tyrosine phosphorylation [121]. The oxidative damage of nuclear and mitochondrial DNA is implicated in early AD, playing a key role in damaging the BBB and neuronal degeneration, indicated by the presence of oxidized bases such as 8-hydroxy-2′-deoxyguanine, 8-hydroxyadenine, and 5-hydroxyuracil [102,122].

One of the most significant proteins involved in the peripheral clearance of Aβ, LRP1, is expressed in neurons, astrocytes, activated microglia, and vascular cells, and it regulates the endocytosis and lysosomal degradation of Aβ [123]. LRP1 binds Aβ on the abluminal side of the BBB, facilitating its transcytosis across the BBB into circulation, where it is cleared by LRP1 in the liver [124]. LRP1 is subject to increased oxidation in AD patients, as evidenced by elevated levels of the oxidized circulating form of LRP1, soluble LRP1 (sLRP1), which results in reduced Aβ binding affinity [125]. Thus, the oxidation of LRP1 may be one of the most significant mechanisms by which oxidative stress contributes to AD pathology.

### 2.4. Altered Nrf2 Signaling in AD

The important role of oxidative stress in AD pathogenesis brings into focus the molecular mechanisms mediating the antioxidant response. One key molecule is the transcription factor Nrf2, which initiates antioxidant response and cytoprotection [126].

There is increasing evidence of altered Nrf2 signaling pathways in neurodegenerative diseases such as AD. Nrf2 is mainly expressed in microglia and astrocytes, and to a lesser extent in neurons [5,127,128]. Data have shown that Nrf2 expression in astrocytes is decreased by half with a reduction in the number of astrocytes positive for Nrf2 in the frontal cortex of the AD human brain. A mouse model of AD (3 × Tg-AD) showed decreased numbers of Nrf2-positive astrocytes [127,129]. Furthermore, Nrf2 is primarily localized to the cytoplasm, whereas its nuclear localization is reduced in hippocampal neurons from AD patients [2,130]. The mouse APP/PS1 model of AD showed decreased Nrf2 levels in the hippocampus, with an inverse correlation with Aβ deposition [2,131]. In addition, Nrf2 deficiency in APP/PS1 mice leads to increased Aβ deposition and tau phosphorylation in the hippocampus, microglial activation, pro-inflammatory cytokine response, and impaired learning and memory capacity [126,132]. Nrf2 can modulate Aβ deposition independently of ROS by binding to the BACE-1 promoter and downregulating the transcription [127,133]. Modulation of tau pathology can be Nrf2-mediated by activation of autophagic degradation of hyperphosphorylated tau influencing the expression of nuclear dot protein 52 (NDP52) [127,134,135].

**Figure 4:**
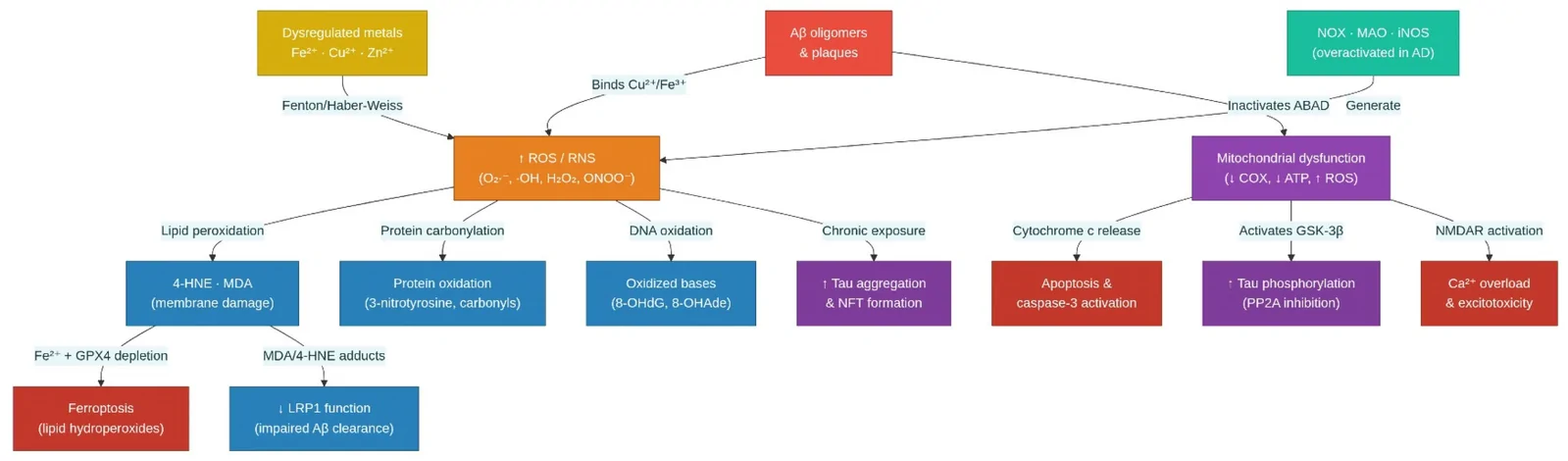
Oxidative stress and mitochondrial dysfunction in AD. Aβ binds Cu^2+^/Fe^2+^ and generates ROS/RNS via Fenton reactions, compounded by overactivation of NOX, MAO, and iNOS. Aβ inactivates mitochondrial ABAD, impairing the electron transport chain and triggering cytochrome c release and apoptosis. ROS drives lipid peroxidation (4-HNE, MDA), protein carbonylation, and DNA oxidation. GPX4 depletion enables ferroptosis through phospholipid hydroperoxide accumulation. Mitochondrial dysfunction activates GSK-3β, promoting tau phosphorylation, and induces NMDAR-mediated Ca^2+^ overload and excitotoxicity. Red: Aβ triggers; orange: ROS/RNS; purple: mitochondrial dysfunction; blue: oxidative damage; dark red: cell death.

Furthermore, a specific *NFE2L2* gene haplotype encoding the Nrf2 protein is associated with disease progression in AD patients [2,136]. Transcriptomic data support the potential role of Nrf2 in AD, as brains from Nrf2 knockout mice showed altered pathways that mimic those of brains from aged and AD patients [126,137]. Transgenic mice with a mutant human APP gene exhibit Aβ plaque deposition and suppression of Nrf2 protein expression [126,138]. Tau protein also showed the capability to decrease Nrf2 levels in an experiment with hippocampal neurons overexpressing mutant tau through modulation of the Nrf2 repressor KEAP1 [1,139]. Furthermore, another possible link between Nrf2 and AD is GSK-3β kinase, which is involved in tau hyperphosphorylation and phosphorylation of Nrf2, leading to protein degradation [127,140]. The altered Nrf2 signaling, oxidative stress and mitochondrial dysfunction in AD are illustrated in Figure 4.

## 3. Nrf2 Pathway and Its Role in Neuroprotection

### 3.1. Nrf2 Structure and Regulation. Nrf2-ARE Signaling Cascade

**Figure 5:**
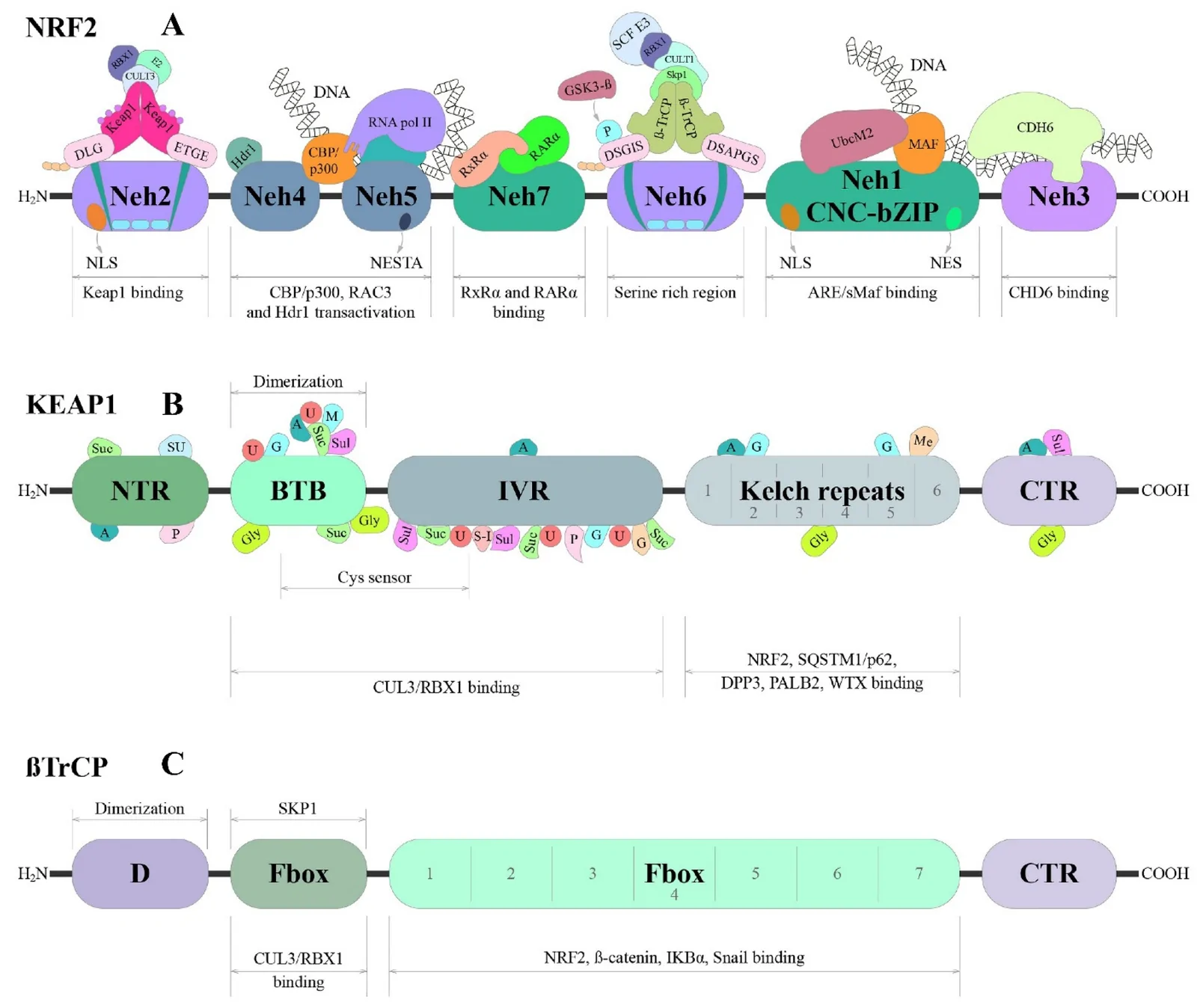
Structure of Nrf2, KEAP1, and β-TrCP. (**A**): The architecture of Nrf2 consists of seven conserved Nrf2-ECH homology (Neh) domains, Neh1-Neh7. Neh1 contains a basic leucine zipper (bZIP) motif, where the basic region is responsible for DNA binding and the ZIP dimerizes with other binding partners such as sMafs. Neh2 contains ETGE and DLG motifs, which are required for the interaction with KEAP1 and subsequent KEAP1-mediated proteasomal degradation. Neh3, 4 and 5 are transactivation domains. Neh6 contains two βTrCP degrons, DSGIS and DSAPGS, responsible for the β-TrCP-mediated proteasomal degradation. (**B**): KEAP1 contains five domains: the amino-terminal region (NTR), a broad complex, tramtrack, bric-a-brac (BTB) domain, an intervening region (IVR), six Kelch domains, and the C-terminal region (CTR). The Kelch domain and CTR mediate the interaction with Nrf2, p62, DPP3, WTX, and PALB2, which contain ETGE motifs. The BTB domain homodimerizes with KEAP1 and contributes to the interaction of IVR with Cul3/RBX1 complex. Several functionally important cysteine residues (C151, C226, C273, and C278) that sense ROS and electrophiles and modulate KEAP1-Nrf2 interaction. (**C**): The βTrCP has three domains: the dimerization domain (D), which forms homo- and heterodimers between βTrCP1 and βTrCP2, the F-box, which recruits SKP1 for the binding of CUL1/RBX1 complex, and the WD40 repeat domain, which binds βTrCP degrons DSGIS and DSAPGS in Nrf2. βTrCP, β-transducing repeat-containing protein; CUL3, Cullin3; RBX1, Ring-box protein; WD40, WD repeat protein 40; RXR α, retinoic X receptor alpha; DPP3, dipeptidyl peptidase 3; WTX, Wilms tumor gene on X chromosome; PALB2, partner and localizer of BRCA2; GSK3, glycogen synthase kinase-3; SKP1, S-phase kinase-associated protein-1.

The Nrf2 protein (encoded by the human *NFE2L2* gene) is widely expressed throughout the body and considered the master regulator of cellular redox homeostasis and antioxidant defense [141,142]. Nrf2 is a member of the basic region leucine zipper (bZIP) family of transcription factors (TFs), and in particular the cap‘n’collar (CNC) subfamily [143] together with p45 NF-E2, Nrf1 and Nrf3 [144], regulating the expression of a large network of prominent antioxidant and Phase II detoxification genes, which protect the cells from electrophilic and oxidative stress, including ROS and RNS [145]. Nrf2 is expressed in most cell/tissue types and is composed of 605 amino acids and contains seven highly conserved functional domains (named Nrf2-ECH homology), arranged as follows from its N- to C-terminus: Neh2, Neh4, Neh5, Neh7, Neh6, Neh1, and Neh3, each performing a specific function (Figure 5A) [142,146].

Neh1 has a bZIP motif, which is necessary for binding to small musculoaponeurotic fibrosarcoma (sMaf) proteins and other antioxidant-responsive element (ARE)-binding proteins. In this region, Nrf2 forms a heterodimer with sMaf proteins and binds to AREs on DNA, thus promoting transcription of Nrf2 target genes [146,147]. Along with that, Neh1 interacts with the E2 ubiquitin-binding enzyme UbcM2 (an E2-type ubiquitin-binding enzyme) to regulate Nrf2 stability and Neh1 also governs nuclear localization signal (NLS) that regulates the nuclear import of Nrf2 [141,143]. The N-terminal (Neh2) domain contains seven lysine residues and two peptide-binding motifs, DLG (low-affinity) and ETGE (high-affinity) motifs. The DLG and ETGE motifs are responsible for interaction of Nrf2 with its negative regulator Kelch-like ECH-associated protein 1 (KEAP1), while the lysine residues are important for Nrf2 subsequent KEAP1-dependent ubiquitination and proteasomal degradation through the formation of the KEAP1–Cullin3–Ring-box-1 (KEAP1-Cul3-Rbx1) complex [2,141,143,148]. The C-terminal domain (Neh3) modulates the activation of ARE-dependent genes by binding to the transcription coactivator chromo-ATPase/helicase DNA-binding protein 6 (CHD6) [143,146]. The Neh4 and Neh5 serve as transactivation domains and facilitate transcription activation (through acetylation) of Nrf2 by interacting with cAMP response element-binding protein (CREB)-binding protein (CBP) and/or receptor-associated coactivator 3 (RAC3) [144,146]. The Neh5 domain also controls the cytoplasmic localization of Nrf2 [141]. The Neh6 domain, with serine-rich residues mediates the KEAP1-independent degradation of Nrf2 by binding its DSGIS and DSAPGS motifs with β-transducing repeat-containing protein (β-TrCP) [3,146]. Neh6 also mediates Nrf2 phosphorylation by GSK-3β [149]. The Neh7 domain has a suppressive role in Nrf2 transcriptional activity through a physical association with the retinoid X receptor α (RXRα), thus contributing to inhibition of the Nrf2-ARE signaling pathway. The RXRα receptor inhibits the binding of CBP to the Neh4 and Neh5 domains, resulting in decreased expression of Nrf2 target genes [143,146]. The continual degradation of Nrf2 ensures that only a fraction of newly synthesized Nrf2 enters the nucleus to control baseline gene expression, while the expression of Nrf2 is tightly regulated by KEAP1 [141]. The human KEAP1 protein is a cysteine-rich protein consisting of 624 amino acid residues with an apparent molecular weight of 69.7 kDa (Figure 5B). It consists of five domains: the N-terminal region (NTR, 1-60); the broad-complex, tramtrack, bric-a-brac (BTB, 61-179) domain; the central intervening region (IVR, 180-314), also called the BACK domain with a nuclear export signal (NES) regulating the cytoplasmic localization of KEAP1; six Kelch repeats, known also as the double-glycine repeat (DGR, 315-598) domain; and a carboxy-terminal domain (CTR, 599-624). The human KEAP1 contains 27 cysteine (Cys) residues, but only Cys151, Cys226, Cys273, Cys288, Cys434, and Cys613 are critical for Nrf2 activation [3]. In addition to KEAP1, at least three other systems regulate Nrf2 proteasomal degradation: the β-TrCP-Cul1/Rbx1 E3 ligase complex, the WD repeat-containing protein 23 (WDR23)-DDB1-Cul4 E3 ligase complex, and the E3 ubiquitin ligase HMG-CoA reductase degradation 1 homolog (HRD1; Figure 5C).

**Figure 6:**
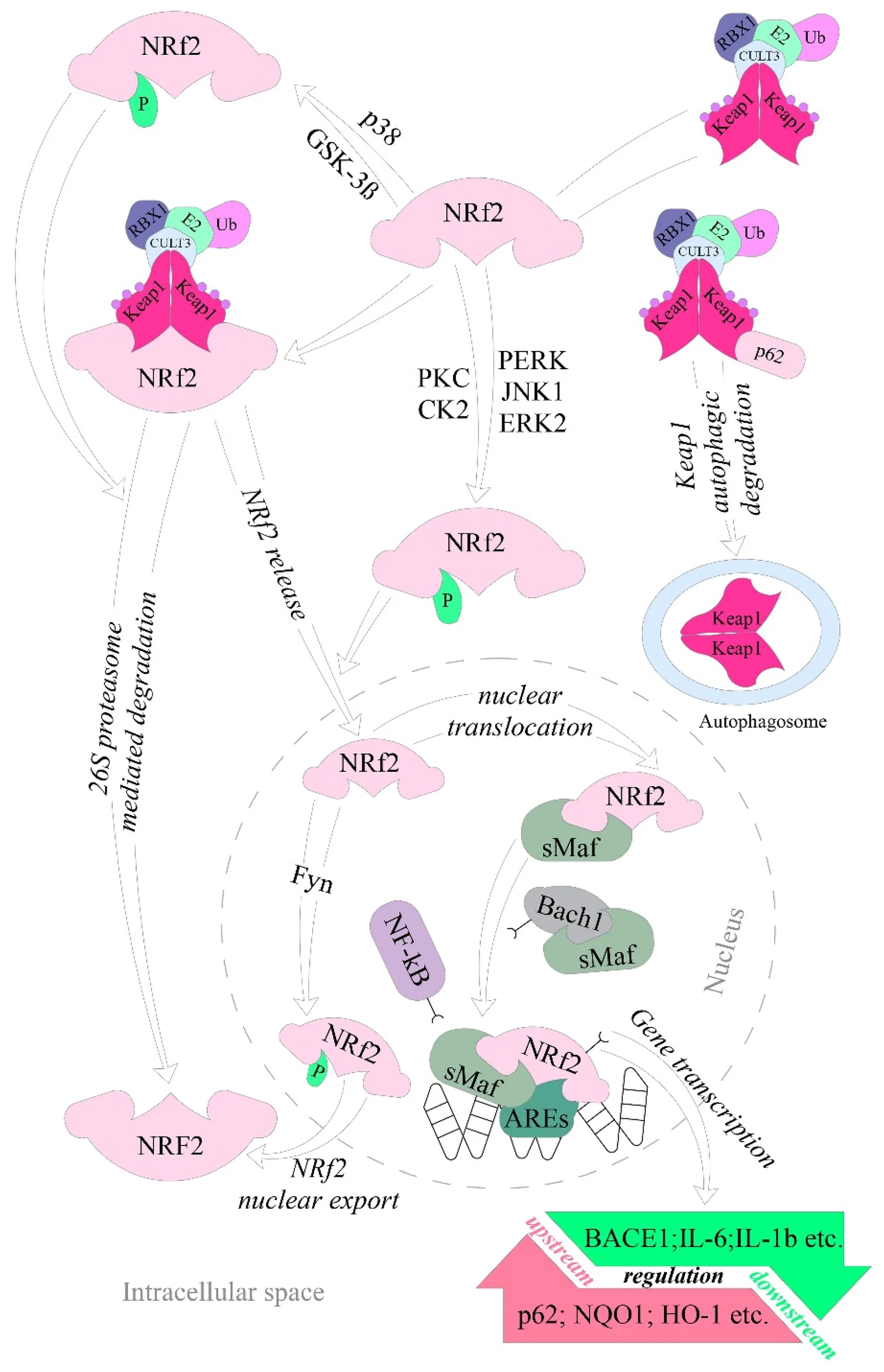
Mechanisms of KEAP1-dependent and independent regulation of Nrf2. Under normal physiological conditions, the KEAP1-Cul3-E3 ubiquitin ligase typically targets multiple lysine residues in the Nrf2 Neh2 domain, located between the DLG and ETGE motifs, thereby facilitating ubiquitination that subsequently leads to degradation by the 26S proteasome. The p62 causes autophagic degradation of KEAP1, which may increase the Nrf2 accumulation in the nucleus. Upon exposure to ROS and electrophilic stress, specific cysteine residues in KEAP1 undergo modifications, resulting in structural alterations in the KEAP1-Cul3-E3 ubiquitin ligase complex. This disrupts the ubiquitination of Nrf2, prompting its translocation to the nucleus where it binds to AREs in target genes through heterodimerization with the Maf protein. Consequently, this initiates a cascade of gene expression that serves to protect the cell. GSK-3β and p38 MAPK can phosphorylate Nrf2 at the Neh6 domain and favor its degradation. Oppositely, the Nrf2 phosphorylation mediated by PKC, CK2 PERK, JNK1, and ERK2 facilitate its dissociation from KEAP1 and consequent nuclear accumulation. In the nucleus, Nrf2 forms a heterodimer with transcriptional co-activator Mafs to facilitate a stable binding to ARE and enhance the gene transcription. Fyn kinase may phosphorylate the Nrf2 at Tyr568 and favor its nuclear export. Bach1 competes with the Nrf2 in binding to ARE leading to decrease of Nrf2 transcriptional ability. The NF-κB also competes with Nrf2 in binding to ARE and downregulates its transcriptional activity.

The intracellular concentration of Nrf2 is determined by a complex equilibrium between its synthesis and its proteasomal degradation [143]. There are three known regulatory pathways that degrade Nrf2 using ubiquitin. The first and most common is mediated by KEAP1. The second is through GSK3 and β-TrCP-dependent Cul1-based ubiquitin ligase complex. The β-TrCP recruits Cul1 and dimerizes to facilitate binding to Nrf2. However, GSK-3β is required to phosphorylate the DSGIS site to initiate ubiquitination. Lastly, the HRD1 complex, an E3 ubiquitin ligase, binds when there is endoplasmic reticulum stress (Figure 6) [147]. Under unstressed conditions, Nrf2 is bound to its repressor KEAP1, a major negative regulator of Nrf2 [143,146]. KEAP1 relies on its BTB domain (located at the N-terminal part of KEAP1) to form a homodimer, which then binds to the ETGE and DLG motifs of Nrf2 via the Kelch domains of KEAP1 [1,146]. KEAP1-Nrf2 combines with Cul3/Rbx1-based E3-ubiquitin, which is responsible for the continuous ubiquitination and subsequent degradation of Nrf2 by the 26S proteasome (Figure 6) [143,146].

Under physiological conditions, the half-life of Nrf2 is 10–30 min due to the KEAP1-mediated Nrf2 degradation [143]. In stress-inducing conditions (oxidative or electrophilic stress), Nrf2 is activated [141] and specific stress-sensing cysteine residues in KEAP1 are modified [150], leading to conformational changes of KEAP1 without Nrf2 dissociation from KEAP1 [146]. In particular, these modifications depend on cysteine sulfhydryl groups, especially Cys151, Cys273, and Cys288 of KEAP1 [150]. This process results in inactivation of KEAP1 and stabilization of Nrf2, thus blocking its ubiquitination and degradation [144]. As Nrf2 remains bound to KEAP1, but cannot be ubiquitinated and released for degradation, free KEAP1 is not generated and newly synthetized Nrf2 is translocated into the nucleus, where it heterodimerizes with sMaf and binds to regulatory ARE sequences. Consequently, the expression of more than 250 genes encoding proteins involved in multiple processes to maintain cellular homeostasis, including antioxidant and detoxifying genes, is promoted, and downregulation of the production of pro-inflammatory mediators is observed [141,144]. ARE sequences (5′-RTGACnnnGC-3′) are present in the promoters of different genes (antioxidant, NADPH synthesis, stress and metal-binding, detoxification, protein turn-over, and several genes that control cellular processes such as DNA repair and apoptosis prevention), most important of which are glutamate–cysteine ligase catalytic (GCLC) and modifier (GCLM) subunits, NQO1, heme oxygenase 1 (HMOX1), sulfiredoxin1 (SRXN1), glutathione S-transferase (GST), multidrug resistance-associated proteins (MRPs), and UDP glucuronosyltransferase (UGT). The presence of ARE in the HMOX1 promoter allows for the Nrf2-mediated synthesis of the corresponding protein, heme oxygenase (HO-1), the enzyme that catalyzes the initial and rate-limiting step in heme degradation [141,147].

To date, there are two models explaining the regulation of Nrf2 stability in the KEAP1-dependent regulation of Nrf2, of which the “hinge and latch” model is the most preferred one for understanding the KEAP1-Nrf2 interaction. According to this model, the Nrf2 ETGE motif (the high-affinity site) first binds to KEAP1, acting as a hinge. The ETGE motif adopts a β-turn conformation that inserts deeply into the KEAP1 pocket with a buried surface area of 420 Å^2^. Nrf2 then binds through its DLG motif of Neh2 in a second independent binding site (the low-affinity site) of KEAP1, acting as a latch [3,141,142]. Another form of KEAP1-dependent (autophagic stabilization) regulation of Nrf2 is via p62, a protein also known as sequestosome-1 (SQSTM1), which can bind to both polyubiquitinated proteins and the autophagic machinery, thereby targeting specific cargoes for autophagy [151]. The p62 protein (a competitor of Nrf2 for binding to KEAP1) disrupts the KEAP1-Nrf2 system by binding to KEAP1 via an STGE motif on p62, which is similar to Nrf2’s ETGE. High expression of p62 results in increased levels of Nrf2 (Figure 6) [152]. Indeed, p62 directly interacts with KEAP1 to sequester it into aggregates and promote the translocation of Nrf2 into the nucleus. Here, the Nrf2 promotes the expression of many genes, including p62, with a positive loop of reinforcement [150].

In addition to KEAP1-dependent regulation, many research groups have addressed KEAP1-independent regulation at the level of post-translational modifications of Nrf2, including phosphorylation, ubiquitination, and acetylation [3,143]. Several transcriptional factors (TFs), such as the aryl hydrocarbon receptor (AhR), regulate the expression of the *NFE2L2* gene, which encodes Nrf2. This TF recognizes xenobiotic response element (XRE) sequences in the *NFE2L2* promoter and induces Nrf2 transcription; AhR ligands, e.g., uremic toxins and xenobiotics, upregulate Nrf2 expression [153]. The BTB and CNC homology 1 (BACH1) TF is a negative regulator, an Nrf2 competitor and a molecular sensor of intracellular heme; it competes with Nrf2 for its binding sites on DNA. BACH1 is a Maf-binding transcriptional repressor (a part of bZIP TF); it forms heterodimers with small Maf (MafK and MafG) proteins on DNA, leading to the repression of ARE-mediated gene expression and induction (Figure 6) [141]. Both Bach1/Maf and Nrf2/Maf competitively share the same binding site (the cis-element of the ARE). Accordingly, repression or activation of target genes may occur [3]. The nuclear factor kappa B (NF-κB), c-Jun and c-Fos, as well as components of the Notch and phosphatidylinositol 3-kinase (PI3K)/protein kinase B (Akt) signaling pathway, activate *NFE2L2* transcription, mediating the increase in Nrf2 expression in response to inflammatory stimuli [154,155,156], while the tumor suppressor p53 inhibits Nrf2-dependent transcription of antioxidant genes [157]. For the translational regulation of Nrf2, an internal ribosomal entry site (IRES) at the 5′ untranslated region (UTR) of the Nrf2 mRNA is needed to initiate its internalization into ribosomes for protein synthesis. The presence of ROS increases Nrf2 translation in an IRES-dependent manner by promoting the entry of the mRNA into ribosomes, which means that Nrf2 is markedly increased under oxidative stress, and its translational efficiency is low under basal conditions [158,159].

Nrf2 may be regulated by epigenetic mechanisms, including DNA methylation, histone modification, and microRNAs. For example, miR-144 has been associated with Nrf2 modulation in retinal, alveolar or neuronal damage [160,161,162] and acts as a negative modulator of Nrf2. The direct binding of miR-144 to the UTR of Nrf2 mRNA decreases Nrf2 levels in K562 lymphoblasts and primary erythroid progenitor cells [163]. Other microRNAs related to decreased Nrf2 levels and the expression of antioxidant proteins include miR-93, miR-28 [164,165], *miR-27a*, *miR-142-5p*, and *miR-153*, which repress Nrf2 mRNA in neuronal cells [166]. On the other hand, *miR-27a*, *miR-142-5p*, and *miR-153* repress Nrf2 mRNA in neuronal cells [166].

One of the mechanisms involved in Nrf2 post-translational regulation is mediated by GSK-3 (β-TrCP-dependent pathway). GSK3β plays a key role in regulating Nrf2 by promoting its degradation through phosphorylation of Nrf2 at serine residues within the DSGIS motif of the Neh6 domain. This phosphorylation creates a phosphor-degron, which is recognized by the E3 ubiquitin ligase adapter β-TrCP, leading to Nrf2 ubiquitination and subsequent proteasomal degradation (Figure 6) [1]. β-TrCP is an adaptor for the Skp1-Cul1-Rbx1-F-box protein (SCF) E3 ubiquitin ligase complex, and therefore Nrf2 phosphorylation by GSK-3 allows Nrf2 to be recognized by β-TrCP, thus facilitating subsequent Nrf2 degradation. Other kinases, including extracellular signal-regulated kinase (ERK), C-JunN-terminal kinase (JNK), PKR-like endoplasmic reticulum kinase (PERK), phosphatidyl inositol 3-kinase (PI3K), and protein kinase C (PKC) inhibit GSK-3 and promote nuclear import of Nrf2 [167], while p38 mitogen-activated proteinkinase (p38) phosphorylation induces Nrf2 degradation in the cytoplasm [168].

The Nrf2 modulation may also be mediated by the E3 ubiquitin ligase Hrd1 (also called SYVN1). Nrf2 interacts with the C-terminal domain of Hdr1 through its Neh4 and Neh5 domains, which induce Nrf2 ubiquitination and degradation, independent of KEAP1 and β-TrCP. Inhibition of Hdr1 or deletion of its gene prevents Nrf2 loss, suggesting that the inhibition of Hdr1could be a promising therapeutic target [169].

### 3.2. Downstream Targets of Nrf2 Activation

Over the past few decades, many studies have revealed the major role of Nrf2 as a TF for antioxidant stress response and drug detoxification. Nrf2 induces the expression of ARE-containing cytoprotective genes in response to cell stress (mainly ROS or RNS), which forms a network of cooperating enzymes involved in Phase I, II, and III drug detoxification reactions and elimination of pro-oxidants to maintain cellular homeostasis. Phase I enzymes mediate oxidation, reduction, and hydrolytic reactions of xenobiotics, including NQO1, carbonyl reductases (CBRs), aldo-keto reductases (AKRs), aldehyde dehydrogenase 1 (ALDH1), and certain cytochrome P450 oxidoreductases (CYPs) [170]. Phase II enzymes traditionally refer to the enzymes catalyzing the conjugation reactions, such as GST, UDP-glucuronosyltransferase (UGT), UDP-glucuronic acid synthesis enzymes, and HO-1 [170,171,172]. Phase III enzymes transport the conjugated metabolites after Phase II and are mainly drug efflux transporters, such as multidrug resistance-associated proteins (MDR), breast cancer resistance protein (BCRP), ATP-binding cassette g5 (ABCG5) and g8 (ABCG8) [170,172]. General antioxidant enzymes include the reduced glutathione (GSH) production, utilization, and regeneration influenced by directly regulating two subunits of the GCLC and modifier GCLM subunits involved in glutathione biosynthesis [173]. Other Nrf2 targets are the redox-cycling enzymes thioredoxin, thioredoxin reductase, sulfiredoxin, peroxiredoxin, glutathione peroxidase, superoxide dismutase 1 (SOD1), and catalase (CAT), and severalGSTs, which are the enzymes mediating the elimination of ROS [170,174]. These comprehensive cytoprotective proteins encoded by Nrf2-target genes are essential for protection against a variety of toxic and oxidative insults and therefore many diseases that have oxidative stress as underlying pathological features, including cardiovascular disease, metabolic syndrome, neuronal degeneration, autoimmune disorders, and cancer [170]. Some of the target genes regulated by Nrf2 are presented in Table 1.

Along with drug metabolism and antioxidant defense, Nrf2 also regulates genes related to metabolic reprograming (aerobic glycolysis and glycogen synthesis, pentose phosphate pathway, *de novo* purine biosynthesis, amino acid metabolism, lipid metabolism, and heme and iron metabolism), proteostasis, autophagy, mitochondrial physiology and biogenesis, as well as inflammation and immunity [170,173,175,176].

## 4. Therapeutic Strategies Targeting Nrf2 in AD

**Figure 7:**
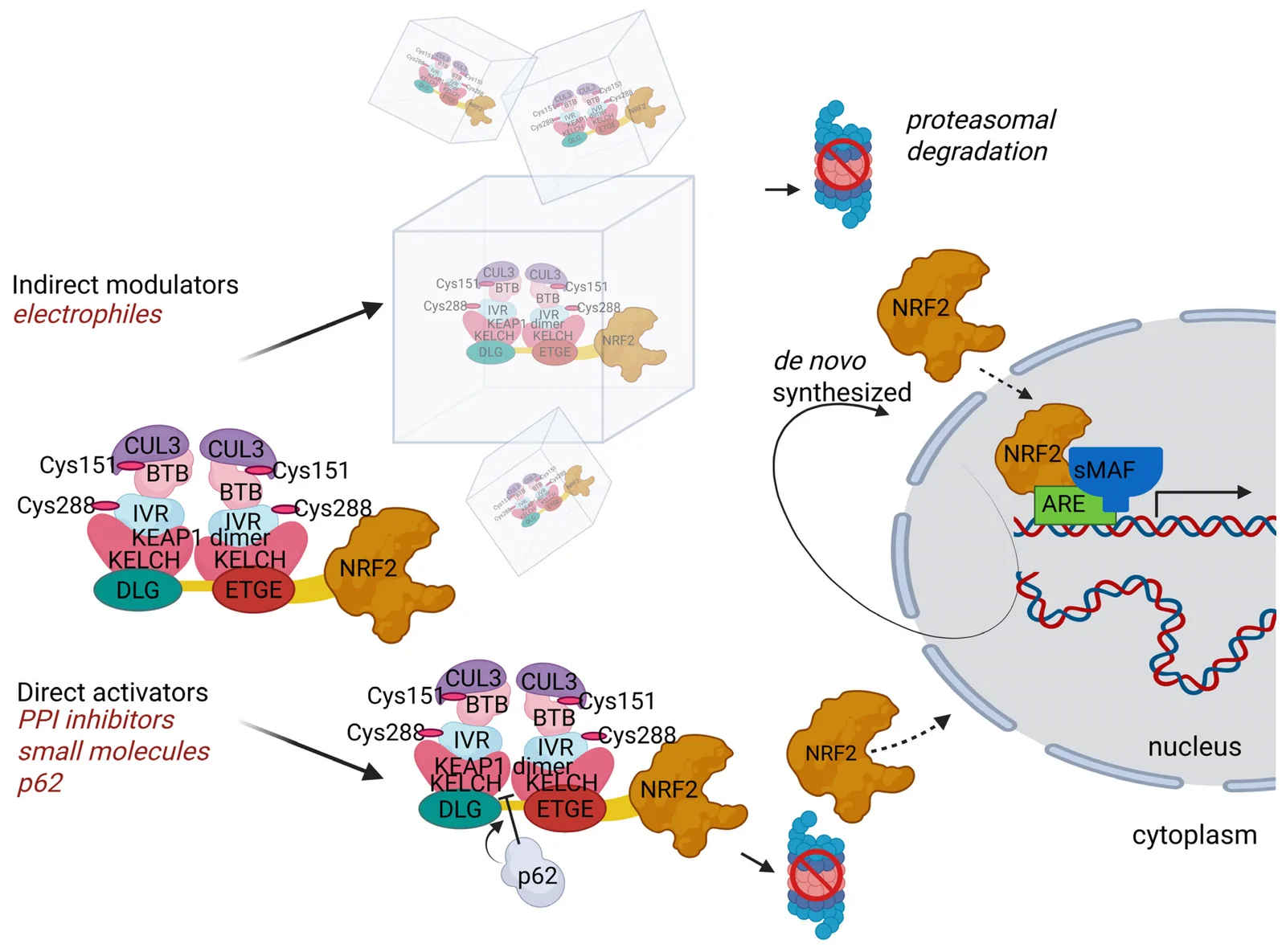
The figure illustrates the hinge–latch and conformational cycling models by which direct and indirect agents can influence the KEAP1-Nrf2 interaction, allowing for Nrf2 activation and targeted gene regulation. Structurally, the distinct kinetics of ETGE and DLG binding underpin both models [198,200]. The ETGE motif binds KEAP1 tightly and stably, whereas the DLG interaction is rapid and reversible, consistent with the transient open and closed conformations observed in live-cell studies [179,180,181]. Acting directly or indirectly as a modifier of the KEAP1-Nrf2, the mentioned interaction can cause perturbations, resulting in slowed KEAP1 regeneration, allowing newly synthesized Nrf2 to escape degradation. The direct impact of PPI inhibitors, such as peptides or small molecules, on the KEAP1-Nrf2 interaction also leads to the accumulation of free Nrf2, as the protein complex is disrupted by competitive binding [134]. Thus, the programmed degradation of Nrf2 is avoided, and its activation is promoted. Abbreviations: KEAP1, Kelch-like ECH-associated protein 1; BTB, broad complex, tramtrack and bric-à-brac; IVR, intervening linker region; Nrf2, nuclear factor erythroid-derived 2-related factor 2; Cul3, Cullin 3 ligase; sMaf, small musculoaponeurotic fibrosarcoma; ARE, antioxidant responsive element.

Therapeutic strategies aimed at modulating the Nrf2-KEAP1 pathway in AD exploit the rich structural and kinetic complexity of this regulatory system [177,178]. To enhance Nrf2 activity, approaches operate either directly by interfering with KEAP1-Nrf2 binding or indirectly by acting on KEAP1, both converging on the subsequent alteration of KEAP1’s ability to ubiquitinate Nrf2 [179]. This distinction is mechanistically important because direct activators act primarily on the physical hinge–latch interface between KEAP1 and Nrf2, whereas indirect activators reshape the conformational cycling of the KEAP1-Nrf2-CUL3 complex (Figure 7) [180,181]. Both perspectives are necessary to understand why such a broad and chemically diverse Nrf2 “drugome” [177,178,182] exists and why different classes of compounds produce similar biological outcomes through conceptually different routes.

The Nrf2-KEAP1 axis is widely regarded as a master regulator of cellular defense against oxidative and electrophilic stress; however, its function extends beyond redox homeostasis [178,183]. Nrf2 and KEAP1 participate in large protein networks that influence mitochondrial function, cytoskeletal organization, transcriptional control, and inflammatory signaling [182,184]. These layers of redundancy and cross-talk explain why experimental outcomes in Nrf2 research are often context-dependent and why simplistic interpretations of Nrf2 activation can be misleading, particularly in chronic neurodegenerative conditions such as AD [147,177,185].

Therapeutic engagement of Nrf2 in AD spans KEAP1 dependent and independent Nrf2 regulatory mechanisms [186,187], including covalent inducers, such as electrophilic small molecules, both synthetic and naturally derived [150], as well as PPI inhibitors or microRNAs [188,189,190,191,192] that hinder KEAP1-Nrf2 interplay. Indirect strategies also include the inhibition of Nrf2 repressors, such as BACH1 [127,193], modulation of upstream signaling pathways involving kinases and p62/SQSTM1 [147], and combination approaches that integrate Nrf2 activation with anti-amyloid [194,195], anti-tau [196], autophagy, mitophagy, or anti-inflammatory therapies [197]. These diverse strategies converge on Nrf2 activation but differ fundamentally in their engagement with the KEAP1-Nrf2 regulatory machinery.

- Hinge–Latch or conformational cycling

The regulation of Nrf2 by KEAP1 has been described by several complementary models, most notably the hinge–latch and conformation cycling models (Figure 7) [198]. Both models emphasize that Nrf2 is not simply released from KEAP1 upon activation; rather, it accumulates because ubiquitination is impaired while *de novo* synthesis continues. The classical hinge–latch model proposes that Nrf2 binds to KEAP1 through two motifs in its Neh2 domain: the high-affinity ETGE motif, which acts as a hinge, and the lower-affinity DLG motif, which acts as a latch [198,199,200]. The conformational cycling model reframes hinge–latch interactions as dynamic states rather than static binding events [180]. In this model, the KEAP1-Nrf2 complex continuously cycles between open and closed conformations, with ubiquitination occurring only in the closed, ubiquitin-competent state [181]. Electrophilic Nrf2 inducers do not dissociate KEAP1 from CUL3 or Nrf2, as shown by fluorescence recovery after photobleaching experiments combined with lifetime imaging microscopy [180], but instead induce conformational changes in KEAP1 that misalign Nrf2 lysine residues relative to the E2 enzyme [180,199,201]. This prevents ubiquitination while preserving KEAP1-Nrf2 binding, leading to the accumulation of Nrf2 in a non-ubiquitinatable closed complex [181].

Support for this model comes from NMR spectroscopy experiments and mathematical modeling, which demonstrate that class I-V electrophiles such as CDDO or sulforaphane modify KEAP1 cysteines without disrupting DLG binding, reinforcing the idea that indirect activators act through conformational effects rather than physical disengagement [179,180,202,203]. In contrast, non-electrophilic PPI inhibitors, termed class VI compounds, bind to the Kelch domain of KEAP1 and disrupt DLG and potentially ETGE binding, thereby shifting the equilibrium away from the ubiquitin-competent state. Endogenous competitors, such as phosphorylated p62, which contains a DPSTGE motif, can outcompete DLG in the open complex, still supported by the ETGE motif interaction, further illustrating how modulation of cycling dynamics, rather than dissociation, governs Nrf2 de-repression and inhibits its ubiquitination in the KEAP1:Nrf2 closed complex [180].

### 4.1. Non-Electrophilic Nrf2 Activators

Non-electrophilic Nrf2 activators primarily target the KEAP1-Nrf2 interface itself and therefore align most naturally with the hinge–latch concept [204]. To be able to interfere in such a way, PPI inhibitors are either peptide-based or possess a specific chemical feature accommodated to achieve better efficacy [205]. While simulations indicate that electrophilic activators require threshold concentrations for robust Nrf2 induction, non-covalent PPI disruptors offer more precise and tunable control [183,202,203,206,207,208]. Although protein–protein interactions are traditionally challenging drug targets, the KEAP1-Nrf2 interface is unusually amenable to modulation due to its arginine-rich pocket [207].

Small molecule PPI design strategies are based on multiple hit compounds with diverse scaffolds that interact with the Kelch domain [205]. They can be distinguished further into the following classes: tetrahydroisoquinoline (THIQ), 1,4-diaminonaphthalene, 3-phenylpropanoic acid, 1-phenylpyrazole and fluorenone carboxamide analogs, and others [208,209]. Structure-based virtual screening and fragment-based design have identified small molecules that can establish several H-bonds and π-π stacking interactions engaging key arginine residues within the KEAP1 Kelch domain, leading to non-covalent disruption of Nrf2 binding and activation of Nrf2 signaling with low toxicity [207].

PPI inhibitors with a domain-binding peptide pattern mediate their interaction by possessing a linear sequence of residues that can interact with the Kelch domain of KEAP1 [210,211]. Their relatively small interface is amenable to ligand intervention and peptide truncation, which can improve their drug-like properties, such as molecular weight, permeability, and specificity [211]. Current efforts have focused mainly on the high-affinity ETGE site, often resulting in poor drug-like properties, whereas targeting DLG may improve both potency and pharmacokinetics, particularly for brain-directed therapies [208]. Thus, pharmacologically used compounds such as NG262 [202] and PRL295 [177] preferentially interfere with KEAP1-DLG binding. Other tactics involve protein-like polymers, which have shown effective BBB permeability and Nrf2-ARE activation in various in vitro assays, demonstrating their potential for neurodegenerative disorders therapy. Their design and complex multimodal testing, based on PPI optimization by the addition of arginine residues coupled to their lipophilic polymer backbone, allowing for permeability enhancement and endosomal degradation protection, provides a promising improvement for their KEAP1 engagement [212].

Proteolysis-targeting chimeras (PROTACs) provide an alternative means of modulating Nrf2 signaling by targeting KEAP1 [213]. KEAP1-recruiting PROTACs elevate Nrf2 levels by promoting KEAP1 degradation, resulting in sustained antioxidant response. However, the identification of selective KEAP1 ligands remains challenging due to KEAP1’s central role in cellular homeostasis. Although Nrf2-derived peptide inhibitors offer high specificity, their poor permeability limits utility. In contrast, the non-covalent small molecule 3-phenylpropanoic acid derivative KI696 [177,213], identified via fragment-based screening, binds the KEAP1 Kelch domain with nanomolar affinity and selectively disrupts the KEAP1-Nrf2 interaction without off-target covalent effects, making it a promising scaffold for KEAP1-recruiting PROTAC development [213]. The development of drug-like non-covalent KEAP1-Nrf2 inhibitors faces challenges due to the size and polarity of the Kelch binding pocket. For that reason novel non-covalent KEAP1-Nrf2 inhibitors have been developed from a weak fragment hit identified by crystallographic screening. The used strategy led to the discovery of novel compounds with low nanomolar affinities and complete selectivity for KEAP1 in a panel of homologous Kelch domains. The compounds activated the expression of Nrf2-controlled genes and showed anti-inflammatory effects by downregulating NLRP3 inflammasome and STING signalling activation and upregulating the activation of cytoprotective pathways and a different profile from typical covalent Nrf2 activators [214]. The KEAP1-Nrf2 PPI inhibitors have potent antioxidant activity by activating Nrf2 pathways and inhibit 17β-estradiol (E2)-induced gene and protein expression in estrogen receptor (ER)-positive human breast cancer cells MCF-7. KEAP1-Nrf2 PPI inhibitors caused significant activation of Nrf2 target genes. E2 decreased the mRNA and protein level of the Nrf2 target gene NQO1, and the KEAP1-Nrf2 PPI inhibitors reversed this effect [215].

### 4.2. Electrophilic Nrf2 Modulators

Electrophilic Nrf2 modulators operate through a conformational cycling mechanism rather than disrupting the KEAP1-Neh2 interaction [181]. These compounds modify reactive cysteine residues within KEAP1, thereby impairing its E3 ubiquitin ligase activity and preventing Nrf2 degradation. The intervening region (IVR), or linker domain, located between the BTB and DGR domains, is enriched with oxidation-sensitive cysteine residues [183,216]. Therefore, therapeutic strategies for AD have focused on targeting Cys151 within the BTB domain using electrophilic agents, such as isothiocyanate sulforaphane and synthetic CDDO derivatives. Sulforaphane is a naturally occurring Nrf2 activator [187,217,218,219], originally identified as a potent inducer of NQO1 in Brassicaceae plants, which activates Nrf2 via KEAP1 Cys151 modification, penetrates the BBB, and exhibits neuroprotective effects in multiple preclinical models. CDDO and its derivatives [178,216,220], including CDDO-Me and CDDO-Im, induce Nrf2 target genes, such as NQO1 and HO-1. CDDO, also known as bardoxolone, is a strong Nrf2 inducer derived from oleanane triterpenoids with known antitumor and anti-inflammatory properties, although its therapeutic utility is limited by insufficient reagent specificity [216].

As electrophilic modulators, chromone-containing molecules may exemplify the mechanistic versatility of Nrf2 activation [221]. Their α, β-unsaturated ketone moiety enables Michael addition to cysteine residues on KEAP1, disturbing the KEAP1-Nrf2-CUL3 complex and inducing ARE-driven cytoprotective gene expression. In addition to this canonical mechanism, chromones can activate Nrf2 through kinase-mediated phosphorylation, KEAP1 autophagic degradation, and crosstalk with other stress-response pathways. These properties have been exploited in the design of multi-target-directed ligands (MTDLs) combining chromone scaffolds with donepezil-derived pharmacophores to simultaneously confer antioxidant activity and acetylcholinesterase inhibition [222,223]. Accordingly, MTDLs as hybrid molecules integrating dimethyl fumarate, tranilast, or dithiocarbamates exhibit strong AChE inhibition, robust Nrf2 induction, BBB permeability, and cognitive improvement in preclinical models. Collectively, these findings support the MTDL paradigm as a rational strategy for AD, offering synergistic symptomatic relief and disease-modifying neuroprotection by targeting multiple pathological hallmarks within a single chemical entity, as AD remains a multifactorial disorder involving cholinergic dysfunction [224,225], oxidative stress, amyloid pathology, tau aggregation, and chronic inflammation [147].

Beyond KEAP1, indirect strategies extend to the repression of negative regulators, such as BACH1 [127]. Although Nrf2 is often stabilized during chronic stress, its transcriptional output in neurodegeneration can be blunted by BACH1 induction. Genetic and experimental evidence, including observations in aging cells, has identified BACH1 as a key brake on Nrf2-driven gene expression. Therefore, dual-targeting strategies that combine Nrf2 activation with BACH1 inhibition offer enhanced therapeutic potential. In particular, dual modulation of Nrf2 activation and BACH1 inhibition has emerged as a promising approach to amplify HMOX1 expression and antioxidant defenses, exemplified by an O-methyl-p-cannabidiol quinone derivative that acts as both a BACH1 inhibitor and an Nrf2 activator. The benzimidazole derivative HPPE exemplifies this approach by inhibiting BACH1 while activating Nrf2 without nonspecific thiol reactivity, leading to reduced oxidative stress, neuroinflammation, and glial activation [187,193]. New dual functional BACH1 inhibitors/Nrf2 activators, such as xanthohumol and alantolactone were identified as promising molecules for the development of anti-metastatic therapies and potential treatments for diseases driven by oxidative stress and inflammation [226].

Another strategy uses microRNAs, which represent an important epigenetic mechanism regulating Nrf2 signaling, as several miRNAs, including miR-7 [189] and miR-592 [192] directly target related regulators such as AD-associated genes, ARE-associated genes, or KEAP1, thereby releasing Nrf2 from KEAP1-mediated inhibition and activating the KEAP1/Nrf2/ARE pathway [188,190]. Growing interest in RNA-based therapeutics has highlighted their potential in neurodegenerative diseases, as they enable precise modulation of disease-relevant transcripts, rapid development, and personalized targeting of pathways that are otherwise difficult to address with conventional drugs [187,191,227].

### 4.3. Combination Approaches with Other Pathways

Given the heterogeneity of AD, therapeutic strategies increasingly emphasize pathway convergence, particularly those regulating oxidative stress and neuroinflammation, and mitochondrial dysfunction [1]. KEAP1-degrading PROTACs sustain Nrf2 activation but require caution due to KEAP1’s extensive interactome [183]. Complementary approaches include KEAP1-recruiting tau PROTACs, which employ an Nrf2-derived peptide to achieve effective tau degradation in neuronal models, although peptide-based limitations constrain in vivo translation [196,213,228].

The convergence of Nrf2 signaling with tau and amyloid pathologies further supports other multi-target approaches. Nrf2 function depends on its interactome, which includes KEAP1, β-TrCP, GSK3β Mafs, and many regulatory mediators [184]. GSK-3 is a central neuronal kinase responsible for regulating tau phosphorylation, a modification essential for tau function, but pathogenic when it is dysregulated. GSK3β overactivation drives tau hyperphosphorylation, making dual GSK3β inhibition and Nrf2 activation a compelling strategy [184,186,229,230]. Dimethyl fumarate exemplifies this concept, activating Nrf2 via both KEAP1-dependent and PI3K/AKT/GSK3β-mediated mechanisms while reducing tau phosphorylation and neuroinflammation in preclinical models. Building on this paradigm, novel 2,4-dihydropyrano[2,3-c]pyrazoles have emerged as the first compounds to combine GSK3β inhibition with Nrf2 induction in a single scaffold, promoting tau clearance via autophagy and improving neuronal survival without detectable toxicity [230,231].

In addition, small-molecule Nrf2 activators acting independently of KEAP1, including methazolamide, melatonin, and Trolox, are known to prevent cytochrome c release and protect mitochondrial function. These small-molecule Nrf2 activators counter Aβ-induced mitochondrial dysfunction by activating Nrf2 through the PI3K/Akt pathway and inhibiting GSK3β-mediated Nrf2 degradation [231].

In AD, p62/SQSTM1 is associated with neurofibrillary tangles composed of hyperphosphorylated tau and ubiquitin, and its reduced expression in the frontal cortex disrupts Nrf2 signaling, increases oxidative stress, and compromises neuronal survival. Phosphorylation of p62/SQSTM1 enhances its affinity for KEAP1, linking autophagy to Nrf2 activation and facilitating the degradation of ubiquitinated protein aggregates and damaged mitochondria, a process critical for preventing ROS accumulation and cytotoxic aggregation [147,232]. Experimental studies in mice and human AD brains further demonstrated that loss or dysfunction of p62/SQSTM1 impairs mitophagy, promotes tau pathology, and contributes to oxidative damage, highlighting the p62/SQSTM1-Nrf2-autophagy axis as a key protective mechanism under physiological stress and a potential therapeutic target in neurodegeneration [197].

Soluble Aβ oligomers (AβOs), now established as central mediators of AD pathogenesis, induce oxidative stress, mitochondrial dysfunction, synaptotoxicity, and neuroinflammation [233]. Current therapeutic strategies that neutralize AβOs or inhibit their formation remain inadequate, emphasizing the need for interventions that directly address AβO-induced toxicity. Small molecules possessing phenolic or flavonic nature that activate Nrf2 and/or PPARγ demonstrate significant efficacy in reducing AβO toxicity by alleviating oxidative stress, suppressing neuroinflammation, and preserving synaptic function, supporting the rationale for Aβ oligomer toxicity-reducing therapy (ATR-T) as an adjunctive treatment for AD [194].

Parallel multi-target strategies integrate Nrf2 activation with the inhibition of amyloid-β production through BACE-1 [234]. Beta-site APP-cleaving enzyme 1 (BACE1) is a brain-expressed aspartyl protease required for amyloid-β (Aβ) production, whose aggregation into insoluble plaques initiates toxicity in AD, making BACE1 a well-validated therapeutic target and the basis for developing potent BACE1 inhibitors. Genetic and experimental studies have shown that reducing BACE1 activity lowers Aβ levels, decreases plaque formation, and protects cognitive function. Fragment-based drug discovery and structure-guided optimization led to the identification of verubecestat, a non-peptidic, high-affinity BACE1 inhibitor that engages the catalytic dyad and occupies multiple hydrophobic subsites. Verubecestat exhibits favorable oral bioavailability, brain penetration, and physicochemical properties, becoming the first BACE1 inhibitor to advance Phase III clinical trials and a critical test of both BACE1 inhibition and the amyloid hypothesis in AD (ClinicalTrials.gov ID: NCT02477800 and NCT02484547) [133,235].

Neuroinflammatory processes also intersect with Nrf2 signaling via the endocannabinoid system [197]. Activation of microglial CB2 receptors suppresses NF-κB-driven inflammation, an effect dependent on Nrf2-mediated HO-1 induction [147]. CB2 agonists promote a shift toward a neuroprotective microglial phenotype, whereas evidence of an ARE within the CNR2 promoter suggests reciprocal regulation by Nrf2. The nutraceutical characteristics of Mediterranean diets, including β-caryophyllene, polyphenols, and antioxidant vitamins, further amplify this axis, enhancing mitochondrial biogenesis and reducing neuroinflammation [197].

## 5. Plant Bioactive Molecules as Nrf2 Activators in AD

**Figure 8:**
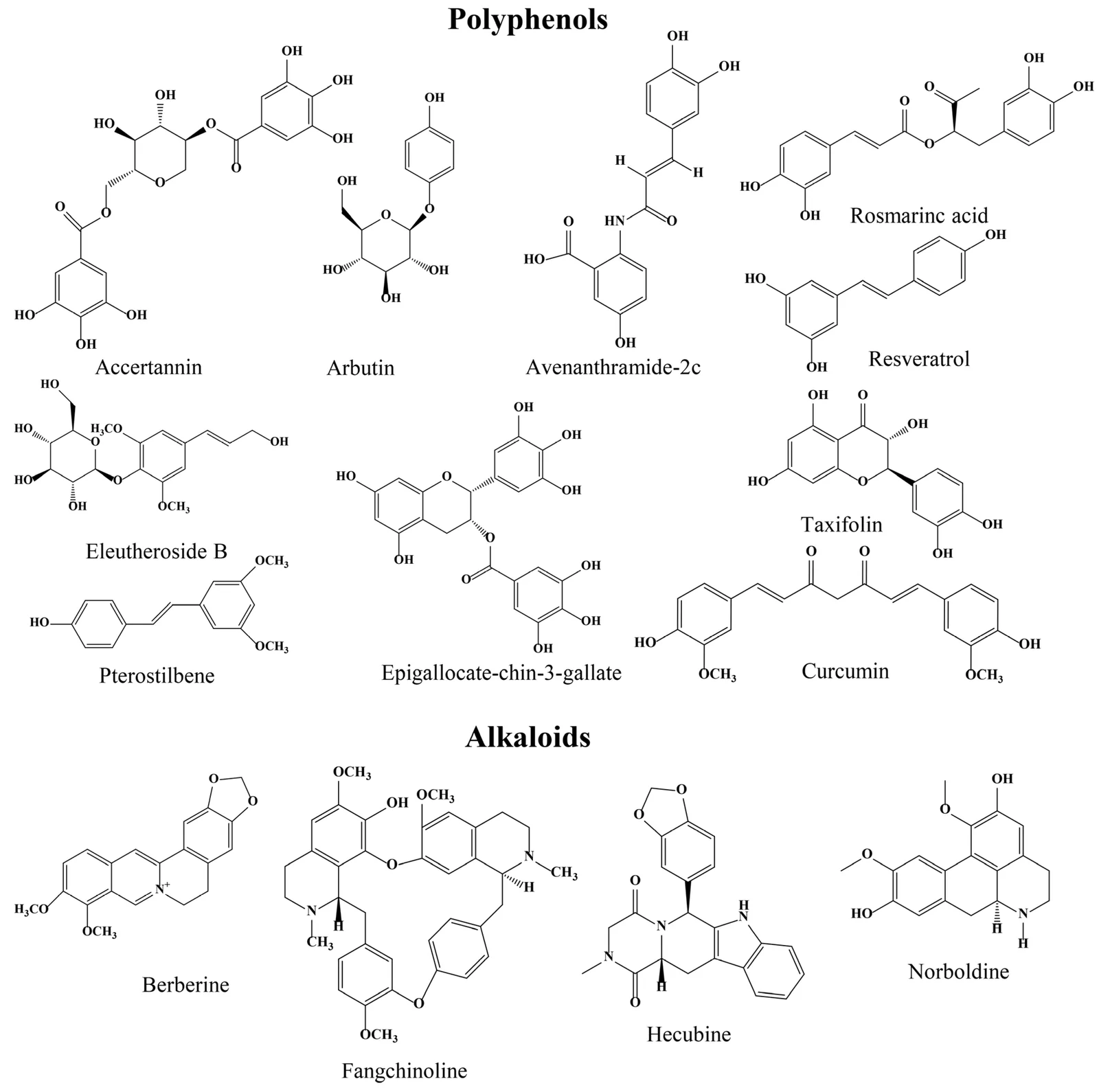
Chemical structures of selected plant-derived molecules discussed in the article.

Since times immemorial, plants and plant-based products have been used as primary source in medicine and nowadays. They are still preferred over the synthetic drugs due to their beneficial effect towards numerous human diseases, fewer side effects and their cost-effectiveness [236], as well as, their inseparable companion in the diet as food or dietary supplements [237]. Undoubtedly, plants and plant-derived constituents have irreplaceable role in drug discovery and are invaluable source of drug candidates [238]. Plenty epidemiological and experimental studies have demonstrated the potential of phytochemicals as therapeutic agent against oxidative stress, which is a major factor in the mechanism underlying many human diseases [239]. Consequently, the amelioration of inflammation and oxidative stress via Nrf2 signaling activation has become an attractive target in the treatment of AD [3]. Nevertheless, the pharmacotherapy of AD has long relied mainly on symptomatic treatments, including the acetylcholinesterase inhibitors tacrine, rivastigmine, galantamine and donepezil, and the N-methyl-D-aspartate (NMDA) receptor antagonist memantine; these agents modulate neurotransmission but do not alter the underlying disease process. In spite of that, these drugs only provide temporary improvement in cognitive functions and cannot end or reverse the underlying progress of AD. Furthermore, the listed drugs have a wide range of side effects including vomiting, diarrhea, nausea, dizziness and headaches, and to date none of them has shown significant effect in AD clinical trials [3]. More recently, the therapeutic landscape has expanded with amyloid β-directed disease-modifying therapies. Lecanemab, a monoclonal antibody that received traditional FDA approval in 2023, targets aggregated soluble and insoluble forms of Aβ, while donanemab, approved by the FDA in 2024, binds insoluble N-truncated pyroglutamate Aβ; both are intended for early symptomatic AD, including mild cognitive impairment or mild dementia due to AD with confirmed amyloid pathology [240,241,242,243,244]. Therefore, considering Nrf2’s ability to interfere with both Aβ and p-tau and the fact that the majority of AD patients are elderly people, Nrf2 activation is an attractive target for treatment of neurodegenerative diseases, including AD [3]. Moreover, different phytochemicals, including polyphenols (flavonoids, phenolic acids, stilbenes, and lignans), terpenoids, alkaloids, organosulfur compounds, etc., have been studied as potential activators of Nrf2 [245]. The chemical structures of selected molecules are presented in Figure 8.

Selected examples of plant species and plant-derived molecules able to modulate Nrf2 pathway in a variety of experimental in vitro and in vivo models are presented in Table 2.

### 5.1. Polyphenols

Polyphenols activate Nrf2 through various mechanisms, including: (1) modification of KEAP1, which leads to release and nuclear translocation of Nrf2, its binding to ARE and activation of the expression of the genes involved in antioxidant defense; (2) activation of kinase signaling pathways (PKC, MAPKs, PI3K/Akt), which phosphorylate Nrf2 or its negative regulator contributing to stabilization and nuclear translocation of Nrf2; and (3) epigenetic modifications through modulation of the activity of enzymes involved in DNA methylation and histone modification [5].

Emerging evidence reveals that the Mediterranean diet (MD), which is particularly rich in polyphenols from extra virgin olive oil, is associated with improved cognitive function and reduced risk of mild cognitive impairment. Polyphenols can activate Nrf2, enhancing endogenous antioxidant defenses. Several polyphenols, such as apigenin, luteolin, quercetin, puerarin, hesperidin and caffeic acid were found to activate Nrf2. Sixteen human target genes, namely *NFE2L2*, *SLC7A11*, *GSK3B*, *NQO1*, *AKR1B1*, *GLS*, *EGFR*, *RELA*, *PRKCB*, *GCLC*, *CSNK1A1*, *MAPK11*, *MAPK14*, *CSNK2A1*, *PRKCA*, and *JUN*, exhibited differential expression in AD [279]. Rosmarinic acid is a promising candidate for neuroprotective treatment of AD, studied in vitro in the neuronal cell model PC12. Rosmarinic acid attenuated Aβ-induced cellular ROS generation, and lipid hydroperoxides (LPO). In vivo, acteoside treatment significantly preserved dopaminergic neurons and curbed ferroptosis in them while alleviating cognitive and behavioral deficits [278]. The effect of arbutin (hydroquinone-β-D glucopyranoside), a dietary soluble glycosylated phenol was studied in the local demyelination model in the rat optic chiasm. Daily injection of arbutin (50 mg/kg, i.p) over 2 weeks decreased inflammatory cytokines (IL-1B, IL-17, TNF-α) and iNOS mRNA expression level. On the other hand, the expression of the anti-inflammatory cytokine IL-10 and the antioxidant mediators Nrf2 and HO-1 were enhanced by arbutin treatment [247]. In chickens with chronic heat-stress-induced liver oxidative damage, administration of resveratrol (400 mg/kg) enhanced the activities of liver antioxidant enzymes, resulting in higher GPX and SOD levels than those in the heat-stressed broilers, and decreased MDA levels. Furthermore, resveratrol activated the gene and protein levels of Nrf2 and HO-1; thus, enhancing NQO1 and SOD1 gene levels, and decreasing protein levels of HSP70, p62, and KEAP1 [260]. Raisin extract (100 and 200 µg/mL) rich in polyphenols (e.g., isoquercitrin and 6-hydroxykaempferol-7-O-glucoside) alleviated the oxidative stress in H_2_O_2_-induced HepG2 cells and D-gal-induced aging mice. The observed effect was due to an increase in the cellular levels of the antioxidant enzymes SOD, CAT and GSH and a decrease in MDA, ROS and advanced glycosylation end-product (AGEs) levels. Mechanistically, the antioxidant and anti-aging was explained via the regulation of Sirt1/Nrf2 signaling [280].

Resveratrol (the active molecule extracted from grapes, berries, wine, etc.) has a multitude of functions, such as anti-inflammatory, anti-oxidation, anti-cancer and cardiovascular protection. In a mouse model of thoracic blast exposure-induced brain injury, the administration of 25 and 50 mg/kg resveratrol ameliorated cognitive dysfunction and anxious behavior. Additionally, it was also found that the observed effects were due to modulation of Nrf2/KEAP1 and NF-κB signaling (amelioration of the induced KEAP1 and NF-κB) and a decrease in Nrf2 expression in the brain. In addition, the levels of several inflammatory-related factors, such as IL-1β, IL-4 and high-mobility group box protein 1 (HMGB1) were significantly reduced, while the levels of the anti-inflammatory factor IL-10 were increased [259]. Multifunctional nanocomposites consisting of graphene quantum dots encapsulated within a metal–polyphenol network formed by physiological Co (II)-coordinated epigallocatechin gallate (EGCG) could offer new avenues for developing novel AD inhibitors. The nanocomposites effectively modulate the AD microenvironment by inhibiting Aβ42 amyloidosis, attenuating oxidative stress, and regulating microglial activity. The nanocomposite formulation alleviated neuroinflammation through scavenging intracellular ROS and regulating the transformation of microglia from M1 phenotype to M2 phenotype. The underlying molecular mechanism was found to be the upregulation of Nrf2 and NF-κB signaling inhibition [281]. Oligonol (a low-molecular-weight polyphenol derived from lychee fruit) prevented dimethylnitrosamine (DMN)-induced liver fibrosis in male Sprague–Dawley rats. It was demonstrated that oligonol showed antioxidative, hepatoprotective, and anti-fibrotic effects via JNK/NF-κB and PI3K/Akt/Nrf2 signaling pathways in DMN-intoxicated rats. The compound prevented the elevations of TNF-α, IL-1β, IL-6, COX-2, and iNOS expressions at the mRNA level. NF-κB activation and JNK phosphorylation in DMN-treated rats were ablated by oligonol. The enhanced production of hepatic MDA and ROS were reduced. The nuclear translocation of Nrf2 was enhanced, while PI3K and phosphorylated Akt levels were increased. The fibrosis markers, such as α-SMA, TGF-β1, and type I collagen, were reduced in rats treated with oligonol [257].

Taxifolin may induce Nrf2 expression and its downstream target genes in mouse skin JB6 P+ cells via an epigenetic pathway by CpG demethylation in the Nrf2 gene promoter region. The compound revealed its skin cancer chemoprotection by inducing Nrf2/ARE pathway, upregulation of mRNA and protein levels of its downstream genes Nrf2, HO-1, NQO1. Taxifolin reduced the methylation level of the first 15 CpGs sites in the Nrf2 promoter [263]. The epigenetic modification of sulforaphane increased the antioxidative and anti-inflammatory capacity in AD model of mouse neuroblastoma N2a cells. At concentrations of 1.25 and 2.5 µM sulforaphane decreased the levels of Aβ_1–40_, the intracellular Aβ_1–42_, ROS, MDA and increased SOD activity. The proposed mechanism of activity was that sulforaphane upregulated the expression of Nrf2 and promoted its nuclear translocation by decreasing DNA demethylation levels of the Nrf2 promoter [282]. Pterostilbene reversed epigenetic silencing of Nrf2 and enhances antioxidant response in endothelial cells in hyperglycemic microenvironment. Pterostilbene upregulated the epigenetic markers, such as HDACs class I-IV and DNMTs 1/3A and 3B. Furthermore, the compound counteracted the hypermethylation of the CpG islands in the Nrf2 promoter in the cells [258].

### 5.2. Alkaloids

Berberine is one of the natural alkaloids that can penetrate the BBB and may be effective in AD underlying pathology. Berberine successfully mitigated the cognitive disorders in triple transgenic AD (3 × Tg-AD) mice. A reduction in Aβ plaque, hyperphosphorylated tau protein, neuronal loss, and ferroptosis were observed. The attenuated apoptosis was due to decreased MDA and ROS, while enhanced SOD, GSH, GPX and SLC7A11 was due to upregulation of Nrf2 transcription levels [269]. Berberine ameliorated chronic unpredictable mild-stress-induced depression-like behavior in rats. Treatment with the compound (50 and 100 mg/kg) upregulated the expression of KEAP1/Nrf2 antioxidant signaling pathway and its downstream neuroprotective factors, including upregulation of SOD and downregulation of MDA [268]. The protective properties of berberine in neurodegenerative and neuropsychiatric disorders have been demonstrated in an immortalized mouse neural progenitor C17.2 cells. Berberine was able to protect from oxidative damage through lowering ROS levels, upregulation of Nrf1/2, NQO1 and HO-1. It also downregulated the apoptotic factors caspase-3 and Bax and increased the expression of Bcl-2. Furthermore, berberine increased C17.2 cell viability via upregulating extracellular-signal-related kinase (ERK) and phosphor-extracellular signal-regulated kinase (pERK) expression [270]. Alkaloids containing extract from *Oxalis corniculata* Linn had potential anti-AD activity in rats. The antioxidant defense was restored via Nrf2/HO-1 hub activation and hampered neuroinflammation via TLR4/NF-κβ/NLRP3 downregulation [274]. Hecubine (a natural small molecule aspidosperma-type alkaloid) has potential as anti-inflammatory and neuroprotective agent. Hecubine upregulated Nrf2 expression levels while downregulating TLR4 signaling expression levels, as well as suppressing the proinflammatory mediators NO, TNF-α, IL-6, and IL-1β [272]. Fangchinoline (a bisbenzylisoquinoline alkaloid) significantly ameliorated cognitive impairment in an Aβ_1–42_-induced mouse model of AD via the induction of autophagy and the inhibition of oxidative stress. This was due to increased levels of GSH-PX, SOD and activation of Nrf2 and HO-1 [271]. Norboldine (an alkaloid extracted from *Lindera aggregata* (Sims) Kosterm) was studied for improving the cognitive impairment and pathological features of 3 × Tg mice as a model of AD. Norboldine treatment improved the learning and memory capacity of 3 × Tg mice and alleviated AD pathology such as Aβ deposition. The alkaloid restored the abnormalities of mitochondrial membrane potential and significantly reduced the production of intracellular ROS and neuronal cell apoptosis. It was considered that, mechanistically, norboldine might exert antioxidant and anti-apoptotic activity through the AMPK/GSK3β/Nrf2 signaling pathway [273].

### 5.3. Organosulfur Compounds

Sulforaphane is an isothiocyanate naturally found in cruciferous vegetables such as broccoli, Brussels sprouts, cabbage, and kale. It is well known for its antioxidative and anti-inflammatory effects and potent activator of the Nrf2 pathway. Sulforaphane has attracted attention for its health benefits, particularly its role in brain health and the potential prevention of dementia and neurodegeneration. Sulforaphane effectively reduced Aβ and toxic p-tau accumulation in vascular cognitive impairment. The protective mechanism involved induction of Nrf2 and HO-1 expression, activation of Akt/GSK3β pathway and modulation of APP expression levels. GSK3β is considered the major kinase responsible for the phosphorylation of tau. Its activities are inhibited by phosphorylation on amino acid residues Ser9 and Ser389 by p-Akt (the phosphorylation-activated form of Akt) and other kinases. Sulforaphane inhibits short-term neuronal and white matter injuries, as well as long-term Aβ and p-tau accumulation caused by cerebral vascular lesions, making it a promising therapeutic compound for treating vascular cognitive impairment and AD [276]. Sulforaphane-rich broccoli sprout extract of 100 mg/kg or 200 mg/kg administrated once daily for four weeks significantly improved scopolamine-induced deficits in long-term memory in an AD C57BL/6J mice model. The low dose of sulforaphane preserved striatal choline acetyl transferase expression and reduced systemic TNF-α and hippocampal COX-2 levels, indicating its anti-inflammatory and cholinergic protective effects [275]. In an immortalized cortical neuronal model (CN 1.4 cells), sulforaphane significantly prevented mitochondrial abnormalities, and reduced ROS levels. The prevention of mitochondrial dysfunction induced by pathological forms of tau in this AD model was mechanistically explained by increased Nrf2 expression, as well as the activation of the Nrf2 pathway-related genes, such as NQO1, HO-1, GCS, GR1, and TRxR1 [277].

## 6. *Caenorhabditis elegans* as a Model for Studying Nrf2 Activation in AD

### 6.1. Advantages of C. elegans in AD Research

The continuous progress in healthcare systems, technology and education has increased our quality of life and longevity. However, increased longevity is being attended by a rise in incidences of age-related diseases, including AD, characterized by three core features: progressive systemic functional decline, impaired stress-responsive homeostatic regulation, and structural degradation of biological systems [14,283]. Despite extensive scientific investigations, the molecular mechanisms that underline the pathology of AD remain incompletely elucidated [283]. Numerous biomodels, such as murine models, cell lines and *C. elegans*, have been used to answer these questions. Mammalian in vivo models have several limitations, such as results reproducibility, high experimental costs and time duration. Along with that, the complexities of the human brain cannot be simulated completely with mammalian neuronal cell cultures in vitro, encouraging scientists to search for alternative models, such as *C. elegans*, for screening of anti-Alzheimer’s drugs [284].

*Caenorhabditis elegans* (phylum Nematoda, family Rhabditidae) is a non-parasitic, free-living round worm mostly found in temperate soils in all regions of the world. It was first introduced by Sydney Brenner in the 1960s (as a model organism to study development and neurobiology), and together with his postdoctoral fellows John E. Sulston and H. Robert Horvitz (using *C. elegans* to elucidate the fundamental regulation of cell growth and controlled cell death) he was awarded in 2002 the Nobel Prize in Physiology [285]. Since then *C. elegans* has been used as a model system to study a vast array of biological processes, such as apoptosis, gene regulation, cell metabolism and cell fate regulation, embryogenesis, stress signaling, aging, obesity, locomotive activity, and neurodegenerative disorders [4,286]. *Caenorhabditis elegans* seems to be the ideal genetic system compared to other classical animal models. Even techniques such as RNA interference (RNAi) and CRISPR/Cas9 gene editing could be easily applied [287]. It has a small size, the adults reaching 1 mm in length and 80 µm in diameter, and is very easy to cultivate in laboratory conditions either on agar plates or in liquid medium, supplemented with *Escherichia coli* (mainly OP50 strain), and working with it does not require approval by institutional animal care and use committees [288]. At 20 °C, it has a short lifecycle of 3 days from egg to adult (developing through four larval stages, L1–L4) and large progeny production of approximately 300 offspring from a single adult self-fertilizing hermaphrodite, thus allowing quick evaluation of age-related traits and lifespan extension, which are essential in aging studies [285]. The nematode has a simple and fully annotated genome (97 Mb size), which along with the simple structure of its nervous system, consisting of only 302 neurons in an adult nematode, makes it valuable for screening drugs against age-associated neurodegeneration, and genetic manipulations can also be carried out easily [289]. *Caenorhabditis elegans* has two sexual forms: self-fertilizing hermaphrodites (XX) and males (XO), being about 0.2% of the total population due to rare meiotic non-disjunction of the X-chromosome. The mature adult hermaphrodite nematodes have 959 somatic cells, while males have 1031. These somatic cells have a high degree of differentiation, allowing many mammals’ physiological processes to have their homolog in *C. elegans*. The nervous system consists of 302 neurons and 56 glial cells in hermaphrodites, and 381 neurons and 92 glial cells in males, which corresponds to almost a third of the total number of cells in the organism. Most neuronal somas are located in the head of the worms, clustered in the ganglia, and compared with other organisms, there is a great variety of neuron types (118 for hermaphrodites) that allows *C. elegans* to perform complex behaviors mostly related to sensory processes [4]. Between 60% and 80% of human genes are orthologous to the *C. elegans* genome, and between 38% and 42% of genes known to be associated with human diseases have orthologs in the *C. elegans* genome [290,291]. Finally, *C. elegans* is transparent at all stages of its life cycle, allowing easy visualization, observation and monitoring of physiological and cellular processes, including neuronal structures and morphologies. These can be made easily traceable by expressing green fluorescent protein (GFP) in a neuron of choice, and the cellular localization of a protein can be visualized by tagging the protein with GFP [291,292]. Many key cellular metabolic and signaling pathways are conserved between *C. elegans* and mammals, such as insulin signaling pathways and essential neurodegenerative disease pathways, such as those controlled by APP, presenilins, and genes implicated in both early-onset (EOAD) and late-onset (LOAD) AD [293]. The molecular mechanisms involved in Aβ processing, tau phosphorylation, and proteostasis are highly conserved functionally, making *C. elegans* a valuable model for investigating AD at the molecular level, since Aβ expression influences similar pathways in worms, mice, and humans [294]. Some of the major advantages and limitations of the most frequently used AD models, including *C. elegans* are presented in Table 3.

### 6.2. Limitations and Strategies to Overcome Them

Despite its advantages, *C. elegans* has significant limitations for modeling human AD [294]. The organism lacks a hippocampus and cerebral cortex, the brain regions most vulnerable to AD pathology. Its synaptic connectivity differs substantially from mammals, and the absence of an adaptive immune system prevents modeling of neuroinflammatory components involving T cells and B cells. Additionally, *C. elegans* lacks several genes critical for AD pathogenesis, including the *APOE* gene and the *BACE1*, making endogenous production of the pathogenic Aβ_1–42_ peptide impossible [294]. Other major limitation is to estimate the actual concentration of drug ingested or absorbed by the nematode from the supplied concentration of the drug in the nematode culture media, which has been to some extend overcame by quantifying the actual concentration of the internalised drug substance with the help of liquid chromatography–mass spectrometry (LC-MS) analysis of the lysate of the nematode [297].

Furthermore, while *C. elegans* lacks adaptive immunity, it possesses a sophisticated innate immune system regulated by p38 MAPK and Nrf2/SKN-1 pathways, permitting investigation of innate immune-mediated neuroinflammation. Recent studies have also shown that several AD-associated genes—including *APOE* (heterologous expression models), insulin signaling components, and cholesterol metabolism enzymes—have functional orthologues or analogues in the worm, partially preserving relevant genetic networks [298].

To circumvent these limitations, researchers have engineered transgenic *C. elegans* strains expressing human AD-associated proteins using various promoters and inducible systems [293,294]. These transgenic models reproduce key hallmarks of AD—Aβ accumulation, tau hyperphosphorylation, neurodegeneration, and behavioral deficits—without requiring endogenous production of these pathogenic proteins [293,294]. Temperature-inducible systems (such as the myo-3 promoter with temperature shifts in CL4176) allow investigators to control the temporal onset of neurodegeneration, mimicking age-related disease progression. These strategies have transformed *C. elegans* into a tractable system for dissecting AD mechanisms at the molecular level [294]. In *C. elegans*, transgenes are present as extrachromosomal arrays and are not integrated into the genome as they are in other systems; a few copies to several hundred copies of the transgene are present in the arrays, so the level of overexpression can be much higher than what is found in vivo. Fortunately, methods for single copy insertions have now been developed [299].

### 6.3. High-Throughput Screening Capabilities

Beyond classical genetics, *C. elegans* is highly effective in high-throughput screening (HTS) applications [300]. Its compatibility with multiwell plate formats, automated liquid handling, and imaging technologies allows for rapid screening of compound libraries [293,300]. The Lifespan Machine, which uses simple flatbed scanners to monitor thousands of worms simultaneously, has made aging research more accessible [293]. Additionally, optogenetics provides precise control over neuronal activity, microfluidic devices like NeuroChip facilitate electrophysiological recordings, and SAM (small molecule accumulation model) predictions help select compounds based on bioavailability [293]. These innovations establish *C. elegans* as a promising platform for translational drug discovery [293].

The first large-scale drug screening using *C. elegans* was done on agar plates in 2006. Among 14,100 small molecules, 308 bioactive compounds were identified by inducing a variety of phenotypes. Among them nemadipine-A induced marked defects in morphology and egg-laying, demonstrating that worms and vertebrates share the orthologous protein target [301]. The use of liquid medium facilitated the workflow to perform screening of 6000 synthetic and 1136 natural extracts for potential antimicrobial activity using an *Enterococcus faecalis* infection model in *C. elegans*. Of them, 16 compounds and 9 extracts promoted nematode survival [302]. Again in liquid medium, the HTS of 88,000 compounds revealed that 48 of them extended lifespan in adult worms by 20–60% [302,303]. The first automated HTS has been performed on a transgenic *C. elegans* expressing the mutant form of human α1-antitrypsin fused with GFP accumulate misfolded protein within the endoplasmic reticulum, similar to that seen in the liver of patients with α1-antitrypsin deficiency (ATD). Among 1280 compounds, 33 hit compounds significantly reduced the intracellular accumulation of misfolded protein aggregates, and fluphenazine in particular was subsequently shown to be efficacious in reducing protein aggregates in mammalian cell line and mouse models of ATD [304]. The AD transgenic model strain (GRU102) that expresses a pathogenic human amyloid beta peptide (Aβ_1–42_) specifically in neurons has been used to identify metformin, lithium and curcumin as potential “hits”. The ability to rescue the swim-exhaustion phenotype correlates well with lifespan and healthspan improvements in GRU102 [305].

## 7. The Nrf2/SKN-1 Pathway: Molecular Architecture and Regulation

### 7.1. Basic Characteristics and Functional Conservation

**Figure 9:**
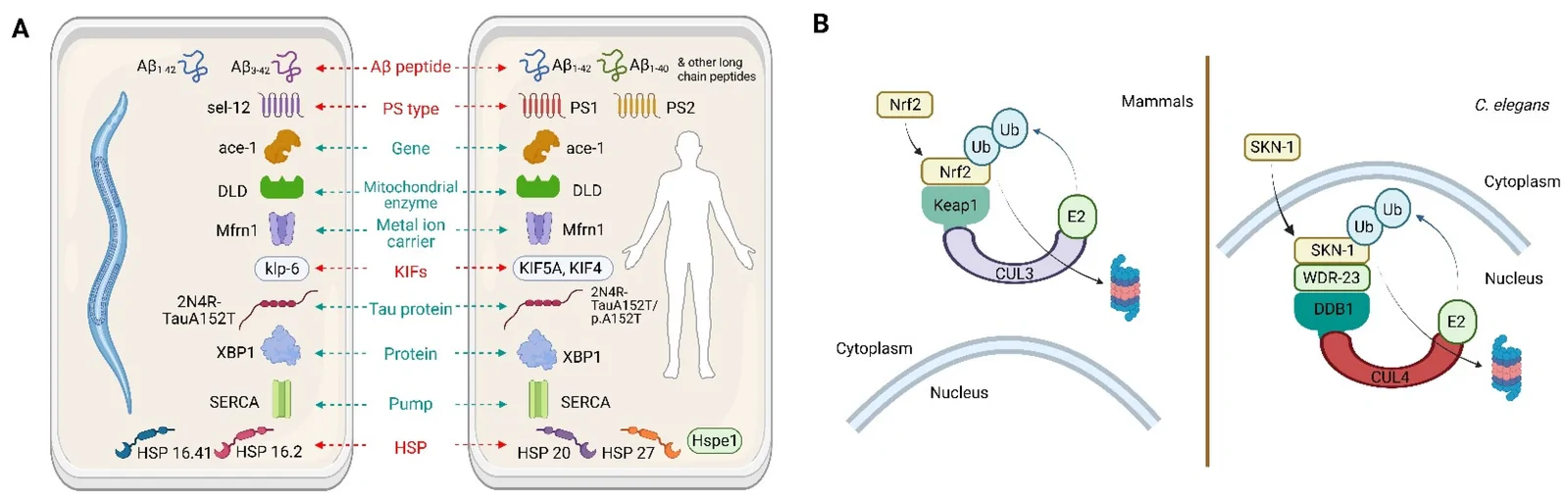
(**A**) *Caenorhabditis elegans* and human protein/gene expression related to AD. All similarities are marked with dark cyan colour, while all differences are in red. Differences: in *C. elegans* the Aβ peptides Aβ_1–42_ and Aβ_3–42_ and sel-1 presenilin are secreted, whereas the Aβ peptides Aβ_1–40_, Aβ_1–42_, presenilin 1 and presenilin 2 are secreted in humans; the kinesin family protein (KIF) in *C. elegans* is klp-6, KIFs, while in humans it is KIF5A or KIF4; the heat shock proteins (HSPs) studied in *C. elegans* are HSP16.41, HSP 16.2, whereas the HSPs studied in humans are HSP20, HSP27 and Hspe 1. Similarities: the ace-1gene, the mitochondrial enzyme dihydrolipoamide dehydrogenase (DLD) and the mitochondrial metal ion carrier mitoferin-1 (Mfrn1) are similar in *C. elegans* and humans; the tau protein 2N4R-Tau A152T secreted in *C. elegans*, the unfolded protein response in *C. elegans* mediated by X box binding protein (XBP 1) and the serco(endo) plasmic reticulum Ca-ATPase pump (SERCA) are similar to humans. (**B**) The regulation of Nrf2 in mammals and SKN-1 in *C. elegans* by distinct ubiquitin ligases. CUL3 and CUL4 are distinct ubiquitin ligases. WDR-23 and KEAP1 are distinct proteins functioning in different cellular compartments (nucleus and cytosol). Both pathways label their target with ubiquitin for proteasomal degradation.

In *C. elegans* SKN-1 is the ortholog of mammalian Nrf/CNC proteins. SKN-1 has diverged considerably from mammalian Nrf/CNC proteins with respect to its mode of DNA binding. However, the degree of functional conservation between SKN-1 and these proteins suggests that *C. elegans* SKN-1 may be a powerful model for investigating the regulation of Nrf2 and other Nrf/CNC proteins, and their influence on the development and functions of normal tissues in vivo. Nrf/CNC proteins are ZIP transcription factors that require their Maf dimerization partner to bind DNA stably, but SKN-1 lacks the ZIP dimerization module. The SKN-1 DNA binding region, along with an additional peptide motif with which SKN-1 recognizes bases in the DNA minor groove, forms a relatively stable helical structure with the CNC domain. Although the SKN-1 recognition preference has diverged from the ARE that is recognized by mammalian Nrf/CNC-Maf dimers, SKN-1 directly regulates many of the very same genes that are known to be Nrf/CNC targets. While mammalian Nrf2 usually binds DNA as a heterodimer with CNC-Maf, *C. elegans* SKN-1 has less strict DNA-binding preferences and can operate as a monomer or a homodimer. Beyond their DNA-binding region, the homology between SKN-1 and Nrf/CNC proteins is limited except for a highly conserved 14-residue motif called DIDLID, which is a unique signature of the Nrf/CNC protein family. This conservation suggests a critical function, and in SKN-1 the DIDLID element is important for transcription activation and interaction with the protein WDR-23, which targets SKN-1 for proteasomal degradation. Four major predicted isoforms are encoded by the *skn-1* gene, three of which have been shown to be expressed in vivo (SKN-1a-c) [306,307]. The differences and similarities in protein gene expression associated with AD in *C. elegans* and humans are illustrated in Figure 9A, while Figure 9B indicates the distinct features in Nrf2 and SKN-1 regulation in *C. elegans* and humans.

### 7.2. Post-Translational Regulation Mechanisms of SKN-1

Recent discoveries have revealed that SKN-1 function is exquisitely regulated by post-translational modifications (PTMs) that modulate its localization, stability, and transcriptional activity. Understanding these regulatory mechanisms is essential for comprehending how cells fine-tune the antioxidant response.

O-GlcNAcylation and nuclear translocation: SKN-1 undergoes O-GlcNAcylation at Ser470 and Thr493, catalyzed by the O-GlcNAc transferase (OGT-1). This modification increases under oxidative stress conditions and promotes nuclear translocation of SKN-1 by preventing inhibitory phosphorylation at Ser483 by GSK-3. The O-GlcNAcylation-mediated release from GSK-3 phosphorylation represents a critical control point, allowing SKN-1 to escape proteasomal degradation and accumulate in the nucleus, where it activates target genes. This PTM is particularly relevant in aging, as O-GlcNAcylation levels are compromised in aged organisms, explaining the age-related decline in SKN-1 stress responsiveness [306].

Phosphorylation and insulin/IGF-1 signaling (IIS): The IIS pathway plays a central role in regulating SKN-1 activity and lifespan [306,308]. Normally, kinases AKT-1, AKT-2, and SGK-1 phosphorylate SKN-1 at various sites, which prevents it from accumulating in the nucleus and instead keeps it in the cytoplasm or targets it for proteasomal degradation [306]. When IIS activity decreases—due to mutations in daf-2 or age-1, or through akt gene knockdown—SKN-1 is dephosphorylated, allowing it to move into the nucleus, where it activates antioxidant and longevity pathways. This process connects nutrient sensing, metabolic health, and stress response, a relationship conserved across species from *C. elegans* to mammals [306].

WDR-23 regulation and isoform-specific effects: The WD-repeat protein WDR-23 serves as a functional analogue of KEAP1, controlling SKN-1 stability and localization through the CUL-4/DDB-1 E3 ubiquitin ligase complex. Remarkably, WDR-23 exists as two distinct isoforms with opposite functions: the cytoplasmic isoform WDR-23A acts as an activator of SKN-1 transcriptional activity, while the nuclear isoform WDR-23B promotes SKN-1 proteasomal degradation. Loss-of-function mutations in *wdr-23* result in constitutive SKN-1 activation, evident from a 40-fold increase in *gst-4* mRNA expression and dramatic lifespan extension (up to 50% in some genetic backgrounds). This isoform-specific regulation suggests potential therapeutic strategies targeting WDR-23 localization to enhance antioxidant response without triggering excessive activation [309].

### 7.3. Tissue-Specific and Cell Non-Autonomous Functions

A groundbreaking discovery in the SKN-1 research is that intestinal SKN-1 influences neuromuscular junction (NMJ) function and neuronal health via cell non-autonomous signaling [309]. This process involves several signaling pathways: the EGL-15 FGFR pathway (mainly in the hypodermis), DAF-2 insulin receptor signaling across various tissues, and sphingosine-1-phosphate (SphP)/SPHK-1 pathways. Intestinal SKN-1 controls neuropeptide release from motor neurons, linking overall metabolic health with neuronal function. This tissue-specific control offers significant insights into how peripheral antioxidant responses can protect the central nervous system, a concept possibly relevant to AD, where systemic metabolic health affects neurodegeneration [309].

Cell non-autonomous SKN-1 signaling involves several intermediate stages. Initially, activating SKN-1 in the intestine increases the expression of specific neuropeptide genes. These neuropeptides are secreted and then bind to receptors on neurons, affecting neuronal function and stress resilience [309]. Furthermore, intestinal SKN-1 controls lipid metabolism and fatty acid oxidation, which subsequently impact mitochondrial function in neurons. This complex communication between tissues underscores the importance of studying SKN-1 within entire organisms rather than in isolated cells [309].

### 7.4. Metabolic Integration: Proline Catabolism and Lipid Metabolism

Recent research has revealed an intriguing connection between SKN-1/Nrf2 and metabolic reprogramming [310]. Specifically, SKN-1 links proline breakdown with lipid metabolism during fasting and oxidative stress [310]. When nutrients are limited, SKN-1 increases the expression of genes for proline dehydrogenase (POX enzymes) and other catabolic enzymes, allowing the breakdown of proline, a non-essential amino acid abundant in cellular proteins and fibrils like Aβ plaques, into α-ketoglutarate, an essential TCA cycle intermediate. At the same time, SKN-1 enhances fatty acid oxidation (FAO) gene expression, supporting mitochondrial β-oxidation of lipids to generate ATP [310].

This metabolic integration is mediated by the transcriptional co-regulator MDT-15 and involves modulation of PGC-1α-related signaling [310]. The coupling of proline catabolism with FAO is particularly interesting in the context of AD, where pathological protein aggregates accumulate and energy metabolism becomes compromised. By promoting both protein catabolism (via proline metabolism) and alternative fuel oxidation (FAO), SKN-1 activation may facilitate clearance of misfolded proteins while sustaining energy production [310].

### 7.5. Crosstalk with Other Stress-Response Pathways

SKN-1 functions not in isolation but in concert with other major longevity and stress-response pathways, creating a complex regulatory network. The transcription factor DAF-16, which is the human FOXO3 orthologue, activates a parallel longevity pathway when IIS activity decreases [306]. Notably, SKN-1 and DAF-16 have both overlapping and unique roles [306,308]. They regulate some common genes, such as certain *gst* genes, but each can independently promote lifespan extension. SKN-1 contributes through enhancing resistance to oxidative stress and metabolic changes, while DAF-16 influences lifespan via Dauer formation, stress response, and metabolic adjustments. During the reproductive phase, SKN-1 and DAF-16 seem to work in parallel; however, their roles converge during Dauer entry and recovery. This suggests that simultaneous targeting of SKN-1 and DAF-16 could lead to additive protective effects against neurodegeneration [306].

Recent evidence shows that SKN-1 surprisingly inhibits autophagy during prolonged oxidative stress. Mechanistically, when oxidative stress continues, SKN-1 binds to the *aak-2* (*C. elegans* AMPK) promoter, reducing its expression. This decrease in AMPK prevents hyperactive autophagy, which can become harmful and cause autophagic cell death. SKN-1 thus plays a dual role: it encourages early autophagy but also suppresses excessive autophagy. This sophisticated regulation ensures a balance based on the stress’s intensity and duration. These findings have important implications for AD, where both too little and too much autophagy are linked to neurodegeneration [311].

Integration with the unfolded protein response (UPR): SKN-1 plays a role in ER stress responses by collaborating with UPR transcription factors XBP-1 and ATF-6. When ER stress or misfolded protein accumulation occurs, these factors work together with SKN-1 to activate genes for chaperones (HSPs), ERAD components, and proteasome subunits. This synergy helps restore proteostasis during protein-folding stress, a key feature of AD pathology [307].

### 7.6. Role in Aging and Longevity

SKN-1 is a key determinant of aging rate and maximum lifespan [308]. Loss-of-function mutations in *Skn-1* cause premature aging (shortened lifespan by 25–35%), while overexpression of *Skn-1* modestly extends lifespan (typically 10–20% extension) [306,308]. Several mechanisms contribute to SKN-1’s pro-longevity effects:

Glutathione homeostasis: SKN-1 maintains cellular glutathione (GSH) pools by upregulating γ-GCS and related biosynthetic enzymes. During aging, GSH levels naturally decline, contributing to increased oxidative damage. SKN-1 activation prevents this age-related GSH depletion through a mechanism distinct from IIS or dietary restriction pathways, suggesting that GSH homeostasis represents a unique SKN-1-mediated aging mechanism [306].

Age-related decline in SKN-1 activity is a key finding, showing that SKN-1 function worsens as organisms age [306]. The baseline expression of many SKN-1-controlled genes decreases gradually over time, and the immediate transcriptional response of SKN-1 to oxidative stress is significantly weaker in older worms than in young adults. This decline with age likely contributes to the increased oxidative stress observed in aging organisms. Research is ongoing to uncover the molecular mechanisms behind SKN-1 dysfunction with age, such as changes in post-translational modifications, diminished nuclear import processes, or epigenetic silencing, all of which have direct relevance to age-related AD [312].

The vital importance of SKN-1 in lifespan extension under reduced IIS is supported by genetic studies. These studies show that *Skn-1* is essential for the lifespan increase seen in reduced IIS conditions. For instance, mutations in *daf-2* (which encodes the insulin receptor) can increase lifespan by two to three times, but this extension is significantly diminished by about 30–35% when Skn-1 is knocked down. Likewise, lifespan extension achieved through *akt* RNAi heavily relies on SKN-1, highlighting its key role in the metabolic regulation of aging [313].

## 8. Transgenic *C. elegans* Models of Alzheimer’s Disease

### 8.1. Aβ-Expressing Models: Classical Strains and Recent Innovations

Classical Aβ models involve the traditional *C. elegans* AD setup, where the human Aβ_1–42_ peptide (actually Aβ_3–42_ due to signal peptide cleavage during translation) is expressed under a muscle-specific promoter (*unc-54*), causing accumulation in body wall muscles. The most common strain, CL2006, develops progressive adult-onset paralysis at higher temperatures (20 °C) and shows a rolling phenotype, an alternative movement pattern that makes it easy to identify transgenic animals visually. This model has been key in establishing paralysis as a measurable indicator of Aβ toxicity. However, CL2006’s variable penetrance—meaning not all worms become paralyzed, and the timing varies between experiments—limits its effectiveness for high-throughput screening [314].

A closely related strain, CL2120, contains the same *unc-54::Aβ* transgene but also expresses green fluorescent protein (GFP) driven by the intestinal *mtl-2* promoter. This allows for real-time visualization of Aβ expression and distribution using fluorescence microscopy [294]. CL2120 is especially useful for screening compounds because it enables researchers to observe both paralysis phenotype and Aβ buildup simultaneously, offering insights into how different compounds influence Aβ pathology [294].

A brief summary of several selected transgenic strains used in AD studies is presented in Table 4.

Temperature-inducible models: To address penetrance challenges, researchers created CL4176, which contains the *myo-3::Aβ* transgene regulated by an inducible promoter. These worms maintain normal movement throughout their lifespan at 16 °C. When larvae are shifted to 25 °C, the transgene is strongly activated, causing quick and complete paralysis within 4–6 h. This timing control is highly valuable: it enables studying the immediate effects of Aβ toxicity regardless of age, allows for targeted pharmacological interventions, and facilitates measurement of latency to paralysis (PT50 time to 50% paralysis) as a key metric. The temperature-shift method has become the standard for fast, consistent Aβ toxicity screening [315].

Neuronal-specific Aβ models: While models expressing Aβ in muscle cells offer insights into its direct toxicity, they do not fully capture effects specific to neurons. To improve this, transgenic strains that express Aβ in motor neurons under the *snb-1* (synaptobrevin) promoter have been developed, with CL2355 being the most well-studied [294]. These worms show defects in chemotaxis (attraction to odorants), impaired movement in liquid, and changes in neurotransmitter signaling. CL2355 is especially useful for exploring the synaptotoxicity of Aβ and associated memory or learning deficits, as chemotaxis acts as a proxy for associative memory and sensory processing [294].

Another specialized model, UA166, expresses Aβ_1–42_ in only five glutamatergic neurons under the *eat-4::GFP* construct, which simultaneously permits visualization of these neurons [294]. This ultra-focused expression system allows investigation of how Aβ disrupts glutamatergic synaptic transmission, the primary excitatory neurotransmitter system and a critical site of Aβ-induced toxicity [294].

Emerging optogenetic models: A cutting-edge development involves optogenetic control of Aβ oligomerization [294]. These transgenic strains express Aβ fused to light-sensitive domains, enabling blue-light-induced oligomerization and toxicity. By controlling the timing and localization of oligomer formation with blue light pulses, researchers can dissect the acute toxic effects of oligomers (which appear to be far more neurotoxic than fibrils) independent of secondary adaptations. Preliminary studies using this approach have revealed that Aβ oligomers, not fibrils, cause metabolic defects and mitochondrial dysfunction in neurons, a finding with significant implications for therapeutic target selection [294].

GMC101 strain and oligomer-specific approaches: The GMC101 strain has emerged as a leading model for studying Aβ oligomerization [294]. This strain provides advantages for oligomer-specific antibody compatibility and has been extensively used in recent chemotaxis and learning studies. Studies using GMC101 have demonstrated that plant-derived compounds (such as resveratrol) can improve cognitive function measured through chemotaxis-based memory assays, with some compounds achieving up to 214% improvement when optimized protocols are employed [316].

Early olfactory dysfunction in AD models: Recent studies demonstrate that olfactory dysfunction occurs very early in *C. elegans* AD models, preceding visible protein aggregation [317]. Both Aβ_1–42_ and polyglutamine-expressing models showed significant reductions in odor sensitivity in specific chemosensory neurons (AWB and AWC) as early as day 1 of adulthood. At the molecular level, ER unfolded protein response (UPR) is significantly activated in Aβ_1–42_-expressing worms. Activation of the AMPK pathway alleviates olfactory defects and reduces fibrillar Aβ in these worms, highlighting the importance of integrated proteostasis approaches [317].

### 8.2. MOAG-4/SERF as a Novel Therapeutic Target

A notable discovery in the study of Aβ aggregation is the identification of MOAG-4 (modifier of Aβ and glutamine toxicity), also called SERF (small ER-resident proteins of the Fln29 family). It acts as a cellular modifier that significantly speeds up Aβ nucleation and amyloid formation [318]. MOAG-4/SERF is a small, highly conserved, positively charged protein that interacts with negatively charged regions of amyloid-prone proteins via fuzzy complex formation. Interestingly, this interaction is quite non-specific: MOAG-4/SERF boosts the nucleation rates of various amyloid proteins, including Aβ_1–42_ and α-synuclein [318].

Mechanistically, MOAG-4/SERF binds to partially unfolded or partially aggregated amyloid monomers and oligomers, lowering the thermodynamic barrier to nucleation. Deletion of *moag-4* in transgenic Aβ-expressing worms substantially reduces amyloid puncta formation and delays paralysis, suggesting that inhibiting MOAG-4/SERF function—or blocking its interaction with Aβ—represents a novel therapeutic strategy. Unlike current amyloid-targeting therapies that aim to clear existing plaques, MOAG-4/SERF inhibition would prevent toxic oligomer formation at the source, potentially addressing the initial steps of amyloid pathogenesis. The conservation of MOAG-4/SERF from *C. elegans* to mammals indicates that insights from the worm model may directly inform therapeutic development for human AD [318].

### 8.3. Tau-Expressing Models: From Pan-Neuronal to Single-Copy Models

Classical tau models: *C. elegans* has an endogenous tau orthologue named *ptl-1* (patatin-like family member). Unlike mammalian tau, *ptl-1* is expressed only in embryonic epidermis and mechanosensory neurons and seems to play a neuroprotective rather than a neurotoxic role [298]. To model tauopathies, transgenic strains were created that express human tau (either wild-type or disease-associated mutants) under neuronal promoters. Recent studies show that APOE4 pan-neuronal expression causes patterned neurodegeneration that aligns with endogenous PTL-1 levels, and deleting PTL-1 suppresses defects in multiple behaviors, indicating broad protective effects in the nervous system [298].

The first-generation tau models expressed human tau with mutations linked to frontotemporal dementia and parkinsonism on chromosome 17 (FTDP-17), such as P301L, V337M, R406W, or ΔK280. These transgenes were controlled by either mechanosensory neuron-specific or pan-neuronal promoters. Phenotypic analysis showed that worms expressing wild-type human tau had a modest, progressive decline in touch response (the ability to respond to gentle anterior or posterior touch). In contrast, worms with mutant tau displayed severe symptoms: significant reduction in movement, accumulation of insoluble tau deposits, and notable decreases in touch response, along with neuritic abnormalities and microtubule loss [319].

V363 mutation models revealing presynaptic vs. postsynaptic effects: More recently, *C. elegans* models expressing tau with V363A or V363I substitutions were generated. These models revealed a striking functional dissociation: the V363A mutation induced presynaptic impairment affecting both motor and pharyngeal neurons, whereas V363I primarily caused postsynaptic defects resulting in motor neuron dysfunction. This synaptic-compartment-specific phenotypic variation suggests that tau’s pathogenic mechanism is context-dependent and that different tau mutants selectively compromise distinct neuronal compartments. These insights suggest that therapeutic strategies may need to be tailored to the specific tau mutation present in individual patients, a notion aligned with precision medicine approaches [320].

A152T models and acute neuronal dysfunction: Transgenic strains expressing A152T human tau at the neuronal level exhibit severe paralysis associated with soluble oligomeric tau species and acute neuronal dysfunction. Mechanistically, A152T tau impairs axonal trafficking, disrupting the microtubule-dependent transport of cargo essential for neuronal function. This model emphasizes the toxicity of soluble oligomeric tau, not just fibrils, and the primacy of impaired axonal transport in tau pathogenesis [321].

Mitochondrial dysfunction and mitophagy defects: To study tau’s effects on mitochondrial health, transgenic strains overexpressing human P301L tau or normal tau were generated. A key finding emerged: normal tau reduced mitochondrial autophagy (mitophagy), while P301L tau completely blocked mitophagy. This defect was quantified using the mito-mKeima fluorescent reporter, which specifically labels mitochondria undergoing autophagy. The tau-induced mitophagy impairment is particularly relevant to AD, where mitochondrial dysfunction and defective mitophagy are central pathogenic events. Natural products that enhance SKN-1-mediated mitochondrial biogenesis or selectively promote mitophagy (such as tomatidine) may counter this tau-induced pathology [322].

Dendra2::tau for tracking tau dynamics: A novel transgenic strain, Dendra2::tau, has recently been developed. This strain contains Dendra2, a photoswitchable fluorescent protein, fused to tau under pan-neuronal expression [294]. Dendra2 permits tracking of tau protein movements, aggregation, and clearance in vivo within intact animals. By illuminating specific neuronal regions with violet light (which converts the green fluorescence to red), researchers can photomark a cohort of tau molecules and track their movement and fate over time. This real-time tracking capability is revolutionizing our understanding of tau aggregation kinetics and clearance pathways in a physiologically relevant context [323].

### 8.4. Dual Aβ/Tau Co-Expression Models

A major limitation of individual Aβ or tau models is their inability to reflect the harmful interactions between these protein aggregates, which often coexist in AD brains. Recently, transgenic strains have been created that express both human Aβ and tau, either as separate transgenes or fused. These dual-transgene models demonstrate significant synergistic toxicity: they reduce lifespan more than either transgene alone, worsen behavioral deficits, and severely impair neurotransmitter signaling [324].

Transcriptomic analysis of worms expressing both Aβ and tau identified 248 genes upregulated and 805 downregulated compared to wild-type controls [324]. Notably, eight genes with altered expression in these worms overlapped with genes dysregulated in human AD brain tissues, offering cross-species validation [324]. Among the conserved dysregulated genes were *mgl-2* (human GRM1, metabotropic glutamate receptor 1) and genes related to collagen synthesis and modification (*dpy-18* and *phy-2*, human orthologs: P4HA2). These dual-transgene models are increasingly utilized to evaluate multi-target therapies, as they had better replicate the complex molecular pathology of human AD [325,326].

## 9. Effects of Plant Bioactive Compounds on Nrf2/SKN-1 Activation in AD Models

### 9.1. Modulation of Oxidative Stress and Antioxidant Response

Plant-derived bioactive compounds exert their neuroprotective effects against AD largely through activation of the Nrf2/SKN-1 antioxidant response pathway. The molecular mechanisms, however, vary considerably depending on the chemical structure and functional groups of each compound [327,328].

Polyphenolic compounds: Resveratrol, a stilbene polyphenol present in red grapes, berries, and peanuts, activates SKN-1/Nrf2 mainly by directly modifying KEAP1. It contains nucleophilic aromatic rings that target the critical cysteine residue Cys151 in KEAP1, preventing KEAP1 from tagging Nrf2/SKN-1 for ubiquitination and degradation. This direct interaction with KEAP1 sets resveratrol apart from many other antioxidants that activate Nrf2 indirectly. Additionally, resveratrol reduces Aβ aggregation and supports proteostasis by upregulating genes related to the proteasome and autophagy. In GMC101 transgenic worms, it improved chemotaxis-based cognitive function by 214% under optimal conditions, though the effect varies significantly with bacterial diet type (live or UV-killed) and environmental factors. Furthermore, resveratrol extends lifespan by activating SIRT1 and AMPK pathways, which intersect with Nrf2 signaling [316].

Curcumin, a natural pigment from turmeric (*Curcuma longa* L.), activates multiple signaling pathways to stimulate Nrf2/SKN-1 [329]. First, it inhibits NF-κB signaling, which is a key regulator of pro-inflammatory genes, leading to a decrease in inflammatory cytokines (IL-1β, IL-6, TNF-α) that can increase oxidative stress. Second, curcumin directly activates Nrf2 by modifying KEAP1 cysteines, similar to resveratrol [330]. Third, it increases the expression of antioxidant enzymes such as catalase (CAT), glutathione peroxidase (GPx), and superoxide dismutase (SOD) [5,330]. However, curcumin’s clinical use is limited by its poor bioavailability and absorption in mammals [5]. Ongoing efforts aim to develop derivatives with better solubility and stability, and several curcumin analogues are now in Phase II/III clinical trials for AD (ClinicalTrials.gov ID: NCT07612150).

Anthocyanins, water-soluble pigments in the flavonoid family, are abundant in berries, purple grapes, and dark leafy greens and are important neuroprotective compounds. These polyphenols have multiple hydroxyl groups and aromatic rings that give them strong antioxidant properties. They activate the Nrf2 pathway, increasing the expression of SOD1, GSTs, and catalase in brain tissues, including the hippocampus. Certain anthocyanins, like cyanidin-3-glucoside, help reduce oxidative stress and prevent caspase-dependent apoptosis in neuronal models exposed to Aβ toxicity [331].

In a *C. elegans* induced model of neurotoxicity, caffeic acid at a concentration of 25 mM restored the levels of Nrf2/ARE binding activity affected by the three toxins used to induce the model (quinolinic acid, 6 hydroxydopamine and FeSO_4_). In the wild-type *C. elegans*, but not in *SKN-1* KO mutant strains with a lacking orthologue of mammalian Nrf2, caffeic acid attenuated the loss of survival by approximately 20% and prevented the motor alterations induced by the toxins [332]. Paeonol, an abundant polyphenol compound in numerous medicinal herbs, significantly promoted the lifespan of *C. elegans* by approximately 29% at a dose of 200 µM. Moreover, paeonol promoted the health of *C. elegans* by increasing the body bending and pharyngeal pumping rates and reducing the lipofuscin accumulation level. Meanwhile, paeonol induced the expression of stress-related genes or proteins by activating the transcription factors DAF-16/FOXO, SKN-1/Nrf2, and HSF-1, which in turn enhanced oxidative and thermal stress tolerance. The mechanism behind the anti-aging effect of paeonol operated by downregulating the insulin/IGF-1 signaling (IIS) pathway [333]. Polyphenolic-rich fruit extract from *Canarium album* L. also prolonged the survival of *C. elegans* affecting IIS pathway, DAF-16/FOXO, and SKN-1/Nrf2 transcription factors. Treatment with the extract induced differential expression of genes related to defense response, immune response, stress response, oxidoreductase activity, and intracellular oxygen homeostasis in *C. elegans* [334]. The same pathways were modulated by treatment of *C. elegans* with methanolic extract of *Fragaria* x *ananassa cv. Romina* containing mainly ellagic acid and pelargonidin-3-glucoside. The extract was able to delay Aβ-protein-induced paralysis, reduced amyloid-β aggregation and prevented oxidative stress at doses of 100, 500 and 1000 µg/mL [335]. Berberine, isolated from *Coptis chinensis* Franch, has emerged as a promising candidate for anti-aging interventions, demonstrated by the lifespan-extending effects in *C. elegans.* The multi-target anti-aging effect was due to coordinated activation of stress-responsive pathways and metabolic optimization. Mechanistically it was explained by activating transcription factors such as DAF-16/FOXO, HSF-1, and SKN-1/Nrf2 and expression of antioxidant enzymes SOD-3, HSP-16.2, and GST-4 [336].

### 9.2. Regulation of Proteostasis and Protein Quality Control

An emerging and underappreciated effect of plant bioactive compounds is their capacity to enhance proteostasis, the cellular system maintaining protein homeostasis through quality control mechanisms including protein folding, aggregation prevention, and degradation [5,328,330]. This aspect is particularly relevant to AD, where proteostasis failure underlies Aβ and tau accumulation.

Proteasome-mediated protein degradation: SKN-1 enhances proteostasis by increasing proteasome subunit gene expression, supporting assembly and activity. Recent findings reveal several natural compounds can hyperactivate the 20S proteasome—the core catalytic part—independent of stress or UPR activation [337]. These compounds, like certain flavonoid extracts, specifically promote the breakdown of intrinsically disordered proteins (IDPs) such as Aβ oligomers and tau tangles [337]. Notably, proteasome hyperactivation decreases oxidative damage to cellular proteins without relying on superoxide dismutase (SOD), indicating a direct protein-clearance process separate from antioxidant enzyme activity. In aging studies, increased 20S proteasome activity closely correlates with lifespan extension and the reduction of vitellogenin buildup, a sign of aging and proteostasis decline [337].

Tomatidine, an alkaloid from tomato plants, influences mitochondrial health through a unique mechanism. It activates mitochondrial stress responses, known as mitohormesis, which ironically promote protective pathways. The production of mitochondrial ROS induced by tomatidine stimulates SKN-1 and SIRT1, resulting in the increased expression of mitochondrial biogenesis genes via the PGC-1α pathway. Importantly, in worms with tau expression, tomatidine restores impaired mitophagy by boosting SKN-1-dependent mitochondrial quality control. Additionally, healthspan indicators—such as pharyngeal pumping, swimming activity, and the extent of muscle cell damage—were all improved with tomatidine treatment [338].

Effects of autophagy-related compounds: Although SKN-1-driven upregulation of autophagy genes (ATG genes) is well-documented, the effects of different compounds on autophagy flux can differ. Some, like resveratrol and curcumin, generally boost autophagy, aiding in the removal of protein aggregates. Conversely, compounds that inhibit AMPK via SKN-1-mediated downregulation of aak-2 may decrease autophagy during extended stress periods. This nuanced response might be protective or harmful, depending on how long and intense the stress is [338].

UPR and ER stress response: Plant compounds stimulate SKN-1 alongside UPR factors (XBP-1, ATF-6), facilitating a coordinated proteostasis mechanism. For instance, certain flavonoid-rich plant extracts enhance *xbp-1* splicing and *atf-6* transcription while activating *Skn-1*, thereby boosting ER chaperone levels and ERAD enzyme expression [339].

### 9.3. Impact on Lifespan and Healthspan

Plant-derived compounds demonstrate remarkable lifespan-extending potential in *C. elegans*, with mechanisms varying by compound class [5,327,328,330].

IIS pathway-modulated lifespan extension: Compounds that downregulate IIS signaling components including daf-2 (insulin receptor), age-1 (PI3 kinase), and sgk-1 (serum/glucocorticoid-regulated kinase) phenocopy reduced IIS longevity [340]. Black rice anthocyanins achieve this through unclear mechanisms, possibly involving sequestration of IIS components or altered signaling dynamics. Once IIS is suppressed, SKN-1 and DAF-16 are dephosphorylated and accumulate in the nucleus, activating longevity programs. This class of compounds can extend lifespan by 20–40% depending on genetic background and diet [341].

Notable quantified effects: Amentoflavone (a biflavonoid from hyperforin) extends lifespan by 63.81% through robust DAF-16/FOXO activation, exceeding the effects of most single-target compounds [342]. Resveratrol extends lifespan, but the magnitude is context-dependent: lifespan extension is dramatic (20–30%) on live *E. coli* OP50 but much lower on UV-killed bacteria [343]. This diet-dependency underscores the importance of reporting exact experimental conditions in compound screening. Norharmane was recently identified as a therapeutic agent for mitigating muscle aging, improving swimming ability and increasing maximum velocity in *C. elegans* through SKN-1 activation. The study was validated using siRNA [344].

Healthspan metrics: Beyond simple lifespan, researchers increasingly measure “healthspan” indicators of tissue and physiological function throughout aging [337,342,343]. Key metrics include: Pharyngeal pumping frequency, which quantifies feeding and neuromuscular control. Aged worms show declining pump rates; compounds maintaining high pump rates extend healthspan. Swimming movement and body bends per minute, which assesses motor neuron control and muscle function. Compounds improving movement quality preserve healthspan. Muscle integrity, which is assessed by fluorescence microscopy or electron microscopy, measuring the percentage of severely damaged muscle cells. Compounds reducing muscle degeneration preserve healthspan. Stress resistance phenotypes, with compounds activating SKN-1 generally enhancing multiple stress resistance phenotypes. Heat stress, with improved survival at 35 °C or above. Oxidative stress, with enhanced survival of paraquat or H_2_O_2_ exposure. Osmotic stress, with improved tolerance to high salt levels [337,342,343].

However, an important nuance from recent studies is that Aβ exposure induces hormetic (adaptive) stress resistance to heat and anoxia but paradoxically *reduces* resistance to oxidative stress. This dissociation suggests that Aβ toxicity selectively impairs antioxidant defense while triggering non-specific stress adaptation—a distinction important for therapeutic targeting [317].

### 9.4. Cognitive and Behavioral Effects

Plant bioactive compounds improve multiple cognitive and behavioral domains disrupted in transgenic AD models. These effects are particularly critical for translational relevance, as cognitive decline is the hallmark symptom driving AD diagnosis and burden [317].

Paralysis assays and motor function: The temperature-inducible paralysis assay (using CL4176 worms) measures compound protection against Aβ-induced motor dysfunction. PT_50_ (time to 50% paralysis) serves as the primary metric: compounds extending PT_50_ slow neuromuscular degeneration. Rose essential oil (containing geraniol, linalool, and other volatile terpenes) achieved remarkable neuroprotection: PT_50_ was increased by 136.1% compared to vehicle control approaching the efficacy of the cognitive enhancer memantine. The mechanism involves SKN-1 activation and upregulation of *gst-4* mRNA, increasing detoxification capacity. This finding suggests that volatile plant compounds that are easy to administer via inhalation might have translational potential. The study was validated using strains with *skn-1* gene knocked down by RNAi [316].

Chemotaxis-based associative memory assays: CL2355 worms (expressing neuronal Aβ) exhibit deficits in chemotaxis—the ability to migrate toward attractive volatile odorants (typically isoamyl alcohol or benzaldehyde) and away from repellents [317]. The chemotaxis assay is interpreted as a proxy for associative learning and memory, as the olfactory system requires intact chemoreceptor neurons, synaptic transmission, and integration in sensory processing centers [317].

Mechanosensory function: Tau-expressing models (especially those with mechanosensory neuron-specific tau expression) show progressive decline in touch response—the reflex withdrawal from gentle anterior or posterior touch. This reflects degeneration of six pairs of mechanosensory neurons (ALMR, ALML, PLMR, PLML, AVM, PVM) and disruption of their synaptic connections. Compounds that activate SKN-1 can restore mechanosensory function [298].

5-HT hypersensitivity: CL2355 worms (expressing Aβ in motor neurons) develop hypersensitivity to serotonin (5-HT), exhibiting exaggerated behavioral responses to low concentrations of serotonin. This hypersensitivity reflects altered serotonergic neurotransmission, likely due to Aβ-induced changes in serotonin receptor expression or G-protein signaling. Plant compounds can reduce this 5-HT hypersensitivity, returning serotonergic responses to normal levels a finding suggesting neuroprotection of monoaminergic systems [345]. The effect of several plant-derived compounds/extracts is summarized in Table 5.

## 10. Translating Findings from *C. elegans* to Mammalian Models and Humans

### 10.1. Comparative Analysis of the Nrf2/SKN-1 Pathway

The remarkable conservation of Nrf2/SKN-1 function across phyla provides confidence that insights from *C. elegans* are translatable to mammals and humans, yet important differences exist that must be appreciated [308,309].

Functional conservation and divergence: The DIDLID domain shows high conservation between Nrf2 and SKN-1, being critical for both activating transcription and proteasomal degradation in both species. Nonetheless, there are important differences in DNA-binding strategies: mammalian Nrf2 needs to heterodimerize with Maf family proteins (c-Maf, MafF, MafG) to specifically and strongly bind ARE sequences. Conversely, *C. elegans* SKN-1 does not have this strict binding requirement and can function as a monomer or a homodimer. This difference likely stems from the simpler genome structure and fewer redundant ARE-related genes in *C. elegans* compared to mammals [307].

Despite their mechanistic differences, Nrf2 and SKN-1 mainly control similar sets of target genes, especially Phase II detoxification enzymes (such as GSTs, NAD(P)H quinone oxidoreductase, and glutamate cysteine ligase), Phase III exporters, and antioxidant genes including SOD, catalase, peroxiredoxins, and thioredoxin [308,309]. Studies on functional complementation have shown that mammalian Nrf2 can partly replace SKN-1 function in *C. elegans* mutants, indicating a significant level of functional conservation [309].

Tissue-specific regulation: A critical finding in recent years is that Nrf2 and SKN-1 exhibit tissue-specific regulation and cell non-autonomous effects in both organisms [309]. In mammals, intestinal epithelial Nrf2 controls systemic antioxidant defense and influences systemic inflammation through production of metabolites and neuropeptides. Similarly, *C. elegans* intestinal SKN-1 regulates neuromuscular junction function and neuronal stress resistance through multi-layered signaling cascades. This discovery underscores the importance of studying these pathways in intact organisms rather than isolated cells and suggests that therapeutic strategies targeting intestinal Nrf2 in humans might provide systemic neuroprotection [309].

### 10.2. Summary of Translational Evidence: Worm Discovery, Mammalian Validation, and Human Evidence

In Table 6 is presented a brief summary of several compounds/mechanisms. The direct *C. elegans*-to-human translational evidence is currently partial or absent, and that clinical evidence, where available, does not yet confirm the specific Nrf2/SKN-1 mechanistic link established in the worm.

### 10.3. Clinical Translation and Ongoing Trials

The promising preclinical findings in *C. elegans* have motivated human clinical trials of Nrf2-activating compounds for AD and related neurodegenerative diseases.

Resveratrol (Phase II completed): A pilot Phase II trial of resveratrol in AD patients (ClinicalTrials.gov ID: NCT00678431) has been completed [352,353]. The trial demonstrated that resveratrol is safe and well-tolerated in AD patients at doses of up to 1 g daily. CSF analysis revealed that resveratrol treatment reduced matrix metalloproteinase 9 (MMP-9), an enzyme implicated in BBB degradation and neuroinflammation. However, the modest sample size and bioavailability challenges of resveratrol (complicated by extensive hepatic metabolism) limit firm conclusions regarding cognitive efficacy [352,353].

Curcumin (Phase II/III ongoing): Curcumin is currently in Phase II/III trials (ClinicalTrials.gov ID: NCT03085680) for AD [328]. Multiple curcumin derivatives with enhanced bioavailability (liposomal formulations, nanoparticles) are being developed to overcome poor intestinal absorption and hepatic metabolism. Early results suggest cognitive stabilization in some patient populations, though definitive efficacy data are pending [328].

Sulforaphane (Phase II/III ongoing): Multiple sulforaphane trials are underway [355,356]. Two notable trials are NCT04213391 (testing sulforaphane efficacy in patients with prodromal to mild cognitive impairment due to AD) and NCT05233579 (assessing sulforaphane in premutation carriers). Sulforaphane activates Nrf2 through KEAP1 modification and exhibits robust anti-inflammatory effects in microglia, reducing production of IL-1β, IL-6, and inducible nitric oxide synthase (iNOS) [354]. Preclinical studies in transgenic AD mouse models (APP/PS1) demonstrate cognitive benefit and reduced Aβ burden following sulforaphane treatment [294].

Vitamin B6 (Pyridoxine), trial completed: A completed trial (ClinicalTrials.gov ID: NCT00056225) tested B vitamin supplementation (including pyridoxine/vitamin B6) in AD patients. The trial demonstrated that supplementation reduced homocysteine levels an independent risk factor for cognitive decline and AD and upregulated the Nrf2/HO-1 pathway, suggesting a mechanistic link between homocysteine reduction and antioxidant activation [357].

DL-3-n-Butylphthalide (NBP), Phase II/III ongoing: NBP is the only synthetic compound currently in Phase II/III trials specifically targeting Nrf2 for AD (ClinicalTrials.gov ID: NCT02993367). NBP is derived from l-3-n-butylphthalide, which is naturally present in celery (*Apium graveolens*) seeds. The racemic mixture (dl-NBP) was approved by China’s FDA for treatment of ischemic stroke in 2002, based on demonstrated neuroprotective efficacy. Mechanistic studies show that NBP improves synaptic plasticity, reduces vascular cognitive impairment, and enhances antioxidant defense in transgenic AD mouse models (APP/PS1). The mechanism involves Nrf2 activation, combined with improvements in vascular endothelial function and mitochondrial biogenesis [358].

### 10.4. Personalized Medicine Approaches Based on Genetic Stratification

Accumulating evidence suggests that individual genetic variation in Nrf2-related genes significantly modulates treatment response to Nrf2-activating compounds [360]. This finding opens possibilities for precision medicine in AD.

NFE2L2 (Nrf2 encoding gene) polymorphisms: Several single-nucleotide polymorphisms (SNPs) in *NFE2L2* have been associated with AD risk and biomarker profiles. Individuals carrying disease-associated alleles may show differential responses to Nrf2 activators, suggesting that genotyping could guide compound selection [360].

KEAP1 polymorphisms: KEAP1, the principal negative regulator of Nrf2, also harbors functionally relevant SNPs. KEAP1 variants affecting protein stability or KEAP1-Nrf2 binding affinity would directly affect the efficacy of KEAP1-targeting compounds like resveratrol and sulforaphane [360].

Environmental and lifestyle modifiers of Nrf2 activation: Beyond genetic factors, environmental variables substantially modulate Nrf2 pathway function.

Age: Nrf2 activity and SKN-1 expression decline with age, particularly in neurons. This age-dependent decline explains why Nrf-activating compounds may be maximally effective in earlier disease stages (mild cognitive impairment) rather than advanced AD [306,317].

Diet: long-chain polyunsaturated fatty acids (LC-PUFAs): Elegant studies have shown interaction effects between aging and dietary LC-PUFA supplementation (omega-3 fatty acids) on Nrf2 target gene expression [328]. High LC-PUFA intake enhances Nrf2-dependent antioxidant responses, suggesting that dietary intervention could be combined with pharmacological Nrf2 activators.

Exercise: Exercise-induced mild oxidative stress paradoxically activates adaptive Nrf2 responses, increasing antioxidant enzyme expression [328]. This hormetic effect suggests that structured exercise regimens might prime Nrf2 pathways, enhancing responsiveness to Nrf2-activating compounds.

Circadian rhythms: Recent evidence reveals that Nrf2 activation and signaling efficacy vary across the circadian cycle [293]. Evening-time Nrf2 activation is more effective at improving glycemic control and fatty acid oxidation compared to morning activation [293]. This finding suggests that chronotherapeutic dosing of Nrf2 activators, timing doses to match circadian peaks in pathway sensitivity, might enhance efficacy.

### 10.5. Challenges and Limitations in Extrapolating C. elegans Findings

Despite the remarkable conservation of AD mechanisms across species, important caveats must be acknowledged when translating worm findings to humans [294,298,300].

Lifespan and age compression: *C. elegans* lives only 3 weeks, whereas humans live 80+ years [294]. This lifespan compression means that neurodegeneration in worms proceeds at an accelerated rate and may activate different compensatory pathways than age-related neurodegeneration in humans. Effects that extend lifespan by 20–30% in worms (adding 6–9 days) might not translate to clinically meaningful longevity benefits in humans [294].

Absence of adaptive immunity: The worm lacks T cells, B cells, and antibody responses, preventing modeling of immune-mediated neuroinflammation in AD [294,300]. While recent studies show that innate immunity mechanisms (p38 MAPK, TLR signaling) are sufficient to model key aspects of neuroinflammation in *C. elegans*, adaptive immune contributions to human AD pathogenesis cannot be fully captured [294,300].

Missing disease genes: Critical genes implicated in human AD particularly *APOE*, which encodes apolipoprotein E and is the strongest genetic risk factor for LOAD—are absent from the *C. elegans* genome [294,300]. While *C. elegans* lacks endogenous APOE, recent studies have found that heterologous expression of human APOE isoforms in worms partially restores age-dependent neurodegeneration phenotypes, demonstrating APOE4-induced patterned behavioral decline and suggesting that some APOE functions can be modeled [300].

Simplified neuroanatomy: The *C. elegans* nervous system has 302 neurons, whereas the human brain contains ~86 billion neurons organized into highly specialized regions (prefrontal cortex, hippocampus, entorhinal cortex) with complex connectivity patterns [294,300]. While the worm’s simplicity permits detailed connectomic analysis and precise manipulation, it cannot recapitulate the vulnerability of specific brain regions to AD pathology [294,300].

Bioavailability and drug metabolism: *C. elegans* drug uptake occurs through direct cutaneous absorption (passive diffusion) or ingestion of bacteria laden with compound [293]. This contrasts sharply with human drug administration (oral, intravenous, intrathecal), where absorption, metabolism, and BBB penetration are critical rate-limiting steps. A compound that protects *C. elegans* at high concentrations might fail in humans due to poor bioavailability [293].

## 11. Future Perspectives and Emerging Technologies

### 11.1. Advanced High-Throughput Screening Platforms

Recent technological innovations have revolutionized the capacity to conduct large-scale phenotypic screening in *C. elegans*, accelerating drug discovery and target identification [293,361].

Automated imaging and machine learning: Modern high-throughput screening systems employ automated microscopy with pattern recognition algorithms to quantify multiple neuronal and somatic phenotypes simultaneously [361]. Specialized software can measure paralysis onset, locomotion parameters (body bends, thrashing frequency, movement velocity), chemotaxis index, and Aβ aggregation levels from thousands of worms in parallel. Artificial intelligence/machine learning algorithms trained on large phenotypic databases can predict compound efficacy and mechanism of action from imaging data alone, substantially reducing experimental time [361].

Lifespan Machine and multiplexed aging studies: The Lifespan Machine uses standard flatbed scanners to image thousands of worms continuously throughout aging, generating precise lifespan curves and survival statistics from single experiments [293,361]. This technology has enabled large-scale, comparative studies of lifespan-extending compounds and genetic interventions, dramatically increasing the scale and statistical power of aging research [293,343].

NeuroChip and electrophysiology: Microfluidic devices such as NeuroChip permit precise physical isolation and electrophysiological recording from individual *C. elegans* neurons or synapses. These devices allow measurement of synaptic transmission, ion channel function, and action potential parameters providing functional neurophysiology data at single-cell resolution. For AD research, NeuroChip enables direct measurement of Aβ effects on synaptic transmission and assessment of compound protection against Aβ-induced synaptic dysfunction [361].

Small molecule accumulation (SAM) model for bioavailability prediction: A computational/experimental framework termed the SAM model predicts whether small molecules will accumulate to sufficient levels in *C. elegans* to exert biological effects [293,361]. The SAM model accounts for molecular weight, lipophilicity, polarity, and structural features that influence permeability. Importantly, SAM predictions correlate with human bioavailability and oral absorption, enabling early prioritization of compounds with promising absorption profiles [361].

### 11.2. Omics Technologies and Systems-Level Analysis

Beyond individual gene measurement, systems-level profiling reveals how Nrf2/SKN-1 activation rewires transcriptional networks, protein interactions, and metabolite pools [324].

Transcriptomics of Nrf2 pathway activation: Whole-genome RNA-sequencing (RNA-seq) of *C. elegans* exposed to Nrf2-activating compounds reveals the complete spectrum of genes regulated by Nrf2/SKN-1 [324]. Recent transcriptomics studies have identified novel SKN-1 target genes beyond the canonical detoxification enzymes, including genes involved in lipid metabolism, mitochondrial function, and cell–cell communication. Comparison of transcriptomics between *C. elegans* SKN-1-activated neurons and human AD brain tissue has identified conserved dysregulated gene modules, suggesting that findings in worms address relevant human pathophysiology [324].

Transcriptomics in dual Aβ/tau models: Dual transgenic worms expressing both human Aβ and tau provide systems-level insights into polyglot AD pathology [324]. Transcriptomic analysis revealed 248 significantly upregulated and 805 downregulated genes; critically, 8 genes showing altered expression in worms overlapped with genes dysregulated in human AD brain tissue, providing cross-species validation [324].

Proteomics and protein-interaction networks: Mass spectrometry-based proteomics can quantify global changes in protein abundance following Nrf2 activation, revealing both direct Nrf2 target gene products and secondary effects. Quantitative phosphoproteomics (phospho-MS) measures phosphorylation state changes on thousands of proteins, revealing which kinase cascades are engaged by specific compounds. Co-immunoprecipitation coupled to mass spectrometry (CoIP-MS) identifies physical protein–protein interactions altered by compound treatment, revealing unexpected mechanistic nodes [362].

Spatial transcriptomics: Emerging spatial transcriptomics technologies (MERFISH, seqFISH, Slide-seq) permit single-cell resolution mapping of gene expression within intact *C. elegans* tissues, particularly valuable for mapping tissue-specific Nrf2 responses and understanding cell–cell interactions in the neuronal microenvironment [363].

Metabolomics and fluxomics: Untargeted metabolomics reveals global shifts in metabolite pools following Nrf2 activation or compound treatment [310,328]. Recent studies show that Nrf2/SKN-1 activation during fasting couples proline catabolism (generating α-ketoglutarate for the TCA cycle) with fatty acid oxidation, a metabolic integration that maintains energy production [310]. Metabolic flux analysis further quantifies the relative contribution of different nutrient oxidation pathways, revealing how compounds reshape cellular metabolism [310].

### 11.3. Chronotherapeutic Approaches and Circadian Regulation

An underappreciated frontier is the integration of circadian biology with Nrf2-targeted therapeutics [293]. Recent research shows that Nrf2 protein levels, phosphorylation, and gene activity follow circadian rhythms in both *C. elegans* and mammals. The clock proteins CLOCK and BMAL1 (known as Period2 in worms) directly influence *NFE2L2* and antioxidant enzyme gene transcription, establishing a circadian pattern in antioxidant defenses. Notably, sensitivity to oxidative stress changes throughout the cycle cells are least vulnerable during nighttime in nocturnal animals (or daytime in humans) [364].

Chronotherapeutic optimization: Administration of Nrf2-activating compounds at times of day when Nrf2 pathway sensitivity is maximized could substantially enhance therapeutic efficacy [293]. Studies in humans show that evening-time Nrf2 activation (around 8:00 p.m.) is more effective at improving glycemic control, reducing circulating glucose, and increasing fatty acid oxidation compared to morning administration [293]. This circadian pharmacodynamics suggests that clinical trials of Nrf2 activators should test chronotherapeutic dosing timing drug administration to match circadian peaks in pathway activity [293].

### 11.4. Integration of Nrf2 Activation with Multi-Target Therapies

Given the failure of single-target anti-AD drugs (particularly anti-amyloid therapies such as aducanumab), multi-target approaches combining Nrf2 activation with complementary mechanisms are receiving intense focus.

Nrf2 + anti-amyloid monoclonal antibodies: Recent clinical approvals (aducanumab, donanemab, lecanemab (NCT03367403, NCT04437511, NCT03887455) target Aβ directly. Preclinical studies in *C. elegans* and mouse models suggest that combining Nrf2 activators with anti-amyloid antibodies produces synergistic cognitive benefit: antibodies clear existing plaques while Nrf2 activators reduce oxidative stress, support proteostasis, and restore mitochondrial function. *C. elegans* provides an ideal platform to test such combinations before expensive clinical trials [359].

Nrf2 + anti-tau therapies: Tau-targeting approaches (including tau-directed antibodies and tau aggregation inhibitors) are advancing. Combining tau-targeting with Nrf2 activation addresses orthogonal pathogenic mechanisms: antibodies disrupt tau seeding while Nrf2 activation enhances cellular capacity to clear tau oligomers and restore proteostasis. *C. elegans* dual Aβ/tau models enable testing of such combinations [365].

Nrf2 + mitochondrial-targeted therapies: Given that mitochondrial dysfunction is central to AD pathogenesis, combining Nrf2 activators (which support mitochondrial biogenesis through PPARγ/PGC-1α signaling) with mitochondrial-targeted antioxidants or mitophagy enhancers is rational. This combination addresses both mitochondrial biogenesis and quality control [366].

Nrf2 + anti-inflammatory strategies: NSAIDs (inhibitors of COX enzymes, which produce pro-inflammatory prostaglandins) exert neuroprotective effects through both canonical (prostaglandin reduction) and non-canonical mechanisms. Recent evidence reveals a noncanonical Nrf2-dependent mechanism of NSAID action, suggesting that combining NSAIDs with direct Nrf2 activators might not provide additional benefit, but rather represents mechanistic overlap an important consideration for trial design [367].

Nrf2 + stem cell therapy: Neural stem cell (NSC) transplantation is being explored as a regenerative strategy for AD. Preliminary studies suggest that co-administration of neural stem cells with Nrf2-activating compounds produces synergistic neuroprotection: stem cells provide trophic support and neural replacement, while Nrf2 activation reduces inflammation and supports stem cell survival and integration [368].

### 11.5. Novel Therapeutic Targets Emerging from C. elegans Research

Beyond Nrf2/SKN-1 itself, *C. elegans* research has identified upstream and downstream targets amenable to therapeutic intervention [318,369].

MOAG-4/SERF inhibition: As discussed, MOAG-4/SERF accelerates amyloid nucleation [80]. Unlike current therapies targeting end-stage plaques, MOAG-4/SERF inhibition would prevent toxic oligomer formation at the source. Chemical libraries are being screened against MOAG-4/SERF for inhibitory compounds, with *C. elegans* serving as a primary validation platform [318].

WDR-23 isoform-specific targeting: The discovery that WDR-23 isoforms have opposite effects on SKN-1 suggests a therapeutic strategy selective inhibition of the nuclear repressive WDR-23B isoform (while preserving the activating WDR-23A isoform) could enhance SKN-1 activation without triggering constitutive, potentially maladaptive signaling. Chemical tools targeting isoform-specific function are in development [369].

Fine-tuning SKN-1 phosphorylation: The AKT-1, AKT-2, and SGK-1 kinases phosphorylate SKN-1, preventing nuclear accumulation [306]. Selective inhibition of these kinases specifically at SKN-1 target sites (while preserving their other essential functions in growth and metabolism) could enhance SKN-1 activation. Phosphatases (e.g., PP2A) that dephosphorylate SKN-1 are being investigated as therapeutic targets [353].

AMPK/SKN-1 axis for autophagy modulation: Understanding how SKN-1 suppresses AMPK during prolonged stress could reveal therapeutic targets to fine-tune autophagy intensity promoting early, protective autophagy while preventing excessive, pathologic autophagy [311].

## 12. Conclusions

*Caenorhabditis elegans* has become a valuable and increasingly advanced model for studying Nrf2/SKN-1 pathway activation in AD. Its high conservation from worms to humans, along with practical benefits such as a short lifespan, small size, transparent nervous system, ease of genetic manipulation, and emerging high-throughput methods, make *C. elegans* ideally suited to quickly screen plant-based bioactive compounds, uncover neuroprotective mechanisms, and validate potential therapeutic targets.

This review summarizes current knowledge showing that plant-derived polyphenols, including anthocyanins and specialized compounds like resveratrol, curcumin, sulforaphane, guarana, and tomatidine, activate the Nrf2/SKN-1 pathway in *C. elegans* AD models. This activation offers broad neuroprotection, such as reducing oxidative stress, improving proteostasis, extending lifespan and healthspan, and restoring cognitive and behavioral functions. Mechanistically, these compounds target multiple regulatory points—from directly modifying KEAP1/WDR-23 and post-translationally modifying SKN-1 to reprogramming metabolism—indicating that Nrf2/SKN-1 is not a simple “on/off” switch but a complex hub integrating responses to oxidative stress, metabolic state, and inflammation.

Clinical translation progresses with several Nrf2-activating compounds in Phase II/III trials. Emerging evidence indicates that personalized medicine approaches tailoring treatments based on Nrf2 pathway genetics and environmental factors like age, diet, exercise, and circadian phase will enhance therapeutic outcomes. Future efforts include combining Nrf2 activation with multi-target strategies that address amyloidopathy, tauopathy, mitochondrial dysfunction, and neuroinflammation simultaneously. Additionally, leveraging emerging technologies such as high-throughput screening, omics, optogenetics, and chronotherapeutics can speed up discovery. Novel targets identified through *C. elegans* research like MOAG-4/SERF, WDR-23 isoforms, and SKN-1 phosphorylation sites offer promising therapeutic avenues.

The combination of evolutionary conservation, mechanistic feasibility, and translational potential makes Nrf2/SKN-1-targeted strategies promising for disease-modifying Alzheimer’s therapy. As research advances and compounds are tested in increasingly complex models from *C. elegans* to rodents, non-human primates, and humans, Nrf2-based treatments could ultimately provide significant cognitive improvements and slow disease progression in patients affected by this severe neurodegenerative disorder.

## Figures and Tables

**Table 1:** List of target genes regulated by Nrf2, according [141,170,173,175].

General Function	Gene Symbol	Name
Drug metabolism and disposition		
Oxidation	ADH7	Alcohol dehydrogenase 7
	ALDH3A1	Aldehyde dehydrogenase
	CYP2A5	Cytochrome P450
Reduction	AKR1B3	Aldo-keto-reductase
	CBR1	Carbonyl reductase 1
	NQO1	NAD(P)H quinone oxidoreductase
Conjugation	SULT3A1	Sulfotransferase
	UGT1A1	UDP-glucuronosyltransferase
Nucleophilic trapping	GSTA2	Glutathione S-transferase
	EPHX1	Epoxide hydrolase
	ES-10	Esterase
Drug transport	MRP2	Multidrug resistance-associated protein
Antioxidant defense		
ROS catabolism	GPX2	Glutathione peroxidase
	PRX1	Peroxiredoxin
	SOD3	Superoxide dismutase 3
Regeneration of oxidized factor	GSR1	Glutathione reductase
	SRX1	Sulfiredoxin
	TRXR1	Thioredoxin reductase
Synthesis of reducing factor	GCLC	Glutamate-cysteine ligase, catalytic subunit
	GCLM	Glutamate–cysteineligase, modifier subunit
Antioxidant protein and inhibitor	TRX	Thioredoxin
	TXNIP	Thioredoxin interacting protein
Redox transport	SLC7A11	Cystine/glutamate transporter
Metal-binding protein	MT1	Metallothionein
	FTL	Ferritin
Stress response protein	HO-1	Heme oxygenase
Oxidant signaling and function		
Autophagy	p62	p62 protein
	ATG5	Autophagy protein 5
	ATG7	Autophagy protein 7
	LC3B	Microtubule-associated protein1A/1B-lightchain3B
Mitochondrial apoptosis	PARK7	Parkinson’s disease 7
Mitochondrial biogenesis	Nrf1	Nuclear respiratory factor 1
Growth factor signaling	PTP1	Protein tyrosine phosphatase
	PTPRB	Protein tyrosine phosphatase receptor
Metabolism		
Carbohydrate metabolism and NADPH generation	G6PD	Glucose-6-phosphatedehydrogenase
	HDK1	Hexokinase domain containing 1
	IDH1	NADP-dependent isocitrate dehydrogenase
	MEI1	Malic enzyme 1
	PGD	6-phosphogluconate dehydrogenase
	TALDO1	Transaldolase
	TKT	Transketolase isoform 1
Lipid metabolism	ACOT7	Acetyl-CoA thioesterase 7
	ACOX1	Acetyl-CoA oxidase 1
	SCD2	Stearoyl-CoA desaturase 2
Heme and iron metabolism	BLVRA	Biliverdin reductase A
	BLVRB	Biliverdin reductase B
	FTH1	Ferritin, heavy polypeptide
	FTL1	Ferritin, light polypeptide
	HO-1	Heme oxygenase 1
Proteasomal degradation		
Regulator of cellular responses to stress	ATF4	Activating transcription factor-4
	PSMA1	Proteasome subunit alpha type-1
	PSMB5	Proteasome subunit beta type-5
	p62	p62 protein
Apoptosis and inflammation		
Apoptosis	BCL-2	B-celllymphoma 2
Inflammation	A1AT	α1-antitrypsin
	SLPI	Secretory leukoprotease inhibitor
	MARCO	Macrophage receptor with collagenous structure
	IL-17D	Interleukin 17D
	CD36	Cluster of differentiation 36
	IL6	Interleukin 6
	IL1β	Interleukin 1β

**Table 2:** Plant species and plant-derived molecules able to modulate the Nrf2 pathway in a variety of experimental in vitro and in vivo models.

Phytochemical/Plant Species	Disease Model	Dosage and/or Duration	Study Observations	Nrf2 Related Mechanism	Reference
Polyphenols/polyphenols rich plant extracts					
Acertannin	LPS-induced inflammation in RAW246.7 cells	100 µM	↓ NF-κB, ROS, TNF-α, IL-12, iNOS, COX-2	↑ Nrf2, HO-1	[246]
Arbutin	Lysolecethin-induced demyelination model in rats	50 mg/kg for 2 weeks	↓ IL-1β, IL-17, TNF-α, iNOS; ↑ IL-10	↑ Nrf2, HO-1	[247]
Avenanthramide-2c	Oxidative stress induced in PC12 cells	20–40 µM	↑ Trx, TrxR, GCLM, GCLC	↑ Nrf2/ARE, NQO1, HO-1	[248]
Curcumin	Neurodegeneration and neurotoxicity in *Drosophila melanogaster*	1 mg/g for 7 days	↓ ACE1	↑ Nrf2, CAT	[249]
Eleutheroside B	High-altitude pulmonary edema in rats	50 and 100 mg/kg/day for three days	↓ TNF-α, IL-1β, VEGF, COX-2	↑ Nrf2, HO-1	[250]
Epigallocatechin-3-gallate	Cisplatin-induced brain toxicity in Wistar albino rats	40 mg/kg/day for 4 weeks	↓ NF-κB, Bax; ↑ Bcl-2; protected against cisplatin-induced inflammatory burden	↑ Nrf2, HO-1	[251]
Grape seed extract	Methotrexate-induced hepatic inflammation in BALB/c mice	75 and 125 mg/kg/day for 14 days	↓ NF-κB/NLRP3, IL-1β, iROS, LPO	↑ Nrf2, HO-1, SOD	[252]
*Gnaphalium affine* D. Don	H9c2 H_2_O_2_-induced model of myocardial injury	50 µg/mL	↓ PI3K/AKT/GSK-3β	↑ Nrf2, SOD	[253]
*Inula britannica* L.	H_2_O_2_-induced inflammation in SH-SY5Y cells		↓ NF-κB, Bax; ↑Bcl-2	↑ Nrf2/KEAP-1, HO-1, NQO1	[254]
*Lycium barbarum* L.	Ethanol-induced cognitive impairment in mice	100, 200 and 400 mg/kg/day for 38 days	↑ Proliferation of neural stem cells, neural progenitor cells and neuroblasts	↑ Nrf2, HO-1	[255]
*Morus nigra* L.	Cigarette smoke-induced inflammation in hypertensive rats	300 mg/kg/day for two months	↓ JAK2/STAT1, Duox1-2	↑ Nrf2/KEAP-1	[256]
Oligonol	Dimethylnitrosamine-induced chronic liver damage in male Sprague–Dawley rats.	10 and 20 mg/kg/day for four weeks	↓ NF-κB, TNF-α, IL-1β, IL-6, COX-2, iNOS, TGF-β1, α-SMA	↑ Nrf2	[257]
Pterostilbene	Diabetic model of human endothelial cells (EA.hy929)	10 µM	↓ HDACs I-IV, DNMTs 1/3A and 3B,	↑ Nrf2/ARE, NQO1, HO-1, SOD, CAT	[258]
Resveratrol	C57BL/6 thoracic blast mouse model	25 and 50 mg/kg/day for 10 days	↓ NF-κB, ROS, IRE-α, CHOP	↓ Nrf2 and KEAP1 induced expression in the brain	[259]
Resveratrol	Oxidative damage induced in heat-stressed broilers	400 mg/kg/day for 7 days	↓ HSP70, p62	↑ Nrf2/KEAP-1; ↑ GRX, SOD, HO-1	[260]
Rosmarinic acid	Neuronal cell model in PC12 cells	0.3–100 µM	↑ Akt phosphorylation; ↑ GSK-3β, ↓ ROS, LPO	↑ Nrf2 nuclear translocation; ↑ HO-1, NQO1, GCLc, TrxR	[261]
*Securidaca inappendiculata* Hassk	Diabetic encephalopathy induced in rats	50–200 mg/kg for 8 weeks	↓ NF-κB, AChE, caspase-3, MDA, p38, MAPK	↑ Nrf2, HO-1	[262]
Taxifolin	Pre-cancer induced model in JB6+cells	10–40 µM	↓ DNMT, HDAC	↑ Nrf2, HO-1, NQO1	[263]
*Theobroma cacao* L.	MMP^+^-intoxicated SH-SY5Y cell line	3 and 10 µg/mL	↑ PPRγ, PGC1α, TFAM, Mfn2; ↓ Fis1	↑ Nrf2	[264]
Tea polyphenols	Radiation-induced intestinal injury in C57BL/6 mice	100–160 mg/kg/day for 5 days	↓ NF-κB, ferroptosis, ROS,	↑ Nrf2, HO-1, GPX4	[265]
Thonningianin A/*Penthorum chinense* Pursh	Model of PD in zebrafish	0.3–2.5 µg/mL	↓ Ferroptosis, α-syn, MDA; ↑ total swimming distance, GSH	↑ Nrf2/KEAP-1	[266]
*Vitis vinifera* L.	AlCl_3_-induced AD model in Wistar rats	15 mg/kg/day for 30 days	↓ p-Tau, APP, Aβ plaques, ROS, MDA	↑ Nrf2, HO-1, CAT, SOD, GSH	[267]
Alkaloids/alkaloids rich extracts					
Berberine	Model of induced depression in rats	50 and 100/day mg/kg	↓ MDA	↑ Nrf2/KEAP-1, SOD	[268]
Berberine	Model of AD in mice	50 mg/kg/day for 7.5 months	↓ Aβ plaque, hyperphosphorylated tau protein, neuronal loss, and ferroptosis in the brains, MDA, ROS	↑ Nrf2/KEAP-1, SOD, GSH, GPX4 and SLC7A11	[269]
Berberine	Neurodegeneration in C17.2 cells	13.5 µM	↓ Caspase-3, Bax; ↑ ERK, p-ERK, Bcl-2, ASCL1, NeuroG1, NeuroD2 and DCX	↑ Nrf2/KEAP-1, NQO1, HO-1	[270]
Fangchinoline	Cognitive impairments in mouse neuroblastoma N2A cells	2.5 µM	↓ Cognitive impairment	↑ Nrf2, HO-1, GSH-PX, SOD	[271]
Hecubine	Bipopolysaccharide-induced neuroinflammation in zebrafish	25 µM	↓ NO, TNF-α, IL-6, IL-1β	↑ Nrf2	[272]
Norboldine	AD model in 3 × Tg mice	1–5 µM	↓ ROS, Aβ deposits	↑ AMPK/GSK3β/Nrf2	[273]
*Oxalis corniculata* Linn	Model of AD in rats	150 mg/kg/day for 5 weeks	↓ Neuroinflammation, TLR4/NF-κB/NLRP3	↑ Nrf2, HO-1	[274]
Isothiocyanates/Isothiocyanate rich extracts					
*Brassica oleracea var. italica* sprout extract	Scopolamine-induced mouse model of memory impairment	100 and 200 mg/kg/day for 4 weeks	↑ Scopolamine-induced deficits in long-term memory; ↓ striatal choline acetyl transferase expression, NF-κB, TNF-α, COX-2	↑ Nrf2	[275]
Sulforaphane	Vascular cognitive impairment in rats	10 mg/kg/day for six weeks	↓ Aβ and tau accumulation, neuronal death, ↑ Akt/GSK3 β	↑ Nrf2/ARE, HO-1	[276]
Sulforaphane	Immortalized cortical neuronal model (CN 1.4 cells)	10 µM	↓ ROS, mitochondrial abnormalities	↑ NQO1, HO-1, GCS, GR1 and TRxR1	[277]
Phenylethanoid glycosides					
Acteoside	Parkinson’s disease model in SH-SY5Y cells	50 µM	↓ Ferroptosis; ↓ MDA, ↑ SLC7A11; ↑ GPX4; ↑ GSH	↑ Nrf2-mitophagy signaling pathway	[278]

**Table 3:** Comparison of major model organisms used in AD research [295,296].

Characteristics	Primates	Rodents	Zebrafish	*Drosophila melanogaster*	*Caenorhabditis elegans*
Advantages	- Most human-like - AD-like pathology and symptoms	- Similar brain anatomy to humans - Monitoring of the treatment by histopathology and behavioral tests	- Vertebrate animal - Easy to bread with no ethical limitations - Possess a complete immune system - Fully sequenced genome - In vivo live imaging - Gene homology close to humans - HTS possible	- Easy to bread with no ethical limitations - Short lifespan and generation time - Genetic manipulations are straightforward - HTS possible	- Easy to bread with no ethical limitations - Least lifespan - Possible genetic manipulation - Maximum HTS - RNA interference allows inactivation of thousands of genes in parallel - Better live imaging compared to Drosophila and zebrafish
Limitations	- Ethical considerations - Limits in animal number - HTS not possible - Expensive maintenance - The animals age over an extended period	- Ethical considerations - Limits in animal number - HTS not possible - Expensive maintenance - No neurofibrillary bundles are formed	- Expensive maintenance - Challengeable genetic modification - Limited cognitive behavior tests	- Different brain anatomy compared to humans - Less adaptive immune system - Genes are less orthologous to humans - Simple behavior and weak cognitive abilities - Neurofibrillary and plaque staining, regional vulnerability impossible to address	- Different brain anatomy compared to humans - Simple behavior and weak cognitive abilities - Neurofibrillary and plaque staining, regional vulnerability impossible to address - Limited homologous gene compared to humans

**Table 4:** Summary of several strains, related proteins, transgene expression and phenotype of *C. elegans* models of AD [284,288,300].

*C. elegans*	Genotype	Transgene Expression	Phenotype	Proteotoxic Process
Aβ models				
CL2006	*dvIs2* [pCL12(*apl-1*/human Aβ peptide 1–42 minigene) + pRF4]	Constitutive expression in muscle cells	Progressive age-dependent paralysis with formation of Aβ amyloid deposits	Toxic buildup and clumping of human Aβ_1–42_ proteins in muscle cells
CL4176	*dvIs27* [*myo-3p*::Aβ (1–42)::*let-851* 3′UTR) + *rol-6*(*su*1006)] X	Inducible expression in muscle cells	Rapid paralysis and oxidative stress which precedes Aβ fibril formation	Temperature-triggered expression and toxic buildup of human Aβ_1–42_ peptides in muscle cells, which leads to severe cellular stress and paralysis
CL2122	*dvIs15* [(pPD30.38) *unc-54*(vector) + (pCL26) *mtl-2*::GFP]	Constitutive expression in muscle cells	Slow adult movement. Intramuscular Aβ deposits.	Does not undergo a disease-related proteotoxic process (Control for Aβ muscles)
CL2355	*dvIs50* [pCL45 (*snb*-1::Aβ_1–42_::3′ UTR(long) + *mtl*-2::GFP] I	Inducible pan-neuronal expression	Defective chemotaxis, formation of amyloid deposits, and serotonin hypersensitivity	Harmful accumulation and clumping of Aβ_1–42_ proteins inside nerve cells
UA166	[*baInl32*; P*eat-4*::ssAβ_1–42_,P*eat-4*::gfp, P*myo*-2::mCherry]	In glutamatergic neurons	Age-dependent loss of glutamatergic neurons	Neuron-specific expression of human Aβ_1–42_ driven by the *eat-4* promoter leading to an age-dependent aggregation of the peptide and selective loss of glutamatergic neurons
GMC101	*dvIs100* [*unc-54p*::Aβ-1–42::*unc-54* 3′-UTR + *mtl-2p*::GFP]	Inducible body wall muscles	Severe paralysis following 48 h exposure to 25 °C temperature shift	Temperature-induced expression and aggregation of human Aβ_1–42_ in muscle cells, which causes severe protein insolubility, mitochondrial stress, and progressive paralysis
APP model				
ynIs109	P*snb-1*::*apl-1* cDNA::GFP	Pan-neuronal *apl-1* over expression	Impaired touch habituation	The apl-1 protein is expressed throughout the nervous system
JPS67	*vxSi38* [P*rab-3*::huAPP695::*unc-54*UTR, Cb *unc-119*(+)]	Pan-neuronal *apl-1* over expression	Behavioral deficits such as impaired locomotion and mobility	Pan-neuronal expression of the human amyloid precursor protein (hAPP695) generating Aβ peptides that misfold, aggregate, and cause cellular toxicity
Tau models				
BR5270	*byIs193*[P*rab-3*::F3∆K280; P*myo*-2::mCherry]	Constitutive pan-neuronal	Severe locomotive impairment at day 1 of adulthood; accelerated aggregate formation; severe developmental defects in nervous system; impairments in presynaptic transmission	Clumping of a human tau protein fragment, leading to nerve cells damages and ruins movement over time
BR5271	*byIs162*;[P*rab-3*::F3∆K280(I277P)(I308P); P*myo-2*::mCherry]	Constitutive pan-neuronal	No overt locomotive impairment; minimal effects on neurodevelopment	Control strain that expresses non-aggregating variant of human tau and does not experience severe proteotoxicity seen in pro-aggregant disease models
N.A. *	*Is*[*Paex-3*::4R1N; P*myo-2*::mCherry]	Pan-neuronal expression	Progressive paralysis and motor deficits	Triggers a specific sequence of proteotoxic stress and neurodegeneration
N.A.	*bkIs10*[P*aex-3*::h4R1 NTauV337M;P*myo*-2::gfp]	Pan-neuronal expression	Progressive tau aggregation and neuronal dysfunction	The human tau protein is expressed throughout the nervous system
N.A.	*Is*[P*aex-3*::P301L; P*myo-2*::mCherry]	Pan-neuronal expression	Progressive paralysis and motor deficits	The proteotoxic process involves protein misfolding, aggregation, proteotoxic stress and cellular dysfunction

* N.A.—non-provided strain nomenclature from the authors.

**Table 5:** Comprehensive efficacy table of plant bioactive compounds.

Compound/Extract	Strain(s)	Primary Effects	Mechanism(s)	Quantified Efficacy	Reference
Rose essential oil *	CL4176, CL2355	PT_50_ ↑, 5-HT normalization, reduced Aβ deposits	SKN-1 activation, ↑ *gst-4* mRNA, antioxidant terpenes (geraniol, linalool)	PT_50_: 136.1% ↑ vs. vehicle	[316]
**Black rice anthocyanins**	CL4176, CL2355	PT_50_ ↑, chemotaxis rescue	IIS pathway ↓, SKN-1 dephosphorylation, ↑ antioxidant defenses	Chemotaxis: 99.96% improvement; PT_50_: significant delay	[341]
**Lycopene**	GMC101	Reduced Aβ secretion and deposits	SKN-1 activation,	Aβ deposit reduction: 50–70%	[346]
Curcumin and hemi-analogs *	GMC101	Aβ toxicity protection, paralysis delay	↑*Skn-1* mRNA, NF-κB ↓, KEAP1 modification	Lifespan: 15–20% extension	[347]
**Anthocyanin-rich purple wheat extract**	Mev-1 (WT background)	Lifespan ↑	IIS pathway ↓, ROS ↓	Lifespan: 20–30% extension	[348]
**Amentoflavone**	WT and aging models	Lifespan ↑	DAF-16/FOXO activation, ↑ *sod-3*	Lifespan: 63.81% extension	[342]
Norharmane *	General muscle aging	Muscle healthspan ↑, swimming ability ↑	SKN-1 activation	Maximum velocity: significant improvement	[344]
**Resveratrol**	GMC101, general Aβ models	Chemotaxis restoration, Aβ reduction, lifespan ↑	KEAP1 Cys151 modification, proteasome ↑, proteostasis	Chemotaxis: 10–214% (diet-dependent); Lifespan: 20–30% (OP50 diet)	[349]
Caffeic acid *	VC1772 (*skn-1*(*ok2315*)	Survival ↑, Prevents motor alterations induced by toxins	SKN-1 activation	Lifespan: 28–38% ↑; Body bends per worm: 18–25	[332]
***Canarium album*** **L.**	CL2070, CL2166, TJ356	Survival ↑, antioxidant activity ↑	DAF-16/FOXO and SKN-1/Nrf2 activation	Lifespan: 10.86–15.91% ↑	[334]
Epigallocatechin-3-gallate *	NL2009, CL2166, EG1285, BZ555	Reduce the effect of MeHg-induced lethal, behavioral and neurological toxicity	SKN-1 activation	Reduced ROS and lipid peroxidation: 17.1 and 17.9%; *skn-1* increased 17.3%	[350]
Paeonol *	CF1038, CB1370, TJ356, TJ1052, CF1553, CL2070, CL2166	Survival ↑, oxidative stress reduction, Improved fitness	DAF-16/FOXO and SKN-1/Nrf2 activation	Lifespan: 16.71–28.49% ↑, Lipofuscin 18.32–29.93% ↓; Thermos-resistance: 13.30–22.71% ↓	[333]
Berberine/*Coptis chinensis* Franch *	TJ375, TJ356, CF1553, GR1307, PS3551	Survival ↑, oxidative stress reduction	DAF-16/FOXO and SKN-1/Nrf2 activation	Lifespan: 27% ↑, ROS: 39.4% ↓	[336]
Coffee extract *	CL6222, CL2337, CL6180	Paralysis ↓, Aβ ↓	SKN-1/Nrf2 activation	Aβ 25% ↓	[351]

Note: Efficacy varies significantly based on bacterial diet (live OP50 vs. UV-killed HT115), compound concentration, exposure duration, developmental stage, and temperature. Data represent maximum reported effects under optimized conditions unless otherwise noted. * The cited study employed skn-1 loss-of-function mutants, RNAi or mRNA expression to confirm genetic dependence of the observed effect. The other studies report only correlative changes in Nrf2/SKN-1 expression without genetic validation.

**Table 6:** Worm discovery vs. mammalian validation vs. human evidence.

Compound/Pathway Finding	*C. elegans* Discovery (Worm)	Mammalian Validation (Rodent/In Vivo)	Human Clinical/Observational Evidence
Resveratrol—Nrf2/SKN-1 activation and lifespan/stress resistance	Extends lifespan and stress resistance via SKN-1/DAF-16 activation in *C. elegans* AD models [343]	Reduces oxidative stress and improves cognition in APP/PS1 and other rodent AD models via Nrf2/KEAP1 modulation.	Phase II trial completed (NCT00678431): safe/tolerated, reduced CSF MMP-9; cognitive efficacy not firmly established due to bioavailability limits [352,353]
Curcumin—multi-pathway Nrf2/SKN-1 stimulation	Activates Nrf2/SKN-1 and reduces Aβ-induced toxicity in transgenic *C. elegans* strains [329]	Reduces Aβ burden and neuroinflammation in APP/PS1 and other AD mouse models.	Phase II/III ongoing (NCT03085680); early signals of cognitive stabilization, efficacy data pending [328]
Sulforaphane—KEAP1-Nrf2 modification, anti-inflammatory effect	Activates SKN-1-dependent antioxidant response; protective in Aβ-expressing *C. elegans* strains.	Reduces Aβ burden and improves cognition in APP/PS1 mice; reduces microglial IL-1β, IL-6, iNOS [294,354]	Two ongoing trials (NCT04213391, NCT05233579) in prodromal/MCI-AD and premutation carriers; results pending [355,356]
Vitamin B6 (pyridoxine)—homocysteine/Nrf2-HO-1 link	Not directly modeled in *C. elegans* AD strains (mechanistic inference from mammalian data only).	Homocysteine-lowering and Nrf2/HO-1 upregulation demonstrated in mammalian systems.	Completed RCT (NCT00056225): reduced homocysteine and upregulated Nrf2/HO-1 pathway [357]
DL-3-n-Butylphthalide (NBP)—Nrf2 activation plus vascular/mitochondrial effects	No direct *C. elegans* data reported; compound developed from a plant-derived precursor (celery seed).	Improves synaptic plasticity, vascular cognitive impairment, and antioxidant defense in APP/PS1 mice [358]	Phase II/III ongoing (NCT02993367); approved in China for ischemic stroke (2002), AD efficacy not yet established.
Anti-amyloid monoclonal antibodies + Nrf2 combination (aducanumab, donanemab, lecanemab)	No *C. elegans* equivalent (antibody-based mechanism not modeled in the worm).	Synergistic cognitive benefit reported when combined with Nrf2 activators in mouse AD models [359]	Aducanumab (EMERGE NCT02484547, ENGAGE NCT02477800), donanemab (TRAILBLAZER-ALZ2 NCT04437511), lecanemab (Clarity AD NCT03887455)—FDA approved for amyloid clearance, but not tested in combination with Nrf2 activators in humans
SKN-1/Nrf2 tissue-specific, cell non-autonomous signaling	Intestinal SKN-1 regulates neuromuscular junction function and neuronal stress resistance [309]	Intestinal epithelial Nrf2 modulates systemic antioxidant defense and inflammation in rodents [309]	No direct human observational evidence identified; remains a mechanistic hypothesis for systemic neuroprotection

## Data Availability

No new data were created or analyzed in this study. Data sharing is not applicable to this article.

## Abbreviations

The following abbreviations are used in this manuscript:

AD	Alzheimer’s disease
Aβ plaques	Amyloid β plaques
APP	Amyloid precursor protein
AICD	APP intracellular domain
BACE-1	β-secretase
CTFβ	C-terminal membrane-bound fragment β
*presenilin 1*	*PSEN1*
CDK5	Cyclin-dependent kinase 5
DNMT	DNA methyltransferase
GSK-3β	Glycogen synthase kinase-3β
HDAC	Histone deacetylase
AMPK	Adenosine monophosphate-activated protein kinase
PKA	Cyclic adenosine monophosphate-dependent protein kinase
TPKI	Tau protein kinase I
PRR	Pattern recognition receptor
ASC	Adaptor apoptosis-associated speck-like protein containing a CARD
MAPK	Activated protein kinase
MMPs	Matrix metalloproteinases
BBB	Blood–brain barrier
PUFAs	Polyunsaturated fatty acids
GPX4	Glutathione peroxidase 4
NLRP3	NLR family pyrin domain containing 3
NOX	NADPH oxidase
MAO	Monoamine oxidase
NMDA	N-methyl-D-aspartic acid
cPLA2α	Phosphorylation of cytosolic A2α
COX	Cytochrome c oxidase
ABAD	Aβ-binding alcohol dehydrogenase
MnSOD	Manganese superoxide dismutase
4-HNE	4-hydroxy-2-nonenal
MDA	Malondialdehyde
NDP52	Nuclear dot protein 52
Nrf2	Nuclear factor-erythroid factor 2
ROS	Reactive oxygen species
RNS	Reactive nitrogen species
sMaf	Musculoaponeurotic fibrosarcoma
CHD6	Chromo-ATPase/helicase DNA-binding protein 6
RAC3	Receptor-associated coactivator 3
NQO1	Quinone oxidoreductase 1
HMOX1	Heme oxygenase 1
GST	Glutathione S-transferase
AKRs	Aldo-keto reductases
ALDH1	Aldehyde dehydrogenase 1
SOD1	Superoxide dismutase 1
STING	Stimulator of interferon genes
PROTACs	Proteolysis-targeting chimeras
